# Global, regional, and national disease burden of pneumoconiosis, chronic obstructive pulmonary disease, tracheal-bronchus-and-lung cancer, and asthma attributable to occupational risks, 1990–2021: a systematic analysis for the global burden of disease study 2021

**DOI:** 10.3389/fpubh.2025.1652216

**Published:** 2025-10-08

**Authors:** Shan Lin, Xuefeng Ding, Xin Dang, Qingyuan Zhan

**Affiliations:** ^1^Department of Respiratory and Critical Care Medicine, Affiliated Hospital of North Sichuan Medical College, Nanchong, Sichuan, China; ^2^Department of Critical Care Medicine, Affiliated Hospital of North Sichuan Medical College, Nanchong, Sichuan, China; ^3^Department of Pulmonary and Critical Care Medicine, Center of Respiratory Medicine, National Center for Respiratory Medicine, China-Japan Friendship Hospital, Beijing, China

**Keywords:** pneumoconiosis, chronic obstructive pulmonary disease, tracheal-bronchus-and-lung cancer, asthma, occupational risks

## Abstract

**Background:**

Occupational risks contribute substantially to the global burden of pneumoconiosis, chronic obstructive pulmonary disease (COPD), tracheal-bronchus-and-lung (TBL) cancer, and asthma; however, comprehensive, up-to-date global, regional, and national estimates remain limited.

**Methods:**

Data from the Global Burden of Disease Study (GBD) 2021 were analyzed to quantify the burden of these diseases attributable to occupational exposure across 204 countries between 1990 and 2021, stratified by sex, age, socio-demographic index (SDI), and geographic region.

**Results:**

In 2021, occupational risks led to 30,546 deaths due to pneumoconiosis, 285,628 deaths due to COPD, 585,451 deaths due to TBL (the highest among the four diseases), and 18,315 deaths due to asthma, with disability-adjusted life years (DALYs) of 1.8, 6.1, 12.6, and 0.4 million, respectively. Male patients exhibited higher burdens of all diseases, reflecting greater exposure to male-dominated industries. Geographically, middle SDI regions had the highest absolute cases for pneumoconiosis and COPD, high-SDI regions for TBL cancer, and low-middle SDI regions for asthma. While age-standardized rates declined for most outcomes over time, the absolute burdens increased owing to population growth and aging, with demographic factors driving up to 80% of the increase in total cases. Health inequities persisted, with lower SDI regions bearing a disproportionate burden, despite modest improvements in absolute inequalities.

**Conclusion:**

These findings highlight the need for strengthened occupational health regulations, targeted interventions in high-risk regions, and policies addressing population dynamics to mitigate the impact of workplace exposure on respiratory health.

## 1 Introduction

Occupational respiratory diseases pose a significant global public health challenge, with workplace exposures to harmful agents substantially contributing to the burden of pneumoconiosis, chronic obstructive pulmonary disease (COPD), tracheal-bronchus-and-lung (TBL) cancer, and asthma (1–3). Among these, pneumoconiosis, a group of interstitial lung diseases caused by the inhalation of mineral dust, such as silica, coal, and asbestos, has long been recognized as a major occupational hazard (4). In 2024, the National Health Commission of China highlighted the high prevalence of pneumoconiosis that accounted for approximately 90% of all reported occupational disease cases nationwide (5). Mining, construction, and manufacturing workers are particularly vulnerable, and the disease often leads to irreversible lung damage, diminished quality of life, and premature death (6). COPD, a progressive disease characterized by persistent airflow limitation, is also strongly linked to occupational exposures. Inhalation of dust, fumes, and chemical vapors in the workplace can exacerbate symptoms and accelerate disease progression (7). A landmark study published in The Lancet in 2007 highlighted the significant role of occupational factors in the onset and advancement of COPD (3, 8). Similarly, TBL cancer, which is the leading cause of cancer-related deaths worldwide, has a well-established occupational dimension. Workplace exposure to carcinogens, such as asbestos, arsenic, and polycyclic aromatic hydrocarbons, markedly increases the risk of lung cancer (9). The International Agency for Research on Cancer has classified multiple occupational exposures as carcinogenic to humans, reaffirming the critical link between the occupational environment and cancer risk (10). Asthma, a chronic inflammatory disease of the airway, can be caused or aggravated by occupational exposure. Substances such as isocyanates, flour dust, and animal proteins trigger occupational asthma (11) that constitutes a substantial proportion of adult-onset cases and often results in reduced work productivity and impaired quality of life (12).

Despite well-documented associations between occupational exposure and respiratory diseases, comprehensive and up-to-date assessments of the global, regional, and national burdens attributable to these risks remain scarce. Existing research has often focused on specific regions or occupational groups (13). For example, some studies have highlighted the high prevalence of pneumoconiosis among miners in certain countries, whereas others have examined the impact of industrial chemicals on COPD in localized manufacturing settings (14). Therefore, there is a pressing need for systematic and longitudinal analyses of evidence-based prevention and intervention strategies (15).

The Global Burden of Disease (GBD) study provides a robust framework for quantifying health losses owing to diseases, injuries, and risk factors across different geographic levels (16). By analyzing data between 1990 and 2021, this study aimed to estimate the burdens of pneumoconiosis, COPD, TBL cancer, and asthma attributable to occupational exposure. These findings aimed to identify high-risk populations and regions, support policy development, and guide the implementation of targeted interventions to mitigate the impact of occupational respiratory diseases (17).

## 2 Methods

### 2.1 Data sources

This study draws on GBD 2021, which compiles mortality, morbidity, and risk-factor data across 204 countries and territories from diverse sources (vital registration, surveys, clinical records, registries, and literature) (15). We extracted occupational exposure and attributable burden estimates for pneumoconiosis, COPD, TBL cancer, and asthma. GBD ensures cross-location comparability using standardized models—CODEm for cause-specific mortality and DisMod-MR 2.1 for nonfatal outcomes—that adjust for underreporting and misclassification and provide 95% uncertainty intervals. Population size and age structure are incorporated via UN Population Division estimates, and results are reported as counts and age-standardized rates per 100,000 using the GBD standard population.

In this study, “occupational risks” were defined according to the GBD 2021 classification. In brief, exposures were categorized as mineral dusts for pneumoconiosis; dust, fumes, and chemical vapors for COPD; occupational carcinogens for TBL cancer; and sensitizing agents for asthma. Specifically, pneumoconiosis was attributed to prolonged exposure to mineral dusts (e.g., silica, coal, asbestos); COPD was linked primarily to workplace exposure to dust, fumes, and chemical vapors; TBL cancer was associated with carcinogens such as asbestos, arsenic, and polycyclic aromatic hydrocarbons; and asthma was related to sensitizers including isocyanates, flour dust, and animal proteins.

The data included deaths, disability-adjusted life years (DALYs), years lived with disability (YLDs), and years of life lost (YLLs) between 1990 and 2021.

Country-level socio-demographic index (SDI) values, a composite measure of income per capita, education, and fertility rates, were obtained from the GBD 2021 database to stratify the analyses by development status. The geographic classifications followed the GBD regions (54 regions) and SDI quintiles (low, low-middle, middle, high-middle, and high) (18).

### 2.2 Statistical analysis

#### 2.2.1 Burden disaggregation

The disease burden of pneumoconiosis, COPD, TBL cancer, and asthma attributable to occupational risks was stratified by sex, age groups (5-year intervals from 0–4 to ≥95 years), SDI regions, GBD regions, and individual countries in 2021. Age-standardized rates (ASRs) were calculated using the GBD global reference population to enable cross-regional comparisons.

#### 2.2.2 Temporal trend analysis

Linear regression analysis was applied to assess the temporal trends in age-standardized death, DALYs, YLDs, and YLLs rates between 1990 and 2021. The estimated annual percentage changes (EAPCs) with 95% confidence intervals (CIs) were calculated for each trend segment. EAPCs were derived from the regression coefficient (*β*) of the natural log-transformed rates against calendar year: EAPC = 100 × (*e*^*β* − 1). Trends were categorized as increasing (EAPC > 0), decreasing (EAPC < 0), or stable (EAPC = 0).

#### 2.2.3 Forecasting to 2050

The Bayesian age-period-cohort (BAPC) model was employed to project the burden of occupational risk-related pneumoconiosis, COPD, TBL cancer, and asthma up to 2050. Autoregressive Integrated Moving Average (ARIMA) models and Exponential Smoothing (ES) models were used for the sensitivity analysis.

#### 2.2.4 Decomposition analysis

A demographic decomposition approach partitioned the changes in total deaths, DALYs, YLDs, and YLLs from 1990 to 2021 into three components: (1) population growth, (2) aging (population age structure shifts), and (3) epidemiological changes (age-specific rate variations). The analysis followed the formula:

ΔBurden = ΔPopulation + ΔAging + ΔEpidemiology.

The contribution percentages were calculated for each component.

#### 2.2.5 Health inequality assessment

Health inequities across the SDI quintiles and countries were evaluated using the Slope Index of Inequality (SII) and CI. The SII quantified the absolute differences in ASRs between the highest and lowest SDI groups, whereas the CI measured the relative inequality across the full SDI spectrum.

### 2.3 Software and validation

All analyses were conducted using the R statistical software (version 4.2.2; R Foundation for Statistical Computing). Key packages included forecast for time-series modeling, and decomp for decomposition analysis. Ethical approval was waived because this study utilized fully anonymized aggregated GBD data.

## 3 Results

### 3.1 Disease burden in 2021

In 2021, occupational risks contributed substantially to the global burden of pneumoconiosis, COPD, TBL cancer, and asthma. For pneumoconiosis, there were 30,546 deaths (95% uncertainty interval [UI]: 24,722–43,991), corresponding to an age-standardized death rate (ASDR) of 0.35 (95% UI: 0.28–0.50) per 100,000 population. The disease also caused 1,761,566 DALYs (95% UI: 1,420,293–2,248,898), with an age-standardized DALY rate (ASDAR) of 20.75 (95% UI: 16.7–26.5). Of these, 725,527 were YLDs (ASYLDR 8.71; 95% UI: 5.59–12.66) and 1,036,039 were YLLs (ASYLLR 4.66; 95% UI: −0.45–12.42) (Supplementary Tables S1–S4).

For COPD, occupational exposures were responsible for 285,628 deaths (95% UI: 217,606–354,452), yielding an ASDR of 3.36 (95% UI: 2.56–4.15). The total DALYs reached 6,120,478 (95% UI: 4,668,066–7,778,938), with an ASDAR of 70.41 (95% UI: 53.84–89.37). These were mainly driven by YLLs (6,042,447 cases), while YLDs contributed a smaller share (78,031 cases, ASYLDR 0.91; 95% UI: 0.60–1.26) (Supplementary Tables S5–S8).

For TBL cancer, occupational risks accounted for 585,451 deaths (95% UI: 470,475–715,711), corresponding to an ASDR of 7.03 (95% UI: 5.64–8.67). This represented the highest burden among the four diseases, with 12,601,863 DALYs (95% UI: 10,575,240–14,969,388) and an ASDAR of 147.29 (95% UI: 123–174.54). The burden was primarily from YLLs (10,550,544 cases, ASYLLR 123.5; 95% UI: 101.14–147.67), although YLDs also contributed over two million cases (ASYLDR 23.79; 95% UI: 18.87–28.51) (Supplementary Tables S9–S12).

For asthma, occupational exposures were linked to 18,315 deaths (95% UI: 16,054–20,938), with an ASDR of 0.22 (95% UI: 0.19–0.25). The total DALYs were 446,894 (95% UI: 392,414–512,871), with an ASDAR of 5.22 (95% UI: 4.58–5.97). These were composed of 59,941 YLDs (ASYLDR 0.70; 95% UI: 0.47–0.98) and 386,953 YLLs (ASYLLR 4.52; 95% UI: 3.93–5.25) (Supplementary Tables S13–S16).

When stratified by sex, men consistently carried a higher burden of pneumoconiosis, COPD, TBL cancer, and asthma attributable to occupational risks than women, reflecting greater occupational exposure (Supplementary Tables S1–S16 and Supplementary Figures S1–S4). Age-specific analyses revealed a typical pattern of increasing burden with age, peaking in older adults, followed by a decline (Supplementary Figures S5–S8).

Geographical comparisons showed that middle-SDI regions reported the highest absolute numbers of pneumoconiosis and COPD; high-SDI regions bore the greatest burden of TBL cancer; and low-middle-SDI regions carried the largest burden of asthma. However, patterns of ASRs differed, highlighting the influence of demographic structure and exposure intensity (Supplementary Figures S9–S12).

Finally, significant heterogeneity was observed among the 54 GBD regions and 204 countries, emphasizing substantial differences in occupational exposures and disease outcomes worldwide. Detailed estimates for each region and country are provided in Supplementary Figures S13–S20.

### 3.2 Temporal trend between 1990 and 2021

Between 1990 and 2021, ASRs of pneumoconiosis, COPD, TBL cancer, and asthma attributable to occupational risks generally showed a declining trend, with the exception of ASYLDRs for TBL cancer and asthma, which remained stable or slightly increased. For pneumoconiosis, decreases were observed in DALYs, YLDs, and YLLs, whereas other indicators showed upward trends across the four diseases (Supplementary Figures S21–S24 and Supplementary Tables S1–S16).

When stratified by sex and age, the temporal patterns were broadly consistent with the global trend, with both men and women, as well as most age groups, showing gradual declines in ASRs across all four diseases (Supplementary Figures S25–S32). Similarly, across SDI regions, the overall patterns remained comparable, though the magnitude of the decline varied (Supplementary Figures S33–S36).

Marked regional heterogeneity was observed. For example, ASRs of pneumoconiosis declined substantially in East Asia, South Asia, and most African regions, but showed increases in Oceania and Australasia. COPD-related burdens decreased across much of Europe and high-income Asia Pacific, while rising trends were observed in East Asia and parts of the Western Pacific. TBL cancer demonstrated sustained increases in several Asian and Middle Eastern regions, contrasting with declining burdens in North America and parts of Europe. Asthma showed decreasing ASRs in many low- and middle-income regions but increasing burdens in Oceania and high-income North America (Supplementary Figures S37–S40).

At the national level, additional variations were evident, with some countries experiencing accelerated declines, while others continued to show rising trends. Detailed country-specific trajectories are provided in Supplementary Figures S41–S44 and Supplementary Tables S1–S16.

### 3.3 Predicted results between 2022 and 2050

Regarding the projections of the BAPC, ARIMA, and ES models from 2022 to 2050, a consistent upward trend is observed in the number of cases for both sexes. However, for the corresponding ASRs, the trends are either relatively stable or decreasing (Supplementary Figures S45–S56). Globally, COPD-related deaths attributable to occupational risks are projected to rise from about 285,000 in 2021 to more than 400,000 by 2050 (95% UI: 350,000–460,000). Over the same period, deaths from TBL cancer linked to occupational exposures are expected to increase from roughly 585,000 to over 800,000 (95% UI: 720,000–910,000). Similarly, asthma-related DALYs attributable to occupational exposures are anticipated to grow from 0.45 million in 2021 to approximately 0.60 million by 2050 (95% UI: 0.52–0.70 million).

It is important to note that the predicted increases in absolute numbers of COPD- and TBL cancer–related deaths, as well as asthma-related DALYs, may not solely reflect current occupational environments but also the long latency periods characteristic of many occupational diseases. For example, tracheal, bronchus, and lung cancer can take up to three decades to develop after carcinogenic exposure. As such, part of the projected future burden likely represents the cumulative effects of historic exposures, particularly in regions with a legacy of mining, asbestos use, or other high-risk industries. This latency implies that even if occupational exposure controls are strengthened today, measurable reductions in disease burden may only become evident after several decades. Conversely, regions that industrialized more recently may still experience rising burdens despite improvements in regulation, as prior exposures continue to manifest as disease outcomes over the projection horizon.

### 3.4 Decomposition analysis

**Figure 1:**
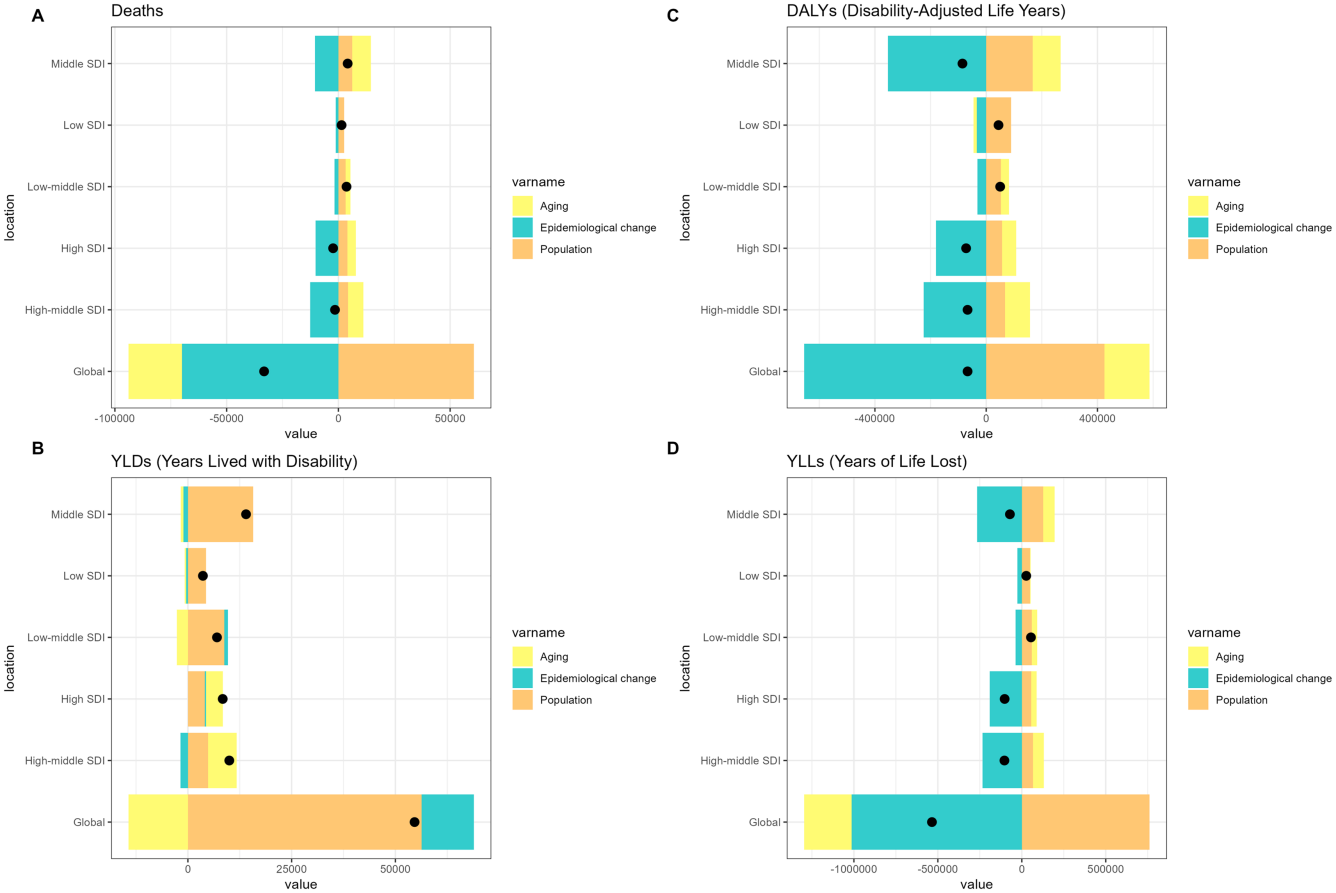
Alterations in the number of cases of pneumoconiosis attributable to occupational risks based on the population-level determinants of population growth, aging, and epidemiological alteration between 1990 and 2021 at the global level and by SDI quintile. SDI, socio-demographic index.

**Figure 2:**
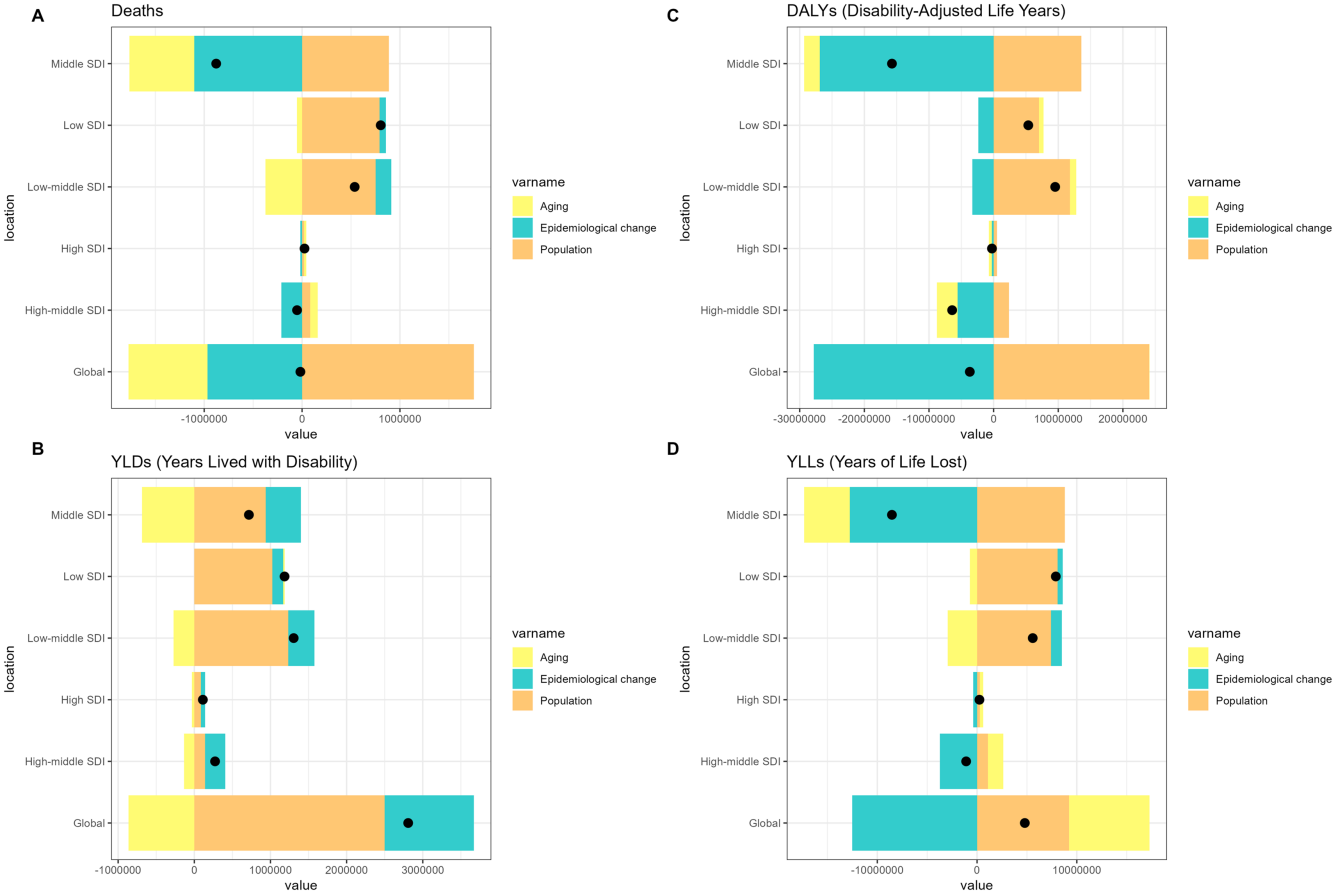
Alterations in the number of cases of chronic obstructive pulmonary disease attributable to occupational risks based on the population-level determinants of population growth, aging, and epidemiological alteration between 1990 and 2021 at the global level and by SDI quintile. SDI, socio-demographic index.

**Figure 3:**
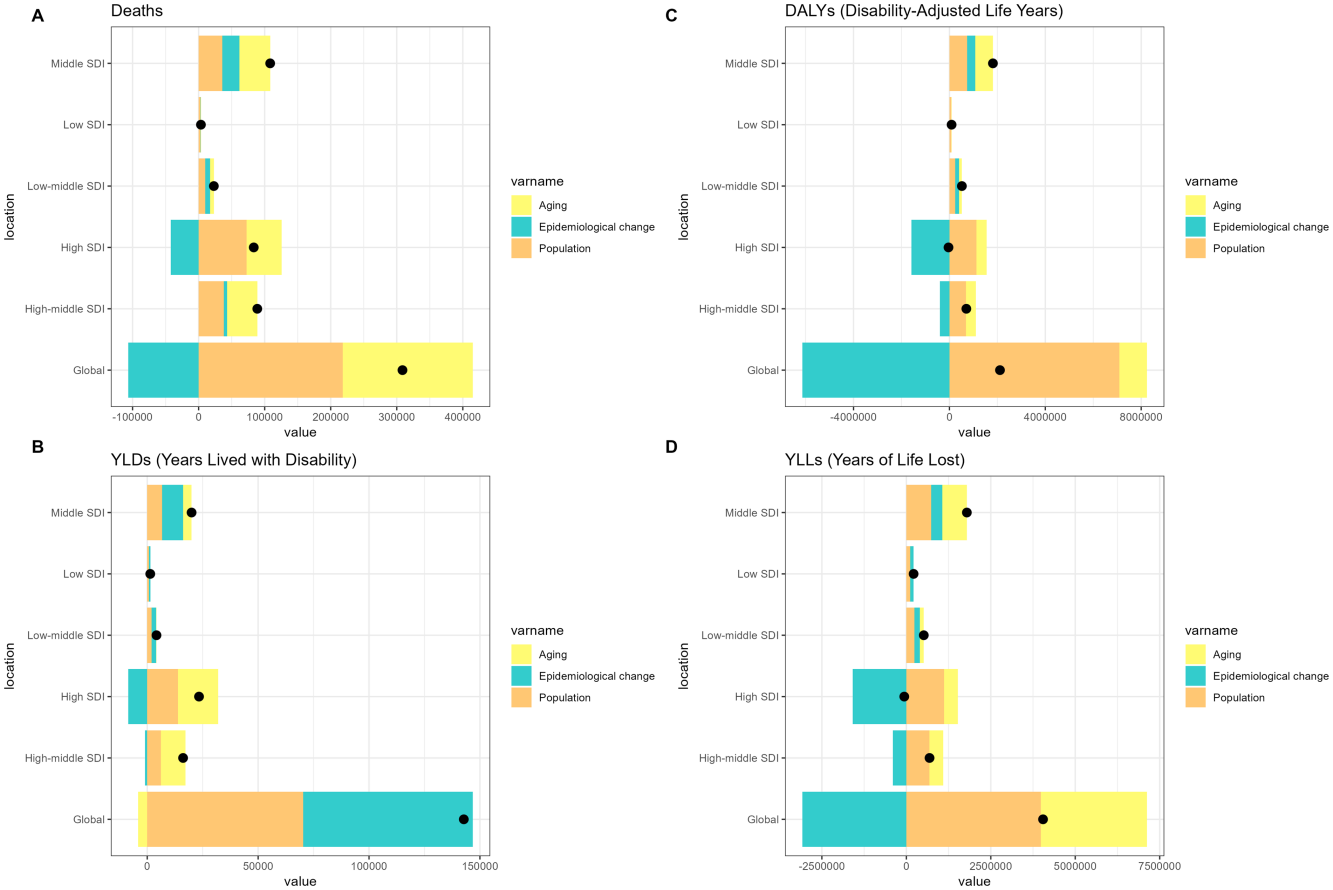
Alterations in the number of cases of tracheal-bronchus-and-lung cancer attributable to occupational risks based on the population-level determinants of population growth, aging, and epidemiological alteration between 1990 and 2021 at the global level and by SDI quintile. SDI, socio-demographic index.

**Figure 4:**
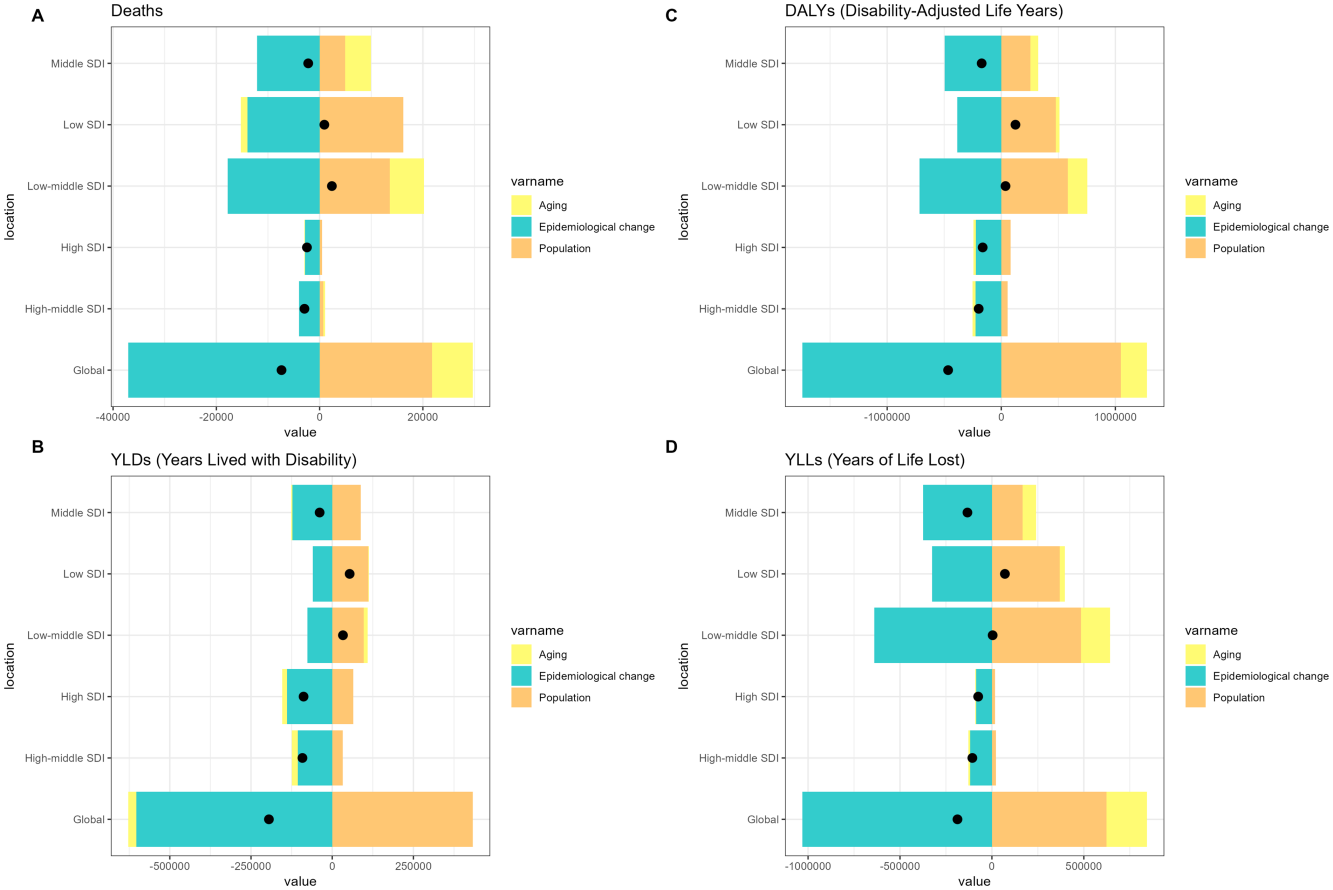
Alterations in the number of cases of asthma attributable to occupational risks based on the population-level determinants of population growth, aging, and epidemiological alteration between 1990 and 2021 at the global level and by SDI quintile. SDI, Socio-demographic index.

Over the past 31 years, a significant global increase in the burden of pneumoconiosis, COPD, TBL cancer, and asthma attributable to occupational risk has been observed. This increase was predominantly fueled by positive population growth and was partially mitigated by negative shifts in epidemiological transitions and aging (Figures 1–4).

### 3.5 Health inequality analysis

Globally, the ASRs burden of pneumoconiosis, COPD, TBL cancer, and asthma attributable to occupational risks is primarily concentrated in areas with lower SDI. Between 1990 and 2021, the SII demonstrated an improvement in inequality among lower SDI countries. In contrast, the concentration index of ASRs showed a relatively stable trend, reflecting a consistent burden concentration in countries with lower SDI (Supplementary Figures S57–S60).

## 4 Discussion

This study provides a comprehensive analysis of the global, regional, and national burdens of pneumoconiosis, COPD, TBL cancer, and asthma attributable to occupational risks between 1990 and 2021. In 2021, occupational risks contributed to a substantial disease burden, with TBL cancer accounting for the highest number of deaths (585,451; DALYs, 12,601,863), followed by COPD (285,628 deaths; 6,120,478 DALYs). Pneumoconiosis, which causes fewer deaths (30,546), had a high burden of YLDs (725,527), reflecting its irreversible nature. Asthma attributable to occupational risks had the lowest mortality rate (18,315 deaths), but remained a significant cause of morbidity. Male patients consistently bore a higher burden across all diseases because of the historically higher exposure in male-dominated industries (19). Geographically, the middle SDI regions had the highest absolute burden of pneumoconiosis and COPD, the high-SDI regions reported the highest TBL cancer burden, and the low-middle SDI regions had the highest asthma burden. Temporal trends showed declining ASRs for most outcomes; however, absolute numbers increased owing to population growth and aging, highlighting the need for sustained interventions (20).

Our findings are broadly consistent with prior epidemiological evidence. We observed a substantial occupational burden of COPD, echoing earlier systematic reviews that identify workplace exposures as key contributors to COPD onset and progression (21, 22). The high prevalence of pneumoconiosis in China and India likewise accords with recent national reports, which note that pneumoconiosis constitutes over 90% of all occupational diseases in China (23, 24). Notable discrepancies also emerged. Although our projections indicate continued growth in TBL cancer cases through 2050, other studies have documented declining trends in asbestos-related outcomes, which may reflect differences in modeling strategies and time horizons (25, 26). In addition, while we estimate a decreasing burden of occupational asthma in select low-income regions, previous work reports rising asthma prevalence in Africa (27). Such divergences likely stem from heterogeneity in data sources, surveillance capacity, and whether demographic dynamics are explicitly incorporated into the modeling framework. Overall, by leveraging the GBD 2021 framework, our study provides the most up-to-date global estimates and, in several respects, complements and extends the existing literature.

The dominance of TBL cancer in high-SDI regions aligns with the historical exposure to well-characterized carcinogens, such as asbestos and arsenic, in industrialized settings (28, 29). For example, a 2019 study in Environmental Health Perspectives linked long-term occupational carcinogen exposure in North America and Western Europe to an elevated lung cancer risk, consistent with our findings of stable to increasing ASRs in these regions despite stricter regulations (29). In contrast, the high burden of pneumoconiosis in middle SDI regions, such as China and India, indicates inadequate dust control in mining and construction industries (30, 31). China’s National Health Commission noted that pneumoconiosis accounts for 90% of the occupational diseases in the country, a finding reflected in our data showing a peak ASRs in East Asia (30).

The COPD attributable to occupational risks demonstrated a complex pattern, while high-SDI regions, such as Australasia saw declining ASRs, upper-middle SDI regions (Eastern Europe) experienced increases, possibly because of persistent exposure to coal dust and chemical fumes in transitioning economies (32, 33). The Lancet highlighted occupational factors as key drivers of COPD progression, which our decomposition analysis confirmed, showing that epidemiological changes (improved workplace safety) only partially offset the population growth effects (33).

Occupational asthma, although less lethal, poses a significant burden in low-middle SDI regions, likely due to exposure to organic dust (flour and wood dust) and isocyanates in manufacturing without adequate respiratory protection (34, 35). A recent study associated rising asthma incidence in South Asia with informal sector employment, which is consistent with our findings of high burdens in low-middle SDI clusters (35).

The decline in age-standardized death and DALY rates for most diseases reflects progress in mitigating occupational hazards, primarily through the reduction of workplace exposures to hazardous substances. Key drivers include the global phase-out of asbestos (linked to pneumoconiosis and TBL cancer), implementation of dust-control strategies, stricter occupational health regulations, and structural changes in industry that have shifted workers away from high-exposure sectors (25, 36). Although workplace ventilation improvements may contribute, the critical factor is reduced intensity and duration of exposure to harmful agents. In addition, changes in non-occupational determinants likely influenced disease trends. For example, declining smoking prevalence in some high-SDI countries has reduced COPD incidence, while persistently high smoking rates in low- and middle-SDI regions may have attenuated progress. For TBL cancer, the rising number of YLDs despite relatively stable ASRs suggests improved survival associated with early detection and treatment in high-SDI regions; however, this contrasts with the poorer prognosis in low-SDI settings lacking adequate screening programs (37).

The regional disparities in trend directions are striking. These regional differences likely reflect variation in industrial structure, regulatory enforcement, and the availability of occupational health resources across countries. The decline observed in East Asia, particularly in China, is plausibly related to the recent adoption of stricter exposure control measures and strengthened occupational health legislation. By contrast, the increases in Oceania and Australasia are likely tied to legacy exposures in mining-oriented economies, where workers continue to experience long-term consequences despite improved contemporary standards. In high-SDI regions such as North America and Western Europe, the comparatively high burden of TBL is consistent with historical exposure to asbestos and other carcinogens, although tighter regulations have tempered further growth. Conversely, in low-SDI regions, the persistent burden of occupational asthma may stem from inadequate regulation, limited access to personal protective equipment, and weak health surveillance systems, which together lead to underrecognition and delayed intervention. Oceania and Australasia saw increasing pneumoconiosis ASRs, possibly due to legacy exposures in mining sectors with aging workforces (38), whereas sharp declines in East Asia reflect China’s recent occupational health reforms (30). For asthma, unexpected increases in high-SDI regions such as North America may be related to underdiagnosis in subtle exposure scenarios (laboratory chemicals), whereas declines in sub-Saharan Africa could stem from reduced industrialization or data limitations (27).

The concentration of the disease burden in regions with lower SDI highlights systemic inequities in occupational health protection. SII improvements suggest a narrowing of absolute gaps, but stable CIs indicate persistent relative disparities. This mirrors global trends in occupational health, where low-income countries lack regulatory frameworks and surveillance systems, leading to higher exposure to unregulated hazards (39, 40). Interventions must prioritize resource-constrained regions by combining engineering controls (dust extraction), personal protective equipment, and health monitoring (41). The International Labor Organization emphasizes that improving workplace safety could prevent 86% of occupational lung disease deaths, underscoring the cost-effectiveness of policy action (42).

This study has few limitations that warrant consideration. First, the reliance on GBD data assumes accurate quantification of occupational exposure, which may be underreported in regions with limited surveillance, particularly for niche hazards, such as rare industrial chemicals (43). Second, our analysis aggregated diverse occupational risks (dust, fumes, and carcinogens), potentially obscuring hazard-specific contributions (44). Third, projections for 2050 assume stable socioeconomic and policy environments, which may not account for future technological advancements or regulatory shifts (global asbestos bans) (45). Finally, sex disparity analysis, while showing male predominance, did not explore sex-specific exposure pathways (maternal occupational risks affecting offspring asthma) highlighting a gap for future research (46).

## 5 Conclusion

This study highlights the enduring global burden of occupational respiratory diseases with distinct regional and demographic patterns. While ASRs have declined in many areas, population growth and aging threaten to exacerbate absolute burdens, particularly in the middle and low-middle SDI regions. Addressing these disparities requires coordinated action, including strengthening occupational health regulations in developing economies, investing in exposure monitoring, and promoting sex-sensitive interventions. The GBD framework provides a critical tool for prioritization, but sustained political will and international collaboration are essential to translate evidence into actionable policies and reduce preventable workplace risks.

## Data Availability

The original contributions presented in the study are included in the article/Supplementary material, further inquiries can be directed to the corresponding authors.
