# Supplementary material for: Global, regional, and national disease burden of pneumoconiosis, chronic obstructive pulmonary disease, tracheal-bronchus-and-lung cancer, and asthma attributable to occupational risks, 1990–2021: a systematic analysis for the global burden of disease study 2021
Source: Front Public Health. 2025 Oct 8;13:1652216. doi: 10.3389/fpubh.2025.1652216 (PMC12540489; doi:10.3389/fpubh.2025.1652216)
Supplement: Supplementary file 1 [file Supplementary_file_1.docx]

**Table legends**

**Table S1.** The number of deaths cases and the age-standardized deaths rate of pneumoconiosis attributable to occupational risks in 1990 and 2021, and its trends from 1990 to 2021 globally.

**Table S2.** The number of DALYs cases and the age-standardized DALYs rate of pneumoconiosis attributable to occupational risks in 1990 and 2021, and its trends from 1990 to 2021 globally. Abbreviations: DALYs, disability-adjusted life years.

**Table S3.** The number of YLDs cases and the age-standardized YLDs rate of pneumoconiosis attributable to occupational risks in 1990 and 2021, and its trends from 1990 to 2021 globally. Abbreviations: YLDs, years lived with disability.

**Table S4.** The number of YLLs cases and the age-standardized YLLs rate of pneumoconiosis attributable to occupational risks in 1990 and 2021, and its trends from 1990 to 2021 globally. Abbreviations: YLLs, years of life lost.

**Table S5.** The number of deaths cases and the age-standardized deaths rate of chronic obstructive pulmonary disease attributable to occupational risks in 1990 and 2021, and its trends from 1990 to 2021 globally.

**Table S6.** The number of DALYs cases and the age-standardized DALYs rate of chronic obstructive pulmonary disease attributable to occupational risks in 1990 and 2021, and its trends from 1990 to 2021 globally. Abbreviations: DALYs, disability-adjusted life years.

**Table S7.** The number of YLDs cases and the age-standardized YLDs rate of chronic obstructive pulmonary disease attributable to occupational risks in 1990 and 2021, and its trends from 1990 to 2021 globally. Abbreviations: YLDs, years lived with disability.

**Table S8.** The number of YLLs cases and the age-standardized YLLs rate of chronic obstructive pulmonary disease attributable to occupational risks in 1990 and 2021, and its trends from 1990 to 2021 globally. Abbreviations: YLLs, years of life lost.

**Table S9.** The number of deaths cases and the age-standardized deaths rate of tracheal-bronchus-and-lung cancer attributable to occupational risks in 1990 and 2021, and its trends from 1990 to 2021 globally.

**Table S10.** The number of DALYs cases and the age-standardized DALYs rate of tracheal-bronchus-and-lung cancer attributable to occupational risks in 1990 and 2021, and its trends from 1990 to 2021 globally. Abbreviations: DALYs, disability-adjusted life years.

**Table S11.** The number of YLDs cases and the age-standardized YLDs rate of tracheal-bronchus-and-lung cancer attributable to occupational risks in 1990 and 2021, and its trends from 1990 to 2021 globally. Abbreviations: YLDs, years lived with disability.

**Table S12.** The number of YLLs cases and the age-standardized YLLs rate of tracheal-bronchus-and-lung cancer attributable to occupational risks in 1990 and 2021, and its trends from 1990 to 2021 globally. Abbreviations: YLLs, years of life lost.

**Table S13.** The number of deaths cases and the age-standardized deaths rate of asthma attributable to occupational risks in 1990 and 2021, and its trends from 1990 to 2021 globally.

**Table S14.** The number of DALYs cases and the age-standardized DALYs rate of asthma attributable to occupational risks in 1990 and 2021, and its trends from 1990 to 2021 globally. Abbreviations: DALYs, disability-adjusted life years.

**Table S15.** The number of YLDs cases and the age-standardized YLDs rate of asthma attributable to occupational risks in 1990 and 2021, and its trends from 1990 to 2021 globally. Abbreviations: YLDs, years lived with disability.

**Table S16.** The number of YLLs cases and the age-standardized YLLs rate of asthma attributable to occupational risks in 1990 and 2021, and its trends from 1990 to 2021 globally. Abbreviations: YLLs, years of life lost.

**Figure legend**

**
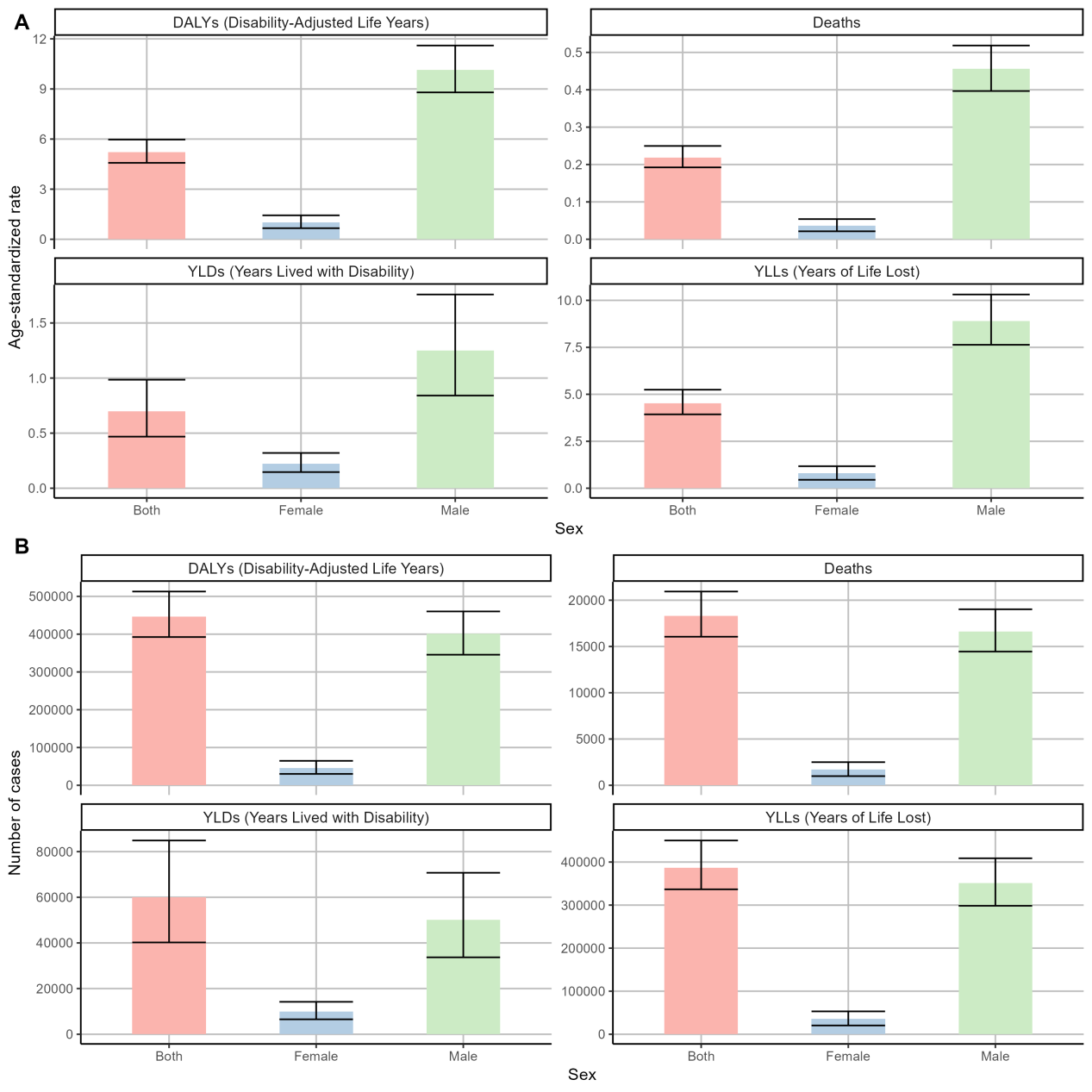
Figure S1.** Numbers and age-standardized rates of pneumoconiosis attributable to occupational risks-related deaths, DALYs, YLDs, and YLLs for both sexes in 2021. Abbreviations: DALYs, disability-adjusted life years; YLDs, years lived with disability; YLLs, years of life lost.

**
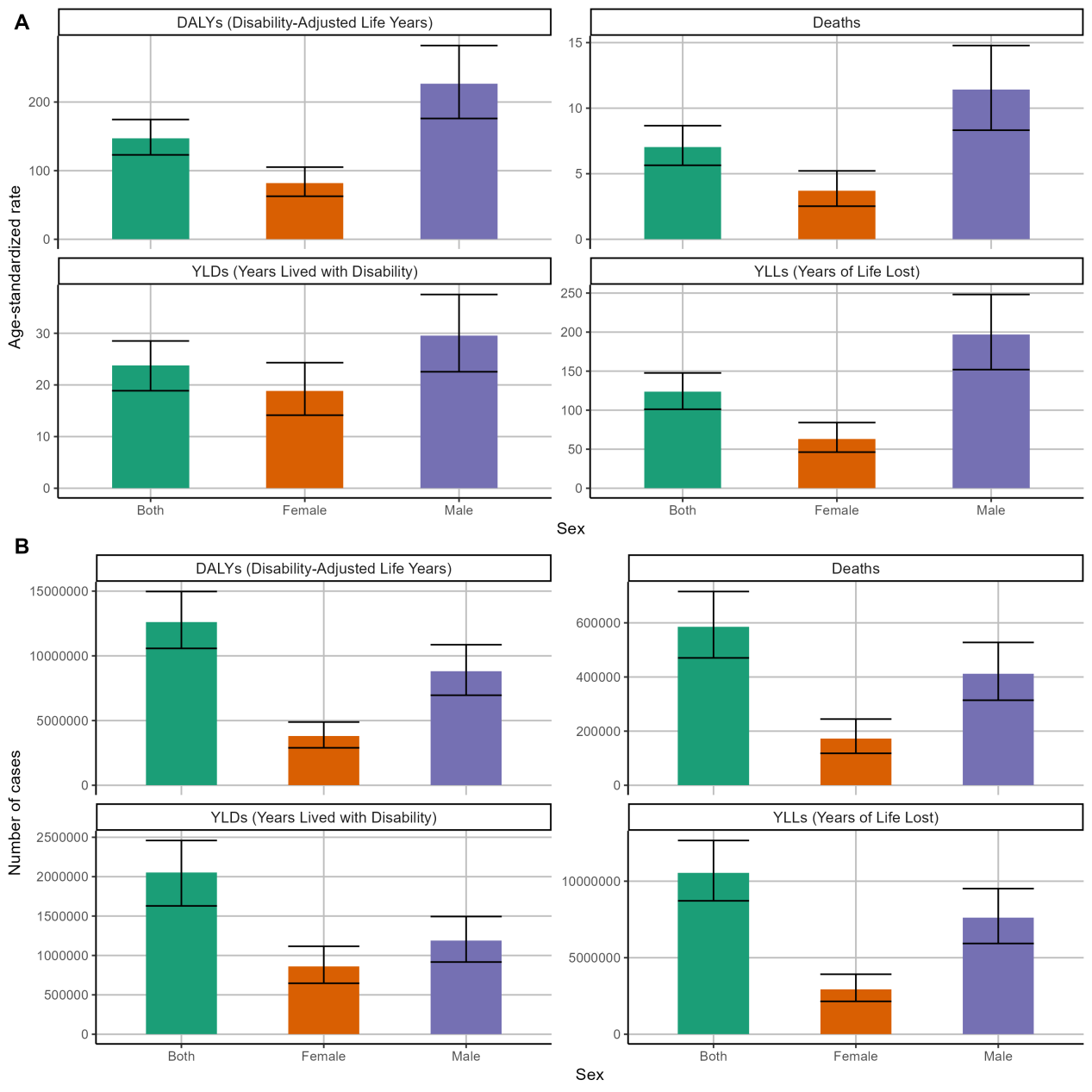
Figure S2.** Numbers and age-standardized rates of chronic obstructive pulmonary disease attributable to occupational risks-related deaths, DALYs, YLDs, and YLLs for both sexes in 2021. Abbreviations: DALYs, disability-adjusted life years; YLDs, years lived with disability; YLLs, years of life lost.

**
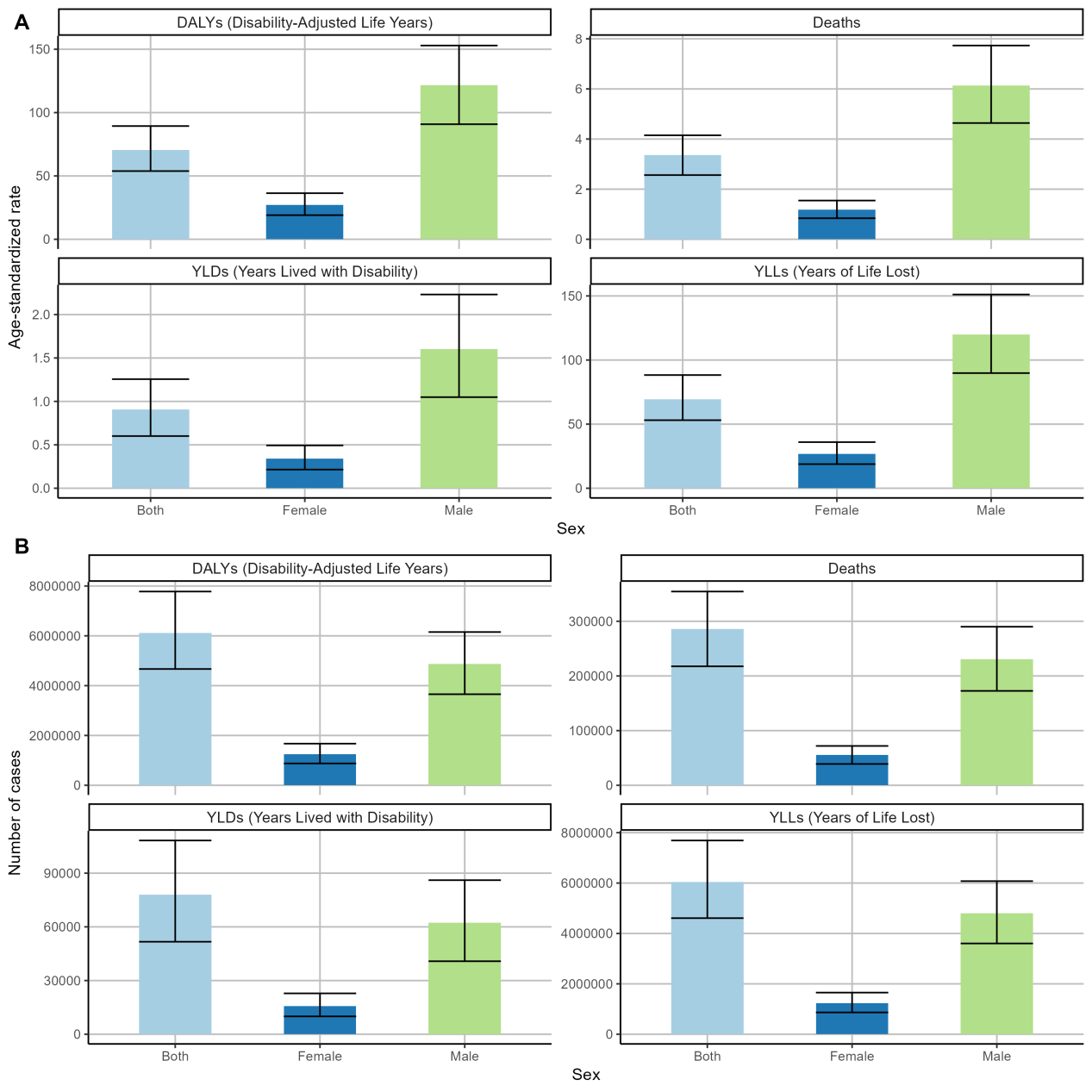
Figure S3.** Numbers and age-standardized rates of tracheal-bronchus-and-lung cancer attributable to occupational risks-related deaths, DALYs, YLDs, and YLLs for both sexes in 2021. Abbreviations: DALYs, disability-adjusted life years; YLDs, years lived with disability; YLLs, years of life lost.

**
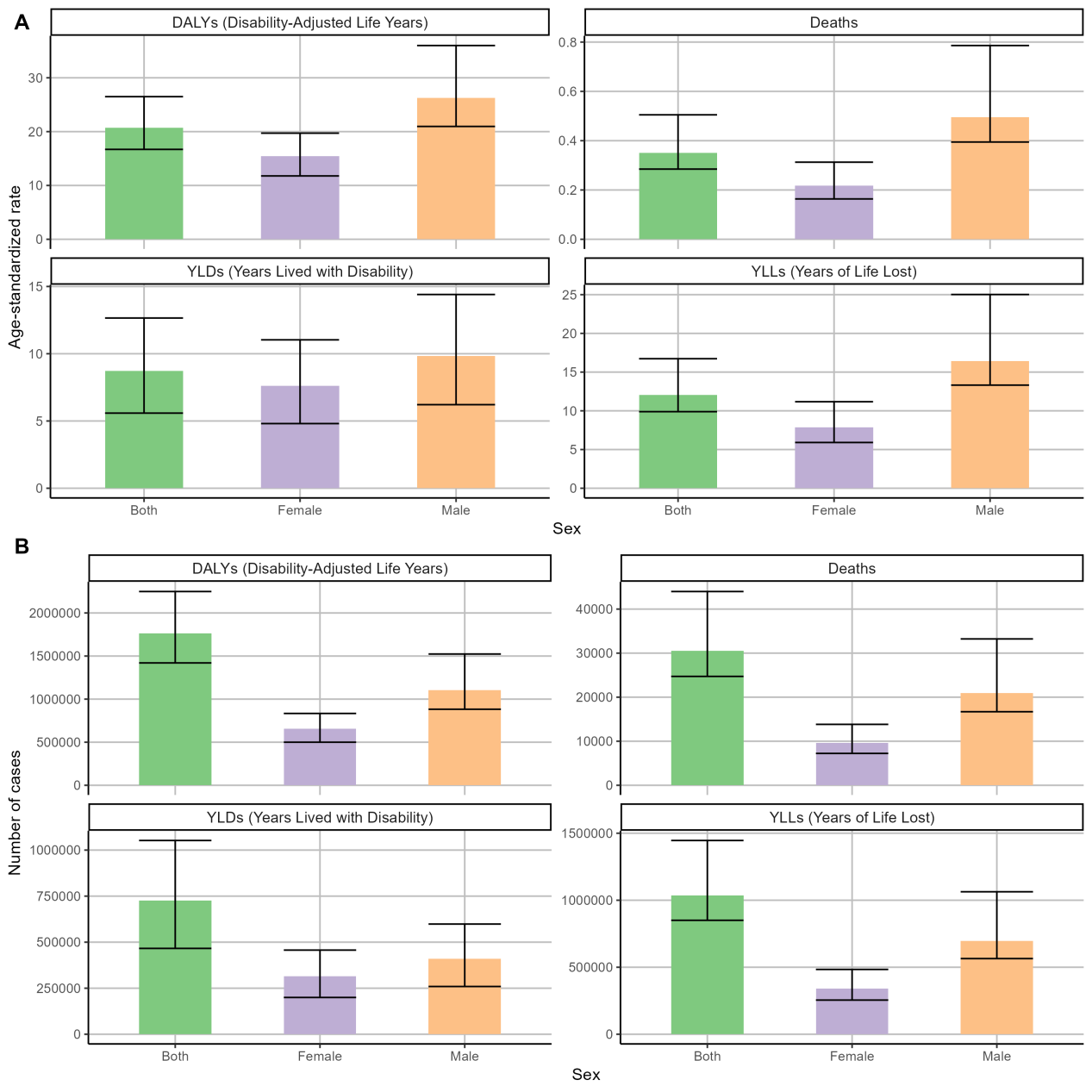
Figure S4.** Numbers and age-standardized rates of asthma attributable to occupational risks-related deaths, DALYs, YLDs, and YLLs for both sexes in 2021. Abbreviations: DALYs, disability-adjusted life years; YLDs, years lived with disability; YLLs, years of life lost.

**
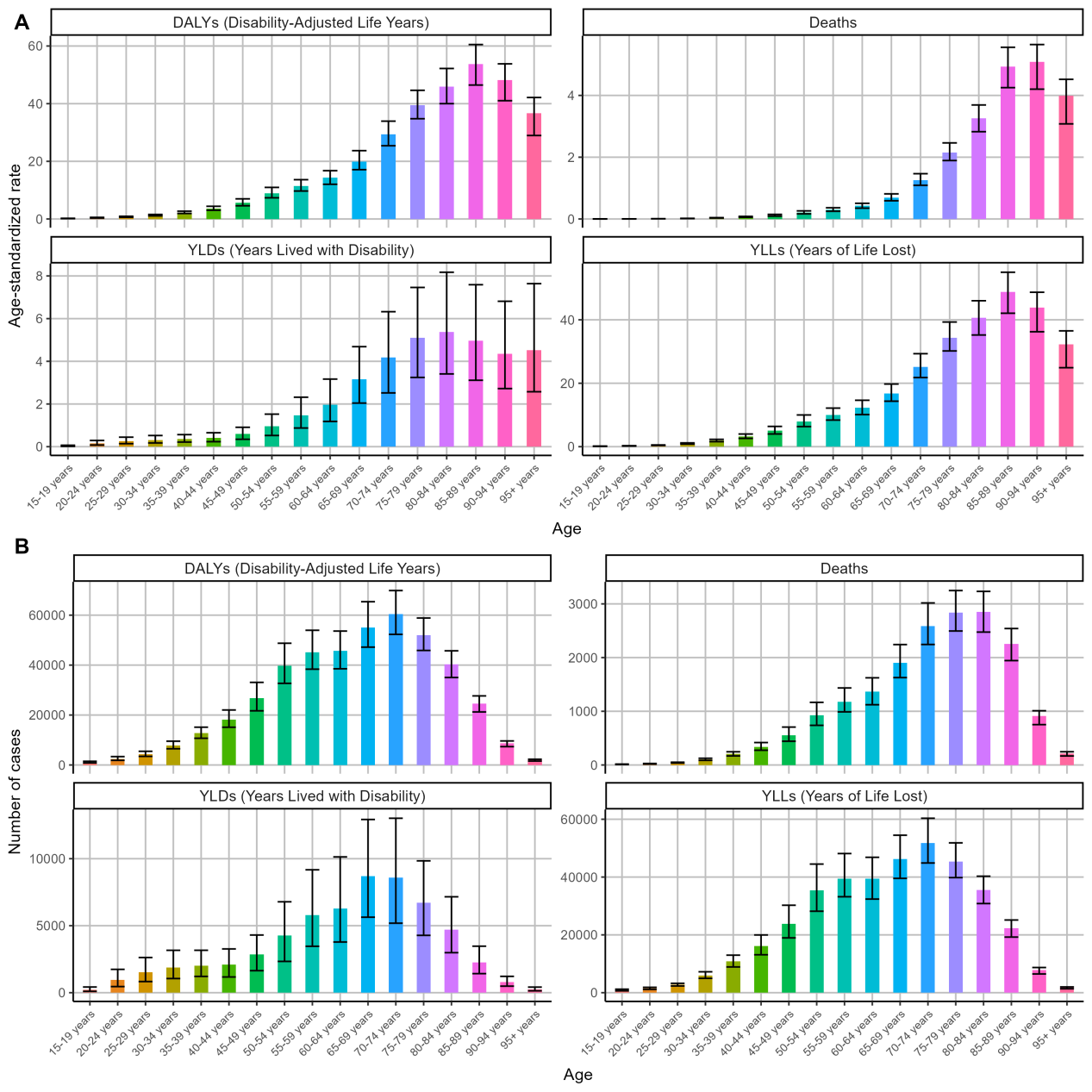
Figure S5.** Numbers and age-standardized rates of pneumoconiosis attributable to occupational risks-related deaths, DALYs, YLDs, and YLLs for different age groups in 2021. Abbreviations: DALYs, disability-adjusted life years; YLDs, years lived with disability; YLLs, years of life lost.

**
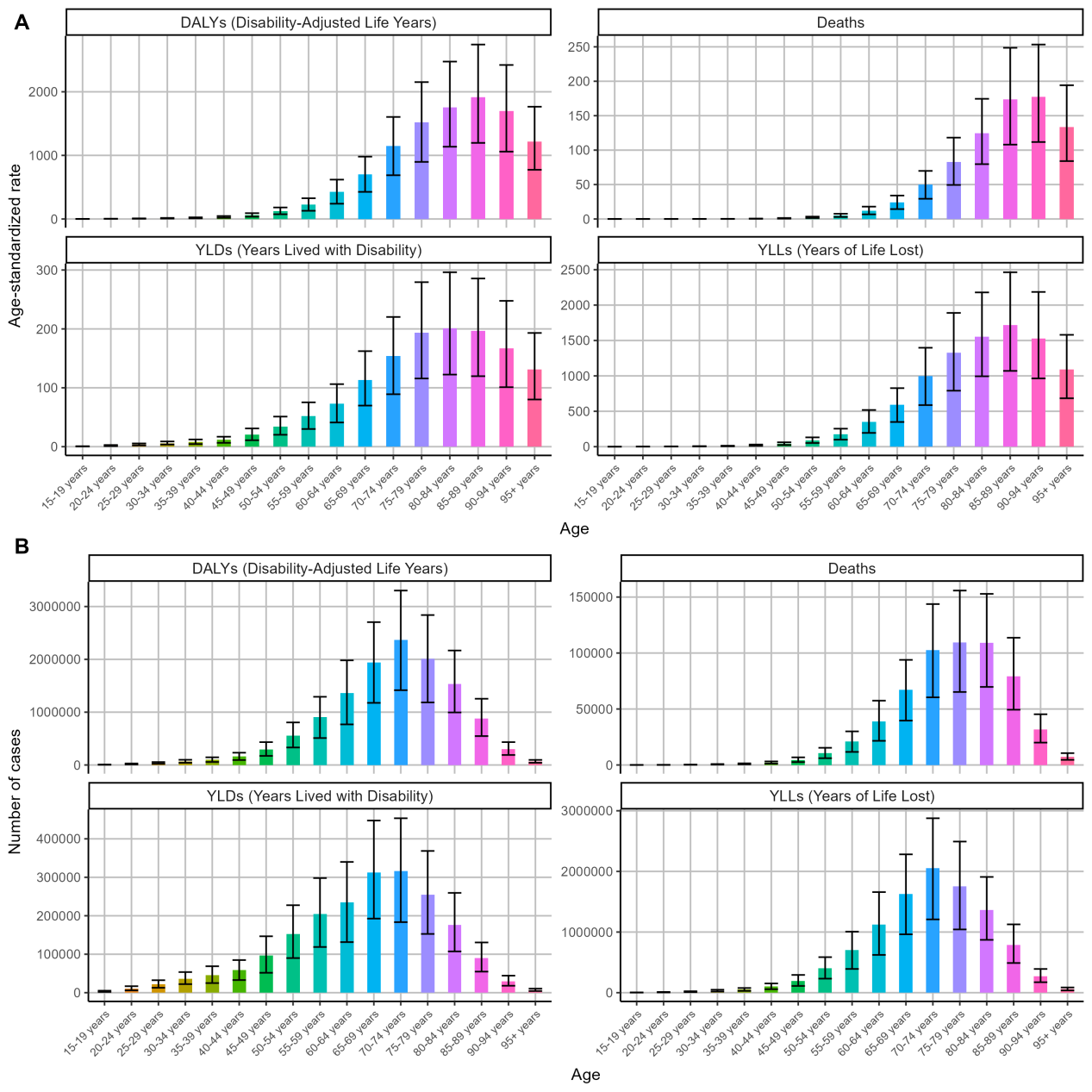
Figure S6.** Numbers and age-standardized rates of chronic obstructive pulmonary disease attributable to occupational risks-related deaths, DALYs, YLDs, and YLLs for different age groups in 2021. Abbreviations: DALYs, disability-adjusted life years; YLDs, years lived with disability; YLLs, years of life lost.

**
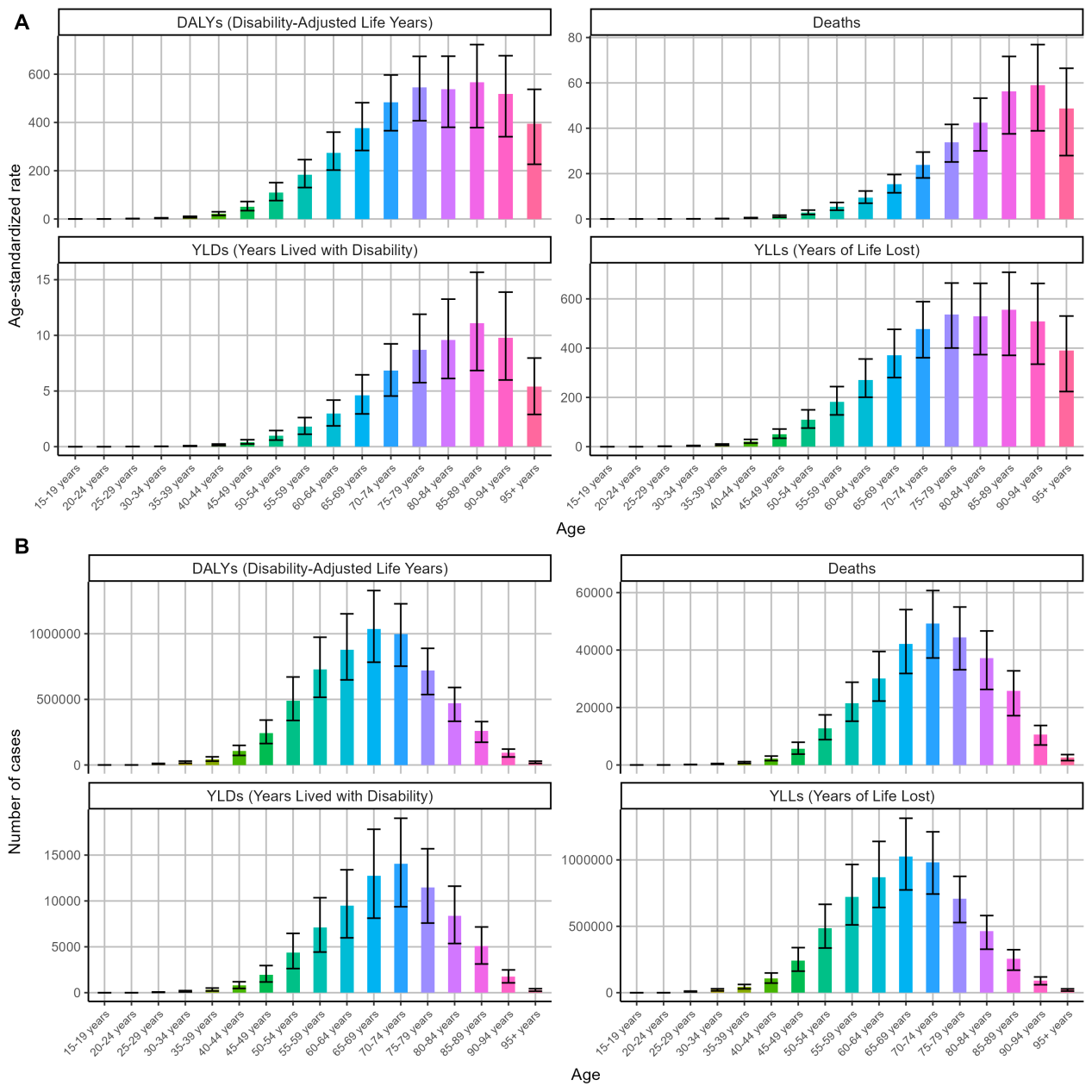
Figure S7.** Numbers and age-standardized rates of tracheal-bronchus-and-lung cancer attributable to occupational risks-related deaths, DALYs, YLDs, and YLLs for different age groups in 2021. Abbreviations: DALYs, disability-adjusted life years; YLDs, years lived with disability; YLLs, years of life lost.

**
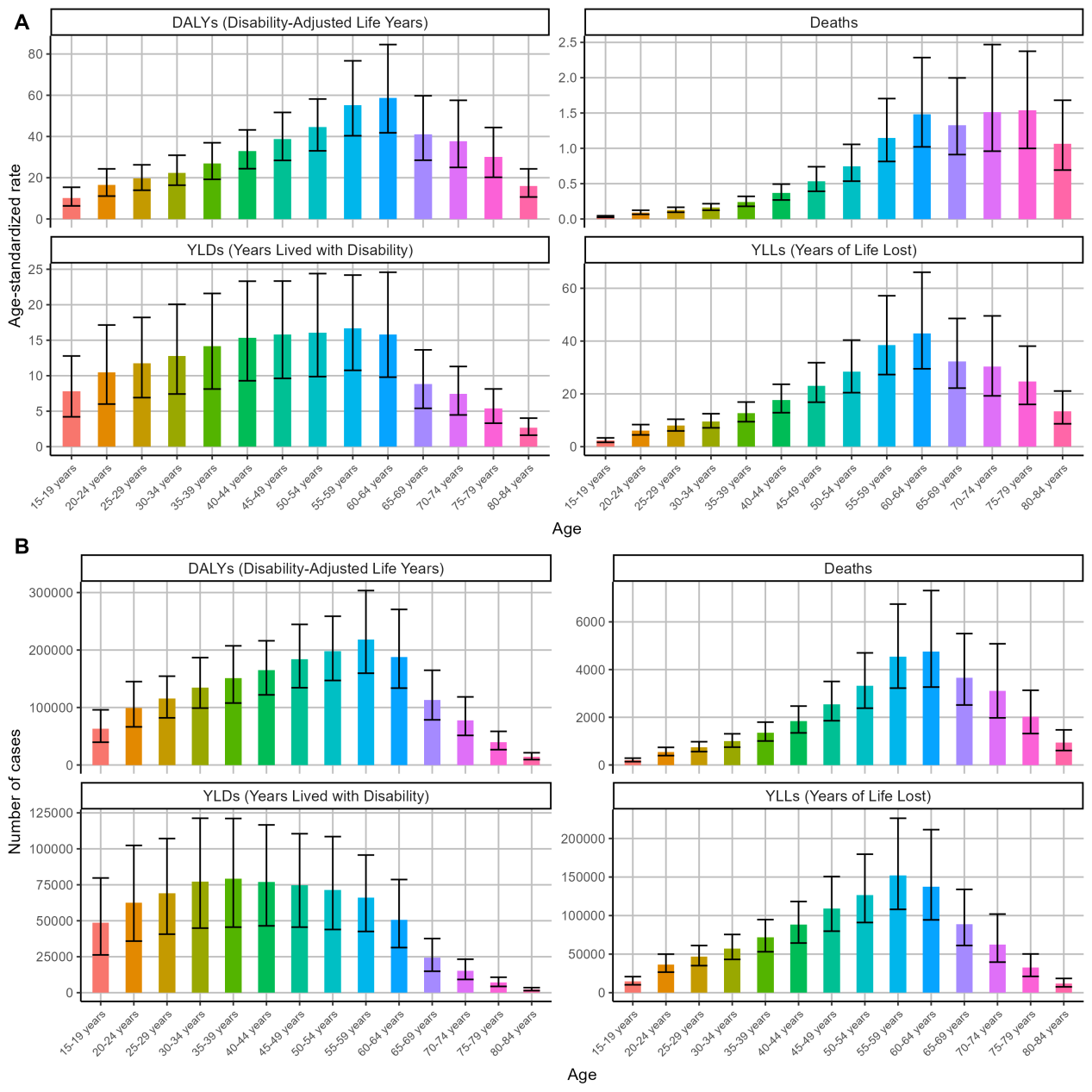
Figure S8.** Numbers and age-standardized rates of asthma attributable to occupational risks-related deaths, DALYs, YLDs, and YLLs for different age groups in 2021. Abbreviations: DALYs, disability-adjusted life years; YLDs, years lived with disability; YLLs, years of life lost.

**
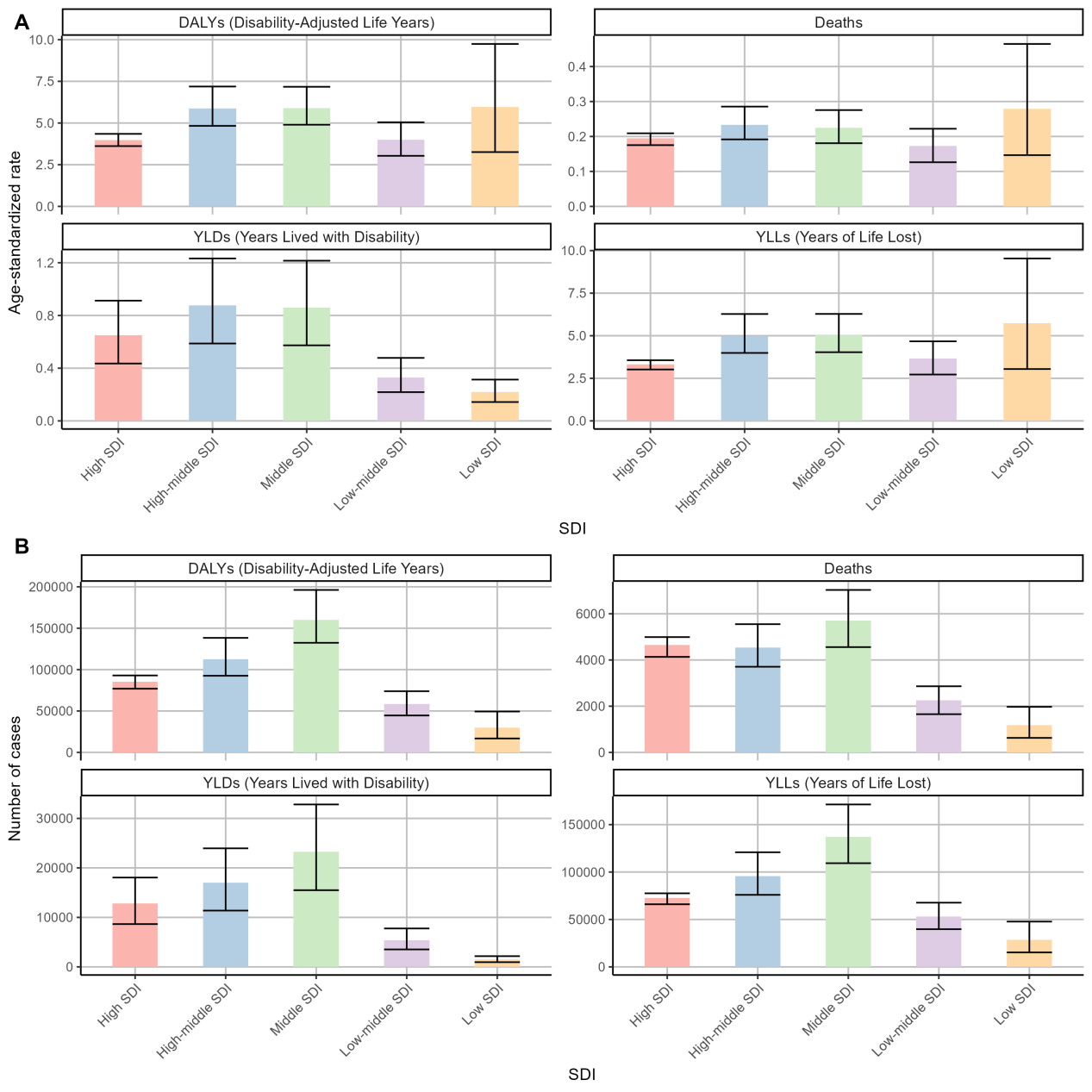
Figure S9.** Numbers and age-standardized rates of pneumoconiosis attributable to occupational risks-related deaths, DALYs, YLDs, and YLLs for different SDI regions in 2021. Abbreviations: DALYs, disability-adjusted life years; YLDs, years lived with disability; YLLs, years of life lost; SDI, Socio - demographic Index.

**
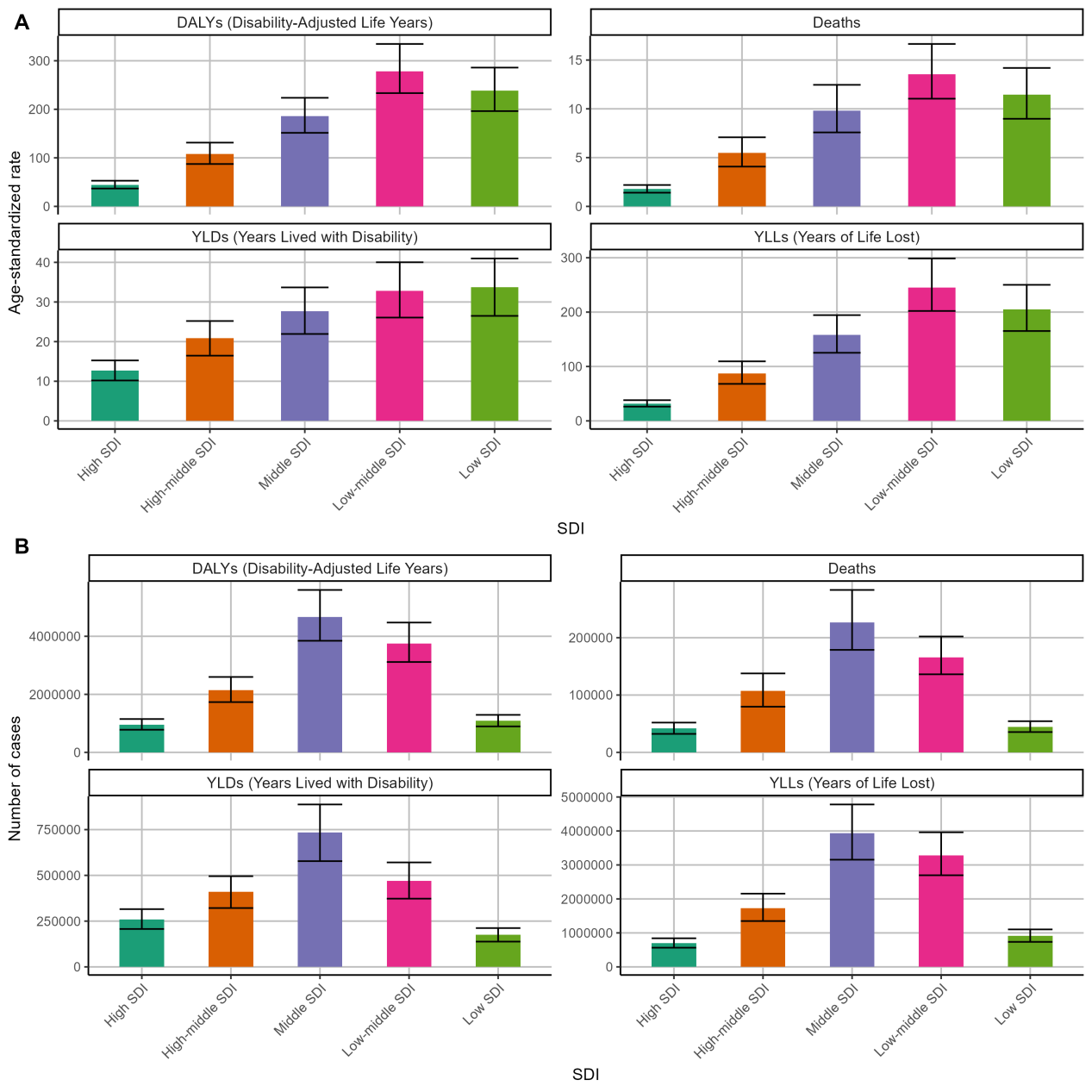
Figure S10.** Numbers and age-standardized rates of chronic obstructive pulmonary disease attributable to occupational risks-related deaths, DALYs, YLDs, and YLLs for different SDI regions in 2021. Abbreviations: DALYs, disability-adjusted life years; YLDs, years lived with disability; YLLs, years of life lost; SDI, Socio - demographic Index.

**
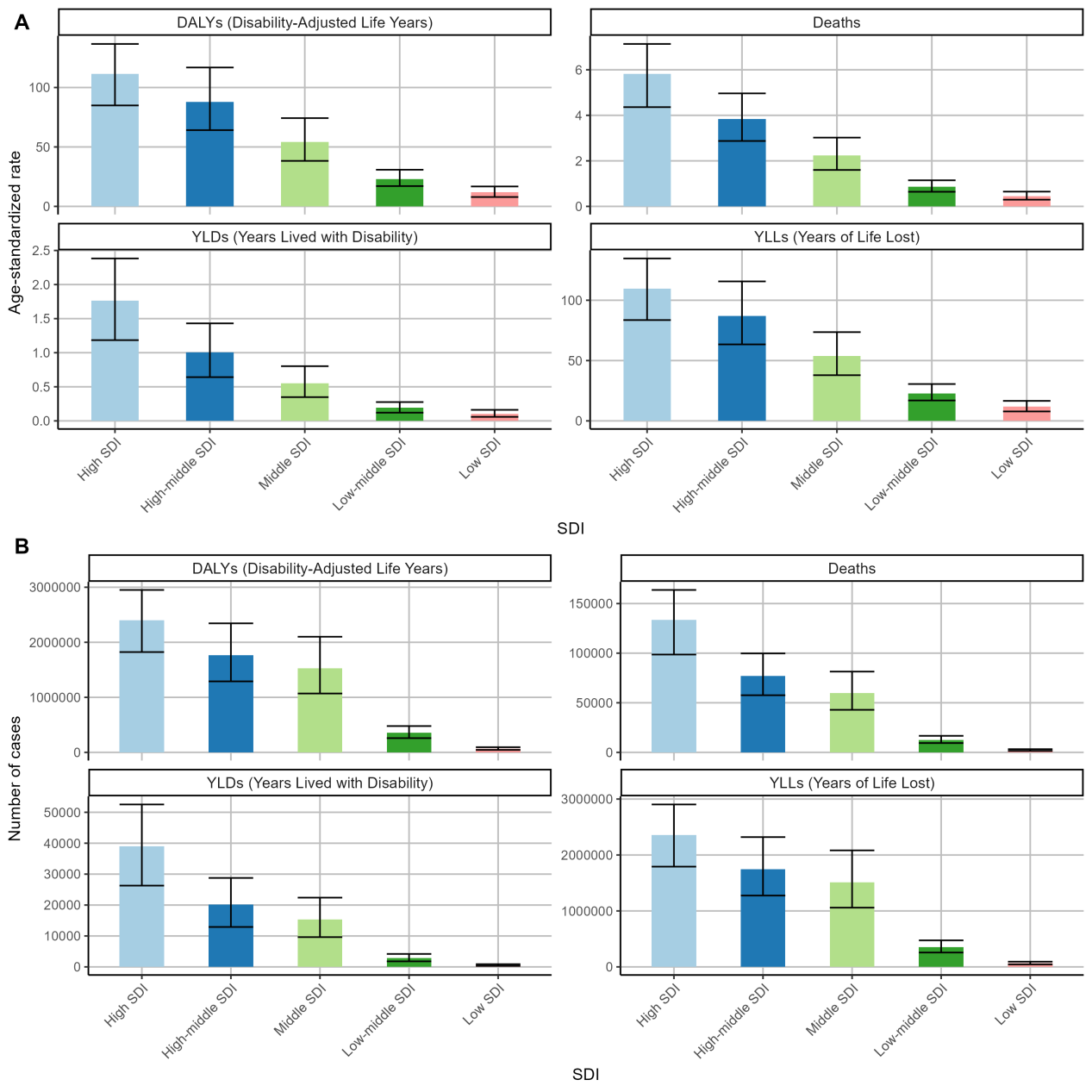
Figure S11.** Numbers and age-standardized rates of tracheal-bronchus-and-lung cancer attributable to occupational risks-related deaths, DALYs, YLDs, and YLLs for different SDI regions in 2021. Abbreviations: DALYs, disability-adjusted life years; YLDs, years lived with disability; YLLs, years of life lost; SDI, Socio - demographic Index.

**
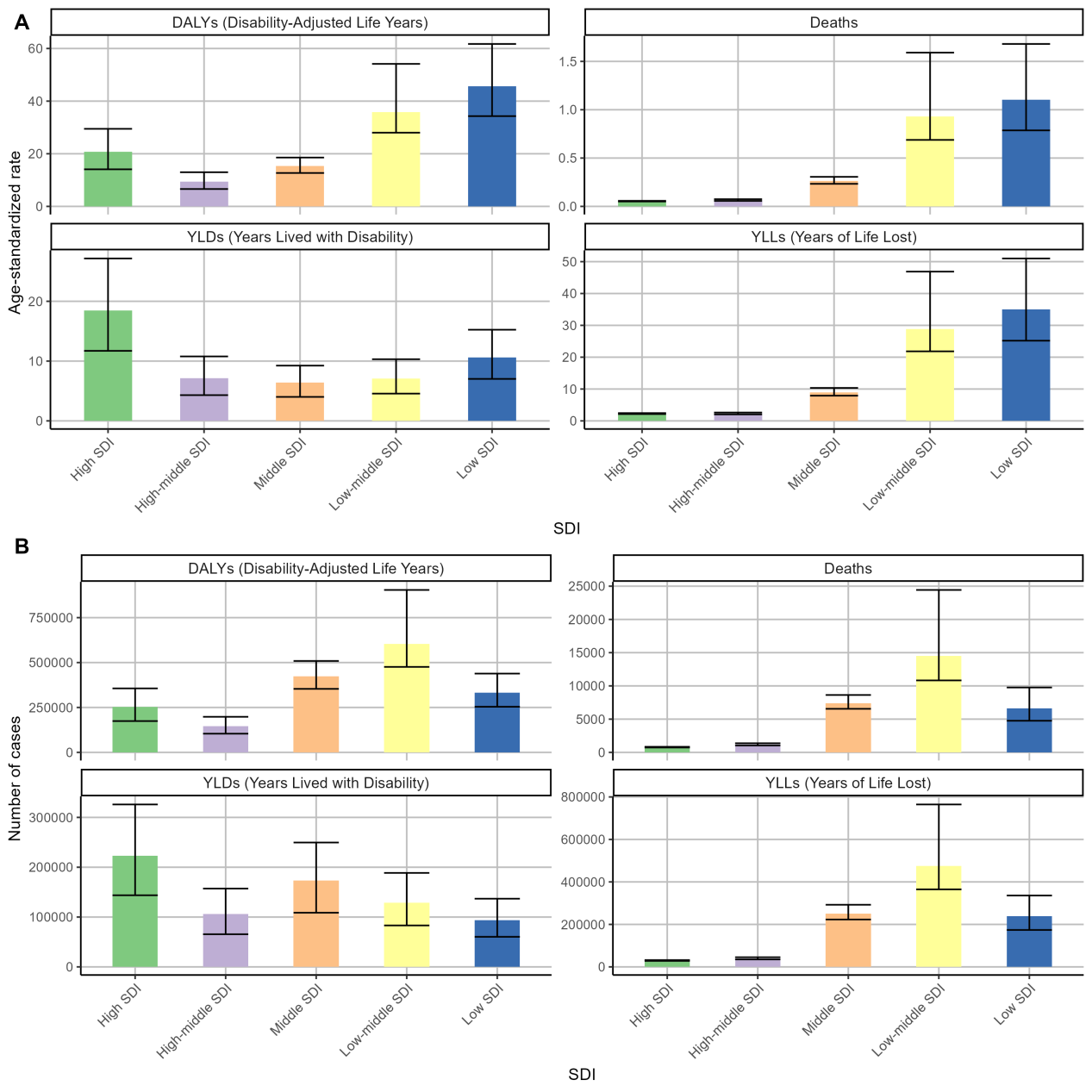
Figure S12.** Numbers and age-standardized rates of asthma attributable to occupational risks-related deaths, DALYs, YLDs, and YLLs for different SDI regions in 2021. Abbreviations: DALYs, disability-adjusted life years; YLDs, years lived with disability; YLLs, years of life lost; SDI, Socio - demographic Index.

**
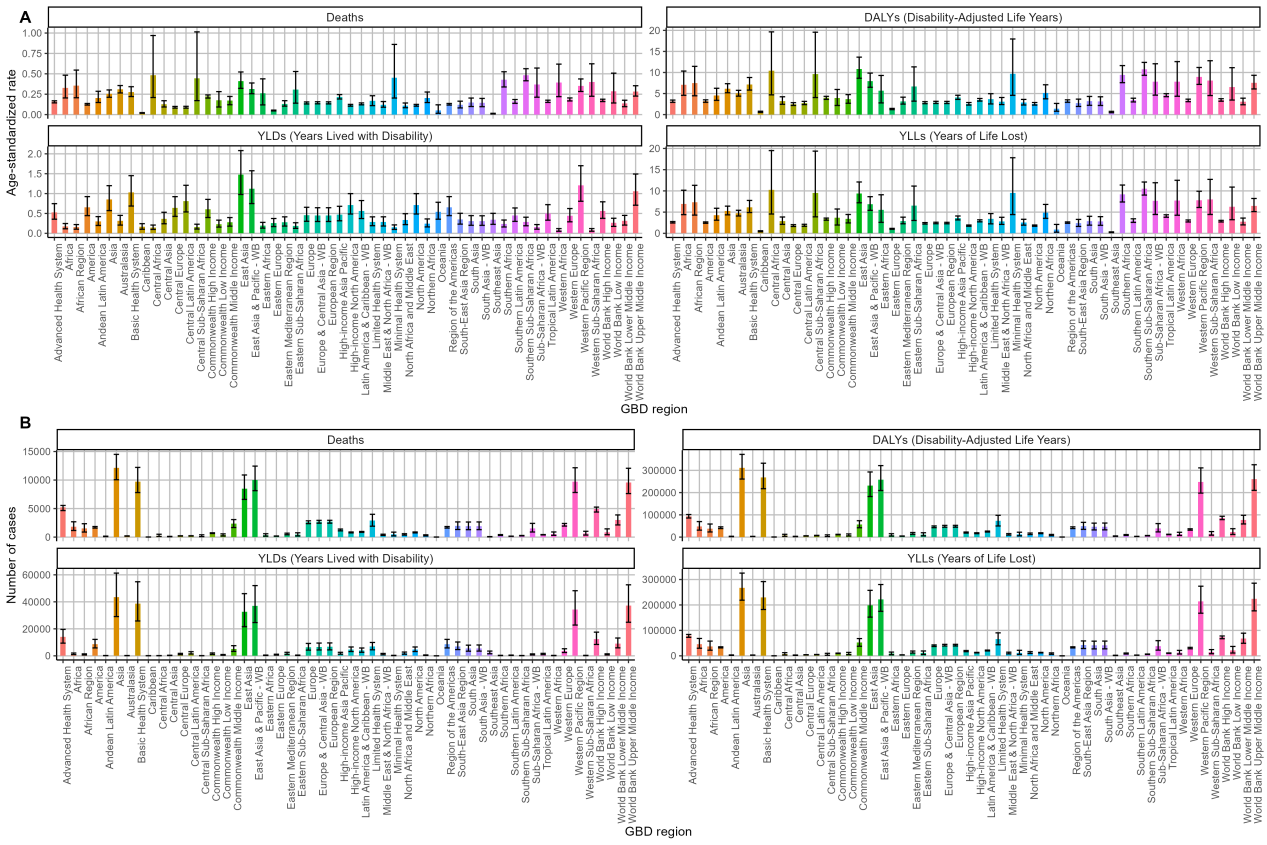
Figure S13.** Numbers and age-standardized rates of pneumoconiosis attributable to occupational risks-related deaths, DALYs, YLDs, and YLLs for different GBD regions in 2021. Abbreviations: DALYs, disability-adjusted life years; YLDs, years lived with disability; YLLs, years of life lost; GBD, Global Burden of Disease.

**
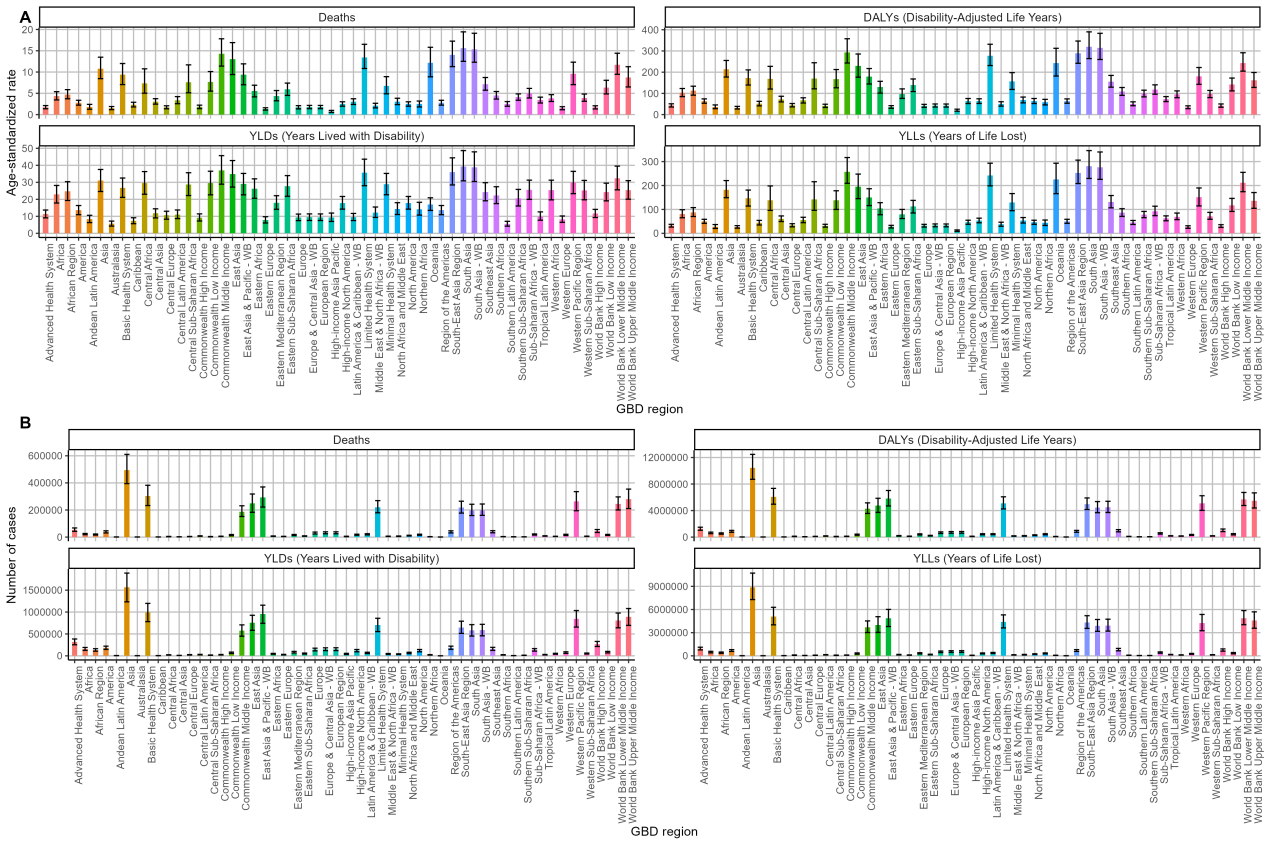
Figure S14.** Numbers and age-standardized rates of chronic obstructive pulmonary disease attributable to occupational risks-related deaths, DALYs, YLDs, and YLLs for different GBD regions in 2021. Abbreviations: DALYs, disability-adjusted life years; YLDs, years lived with disability; YLLs, years of life lost; GBD, Global Burden of Disease.

**
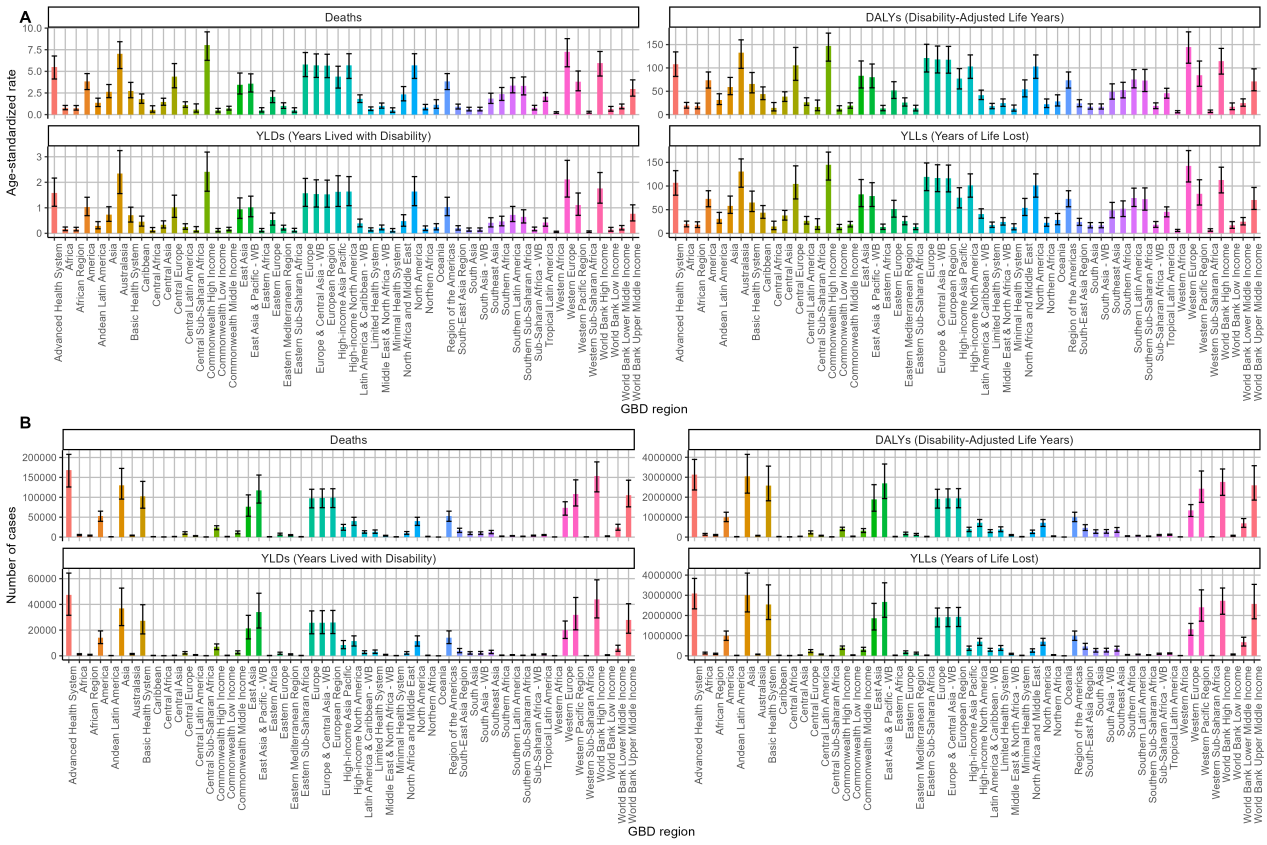
Figure S15.** Numbers and age-standardized rates of tracheal-bronchus-and-lung cancer attributable to occupational risks-related deaths, DALYs, YLDs, and YLLs for different GBD regions in 2021. Abbreviations: DALYs, disability-adjusted life years; YLDs, years lived with disability; YLLs, years of life lost; GBD, Global Burden of Disease.

**
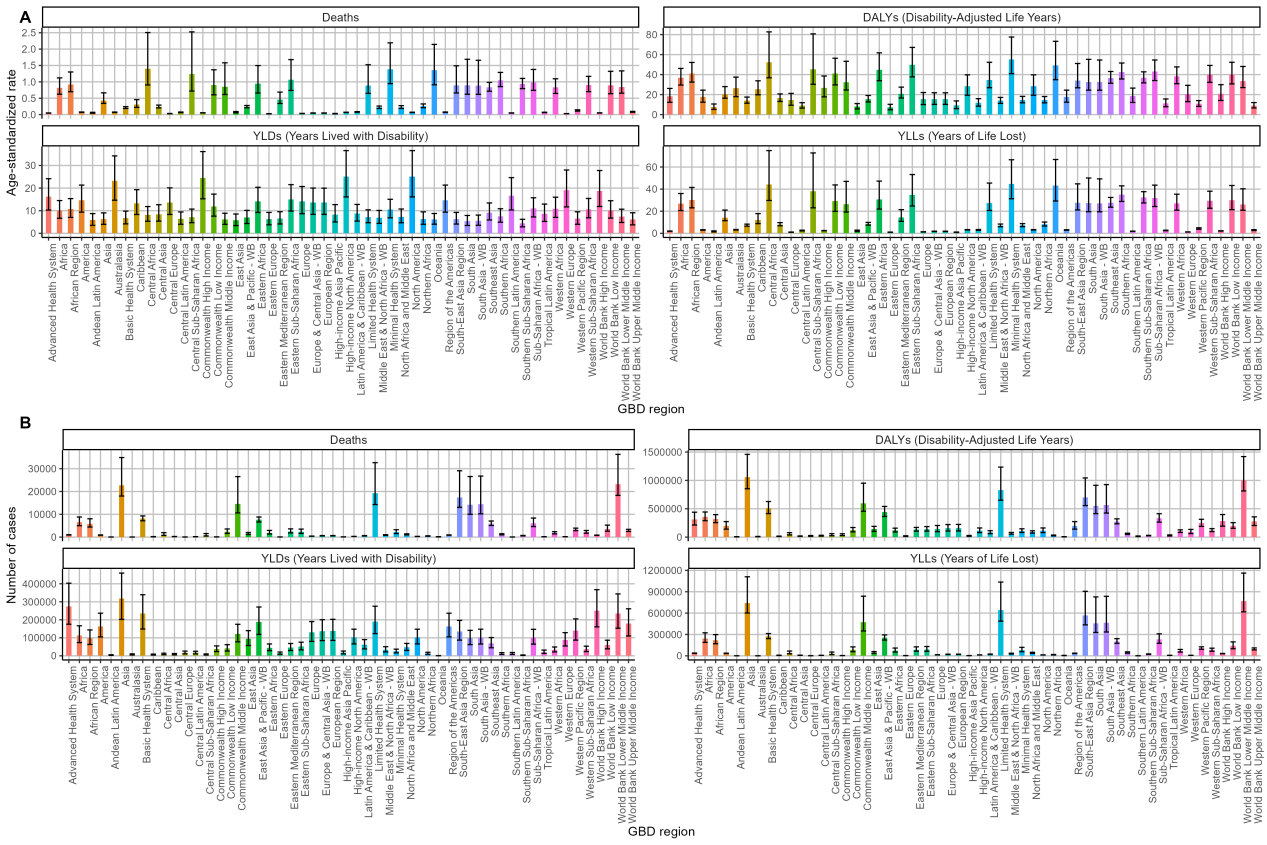
Figure S16.** Numbers and age-standardized rates of asthma attributable to occupational risks-related deaths, DALYs, YLDs, and YLLs for different GBD regions in 2021. Abbreviations: DALYs, disability-adjusted life years; YLDs, years lived with disability; YLLs, years of life lost; GBD, Global Burden of Disease.

**
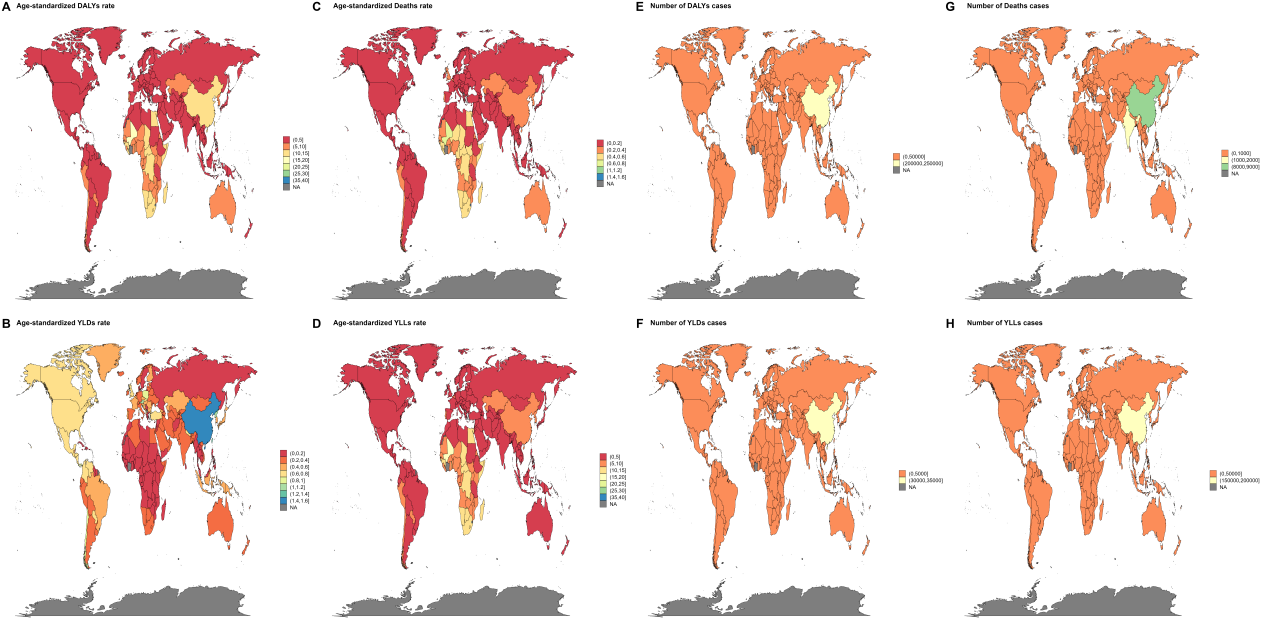
Figure S17.** Numbers and age-standardized rates of pneumoconiosis attributable to occupational risks-related deaths, DALYs, YLDs, and YLLs across countries and territories in 2021. Abbreviations: DALYs, disability-adjusted life years; YLDs, years lived with disability; YLLs, years of life lost.

**
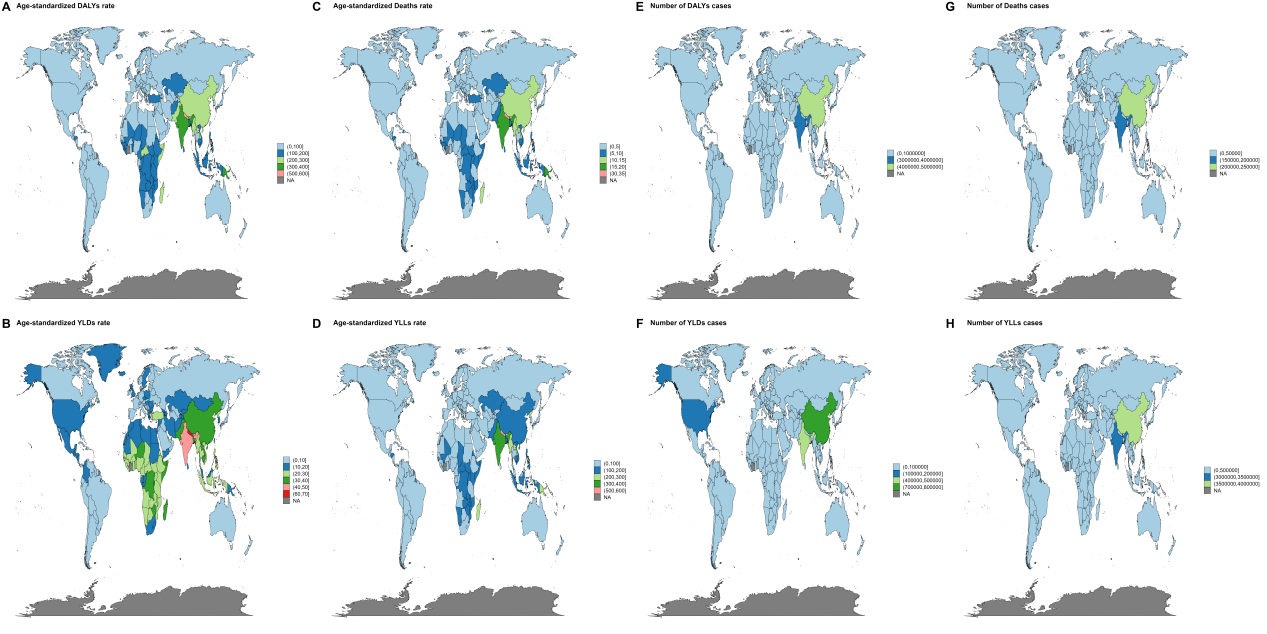
Figure S18.** Numbers and age-standardized rates of chronic obstructive pulmonary disease attributable to occupational risks-related deaths, DALYs, YLDs, and YLLs across countries and territories in 2021. Abbreviations: DALYs, disability-adjusted life years; YLDs, years lived with disability; YLLs, years of life lost.

**
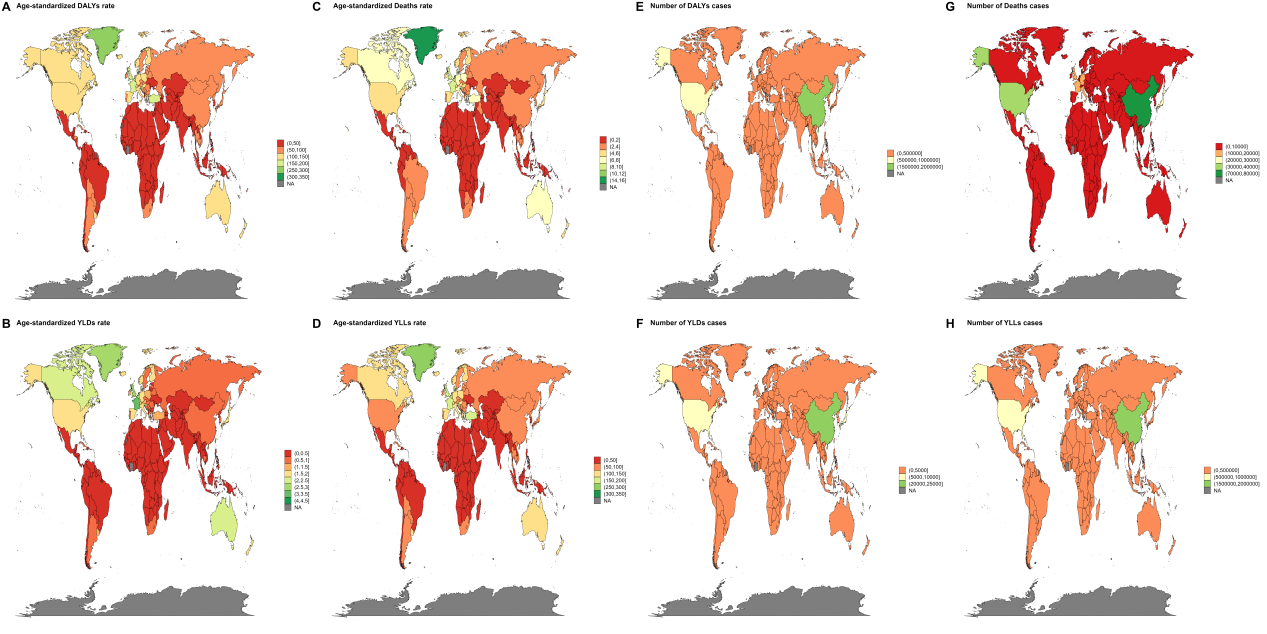
Figure S19.** Numbers and age-standardized rates of tracheal-bronchus-and-lung cancer attributable to occupational risks-related deaths, DALYs, YLDs, and YLLs across countries and territories in 2021. Abbreviations: DALYs, disability-adjusted life years; YLDs, years lived with disability; YLLs, years of life lost.

**
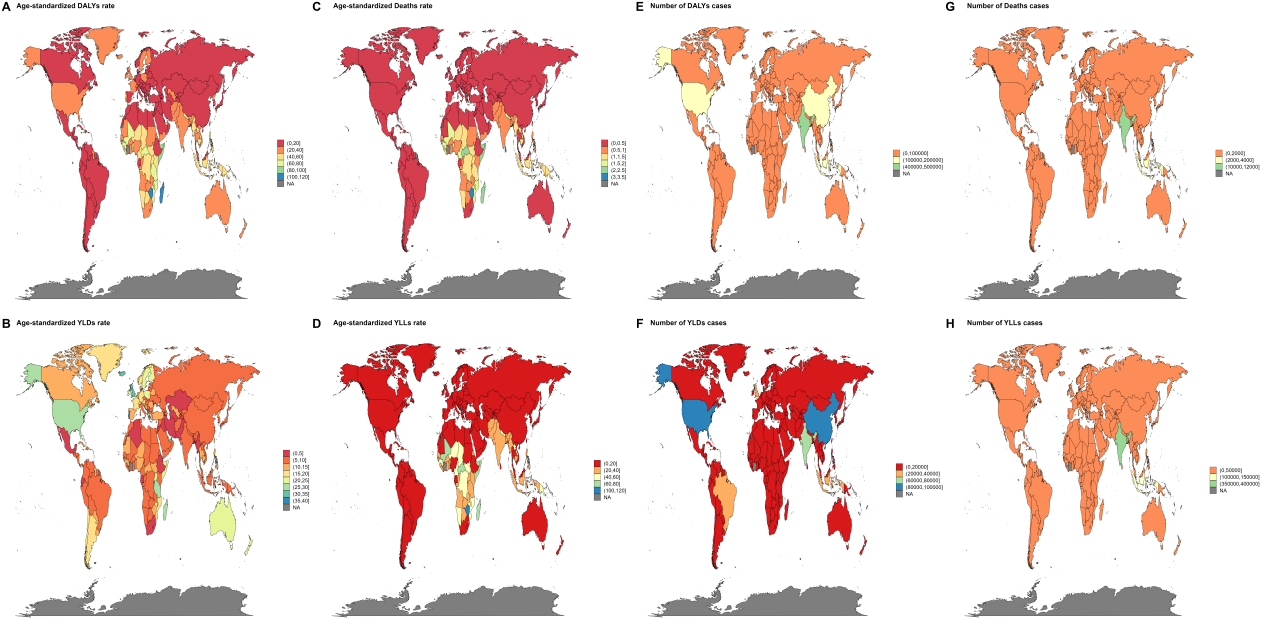
Figure S20.** Numbers and age-standardized rates of asthma attributable to occupational risks-related deaths, DALYs, YLDs, and YLLs across countries and territories in 2021. Abbreviations: DALYs, disability-adjusted life years; YLDs, years lived with disability; YLLs, years of life lost.

**
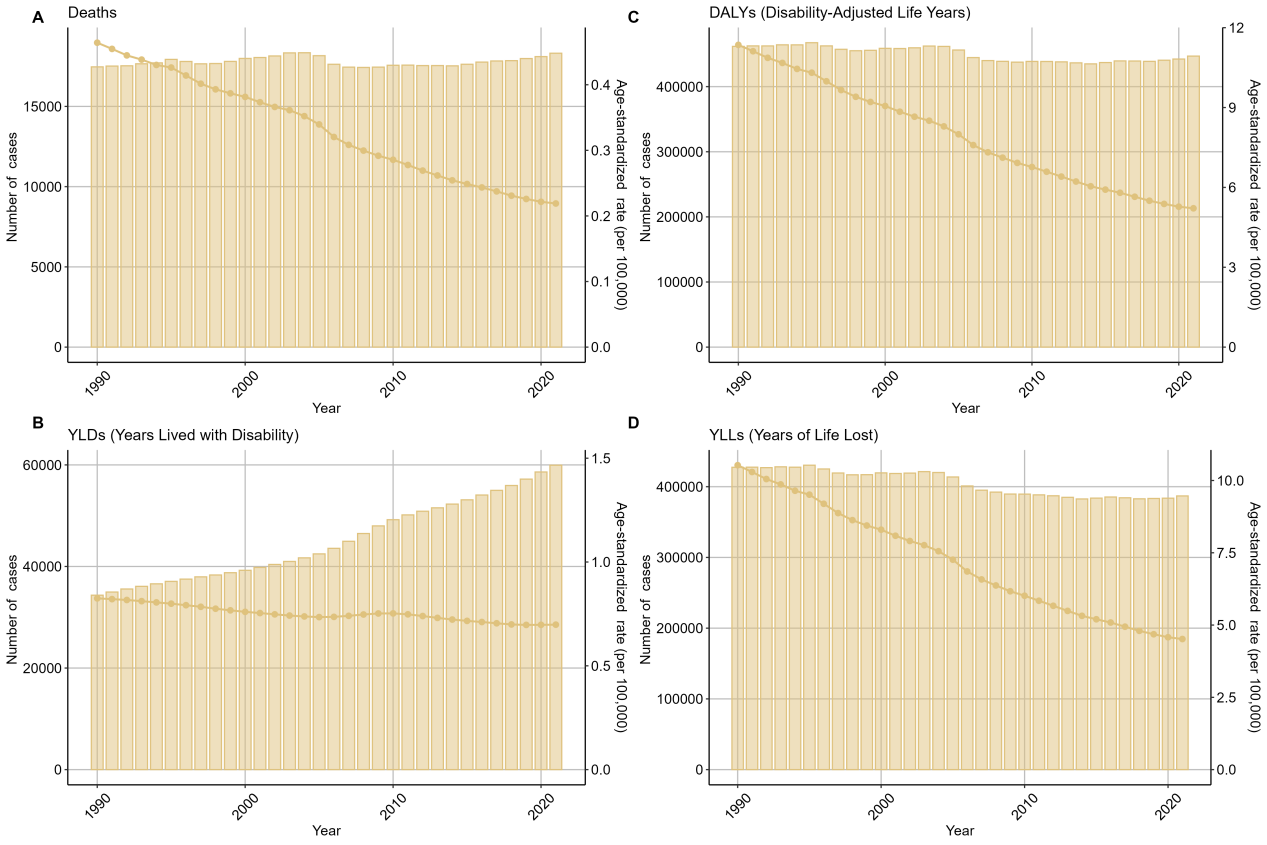
Figure S21.** Trends in the numbers and age-standardized rates of pneumoconiosis attributable to occupational risks globally from 1990 to 2021.

**
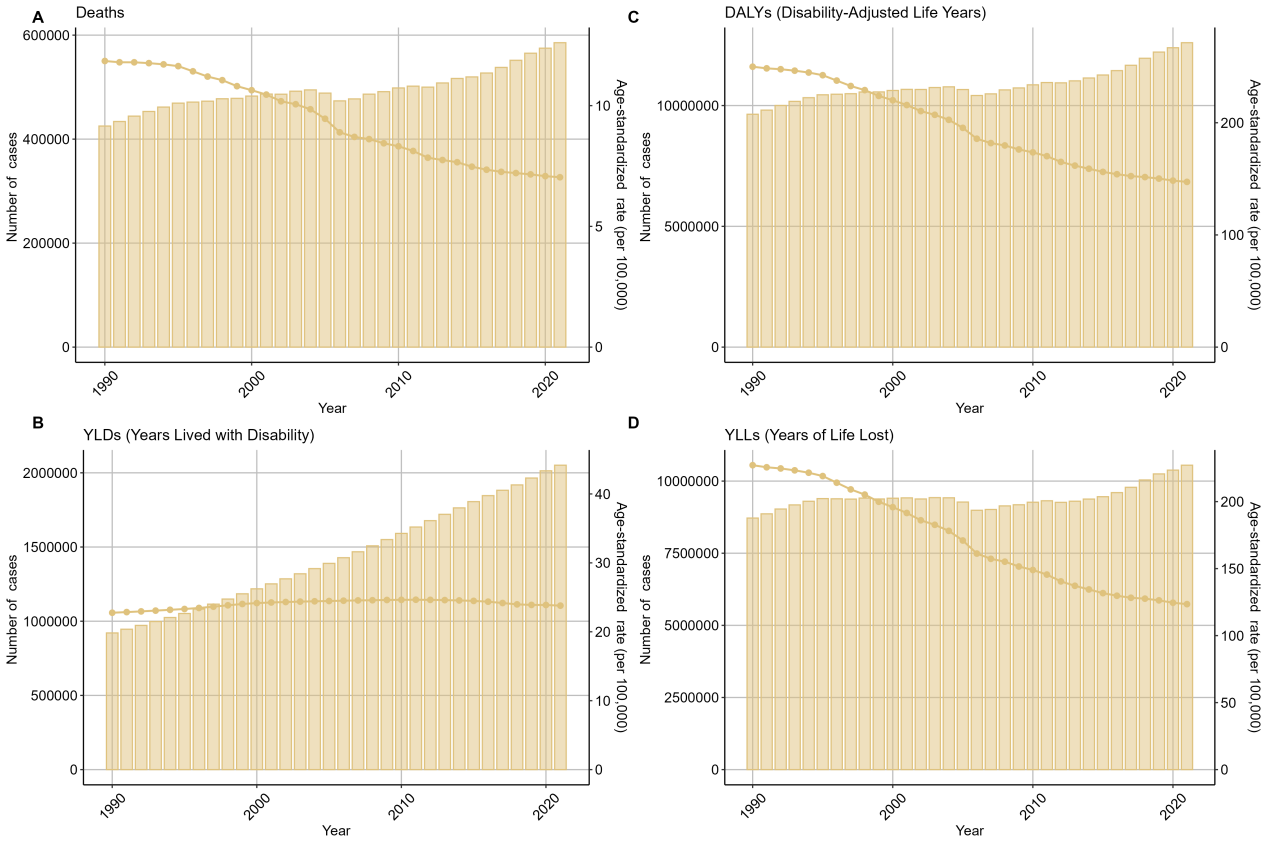
Figure S22.** Trends in the numbers and age-standardized rates of chronic obstructive pulmonary disease attributable to occupational risks globally from 1990 to 2021.

**
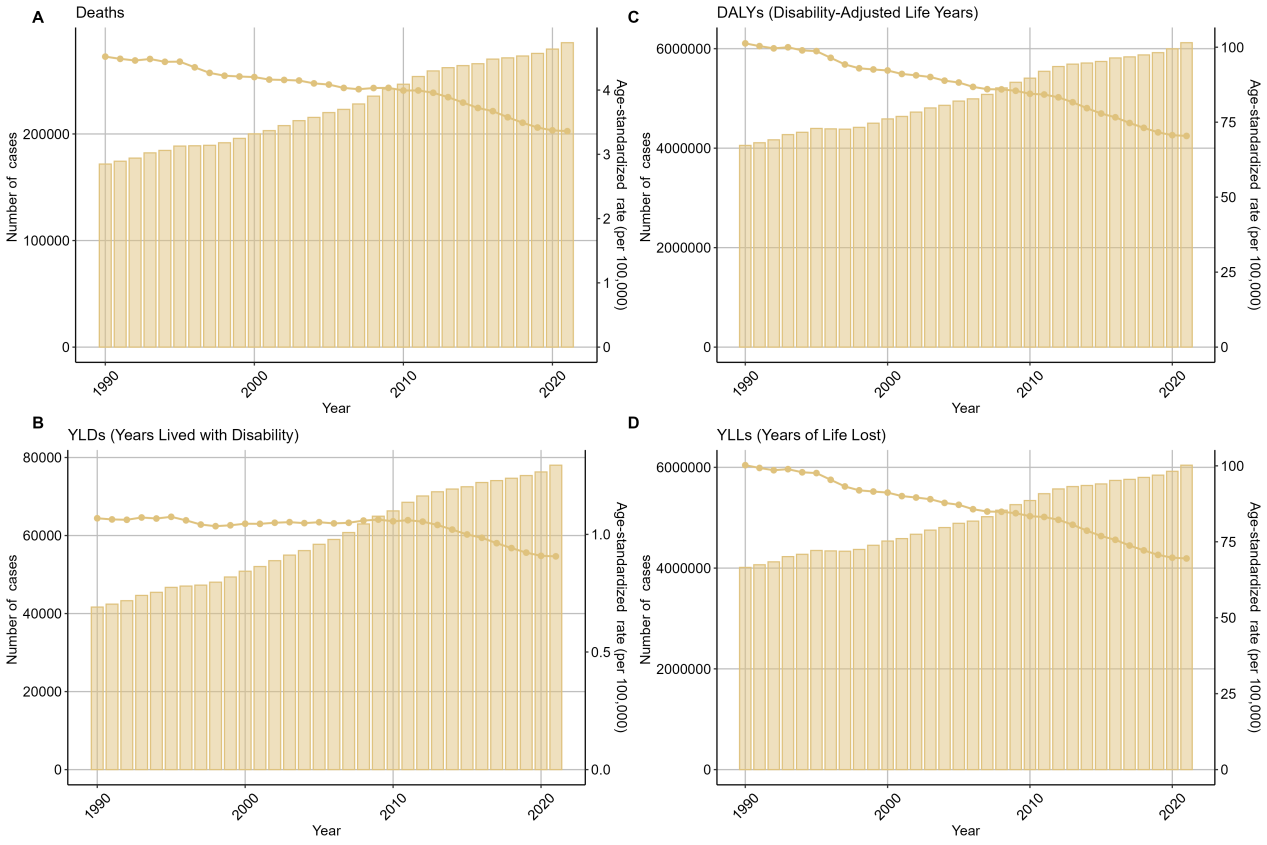
Figure S23.** Trends in the numbers and age-standardized rates of tracheal-bronchus-and-lung cancer attributable to occupational risks globally from 1990 to 2021.

**
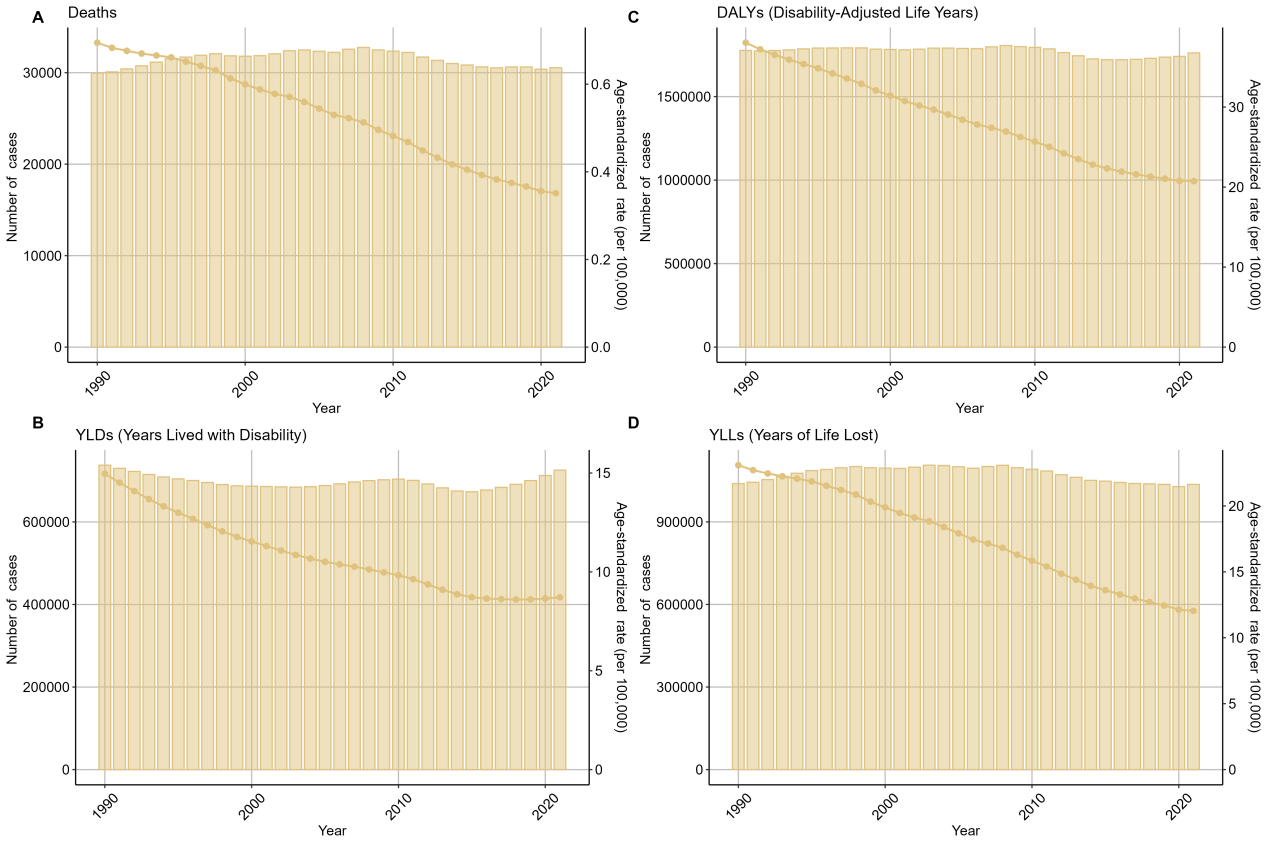
Figure S24.** Trends in the numbers and age-standardized rates of asthma attributable to occupational risks globally from 1990 to 2021.

**
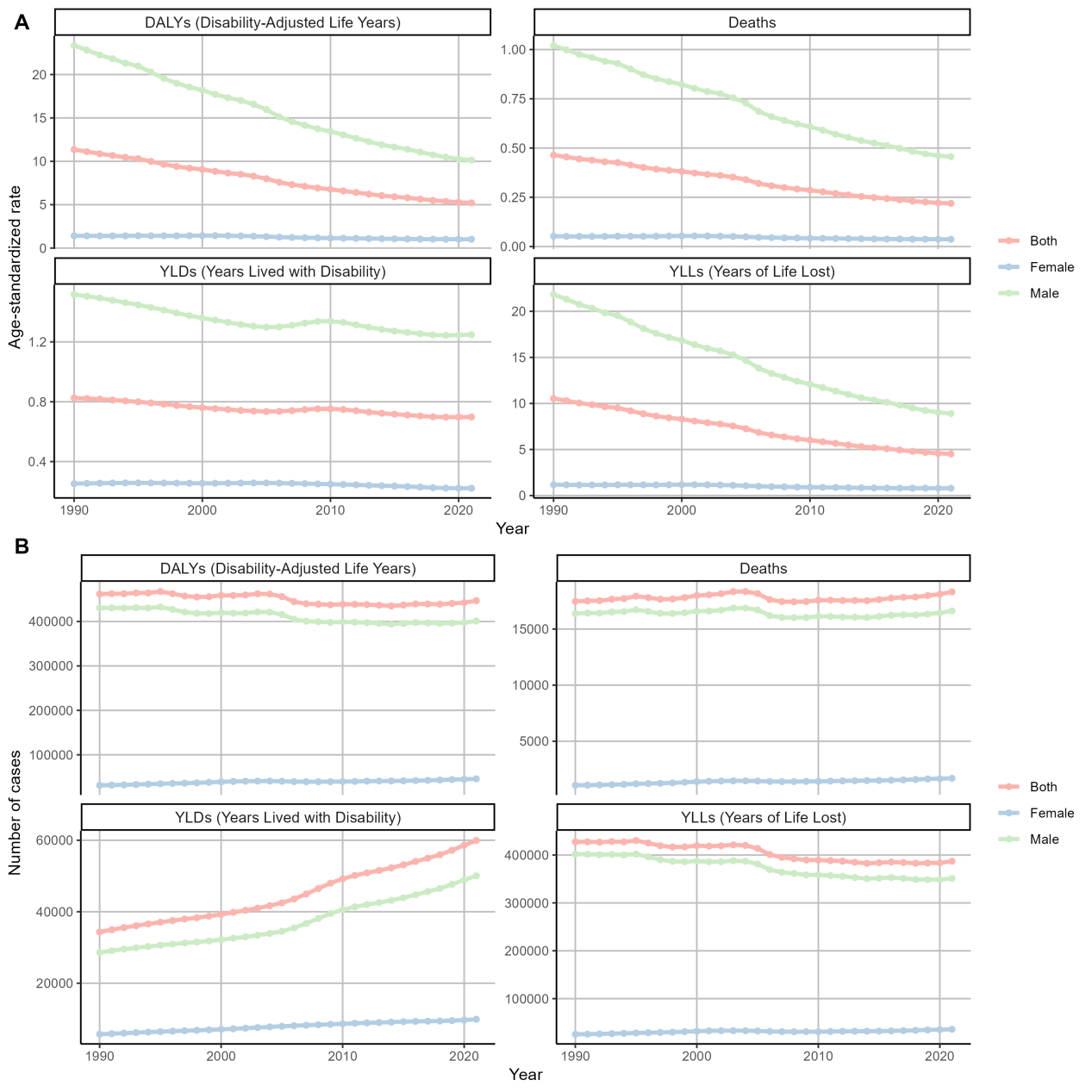
Figure S25.** Trends in the numbers and age-standardized rates of pneumoconiosis attributable to occupational risks-related deaths, DALYs, YLDs, and YLLs globally by sexes from 1990 to 2021. Abbreviations: DALYs, disability-adjusted life years; YLDs, years lived with disability; YLLs, years of life lost.

**
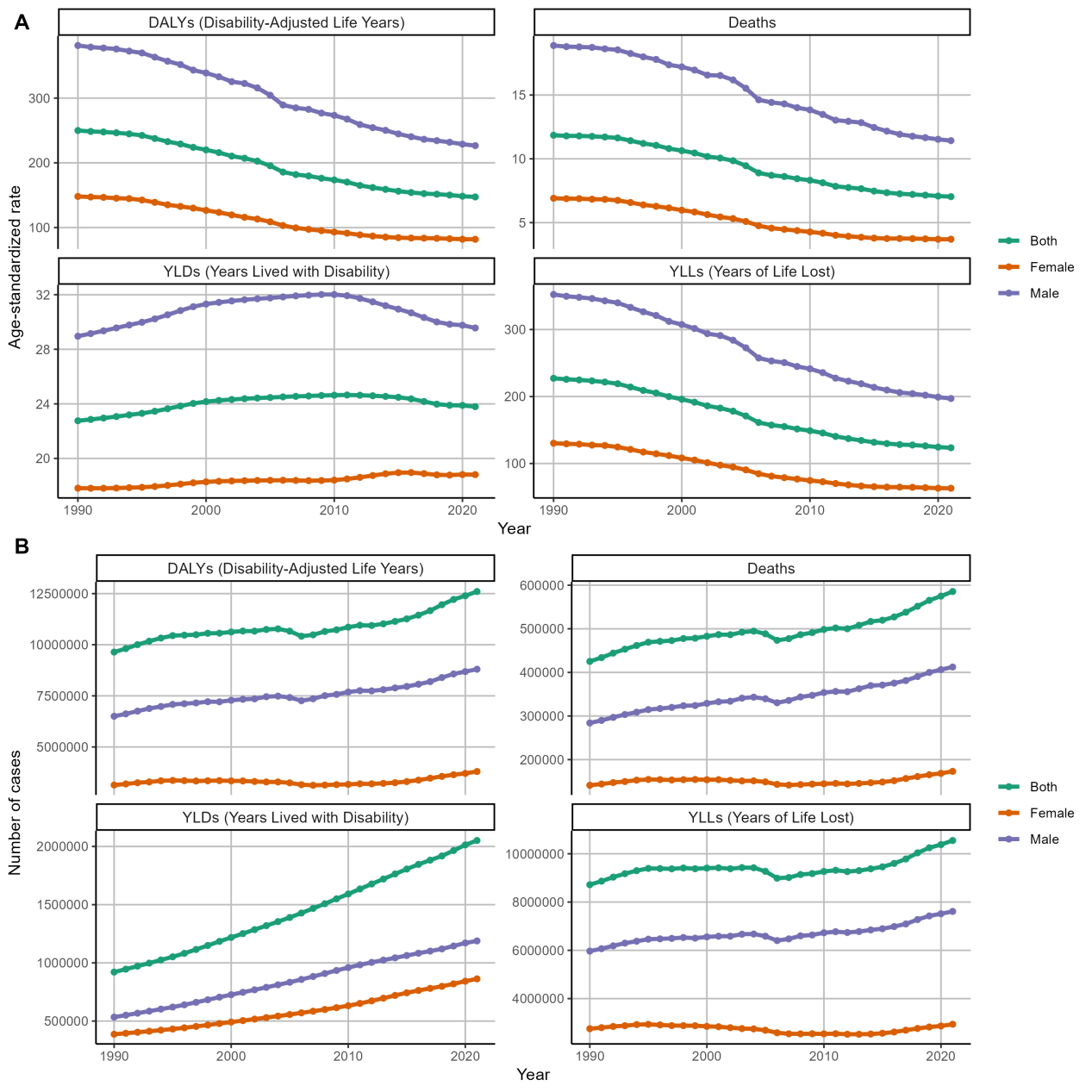
Figure S26.** Trends in the numbers and age-standardized rates of chronic obstructive pulmonary disease attributable to occupational risks-related deaths, DALYs, YLDs, and YLLs globally by sexes from 1990 to 2021. Abbreviations: DALYs, disability-adjusted life years; YLDs, years lived with disability; YLLs, years of life lost.

**
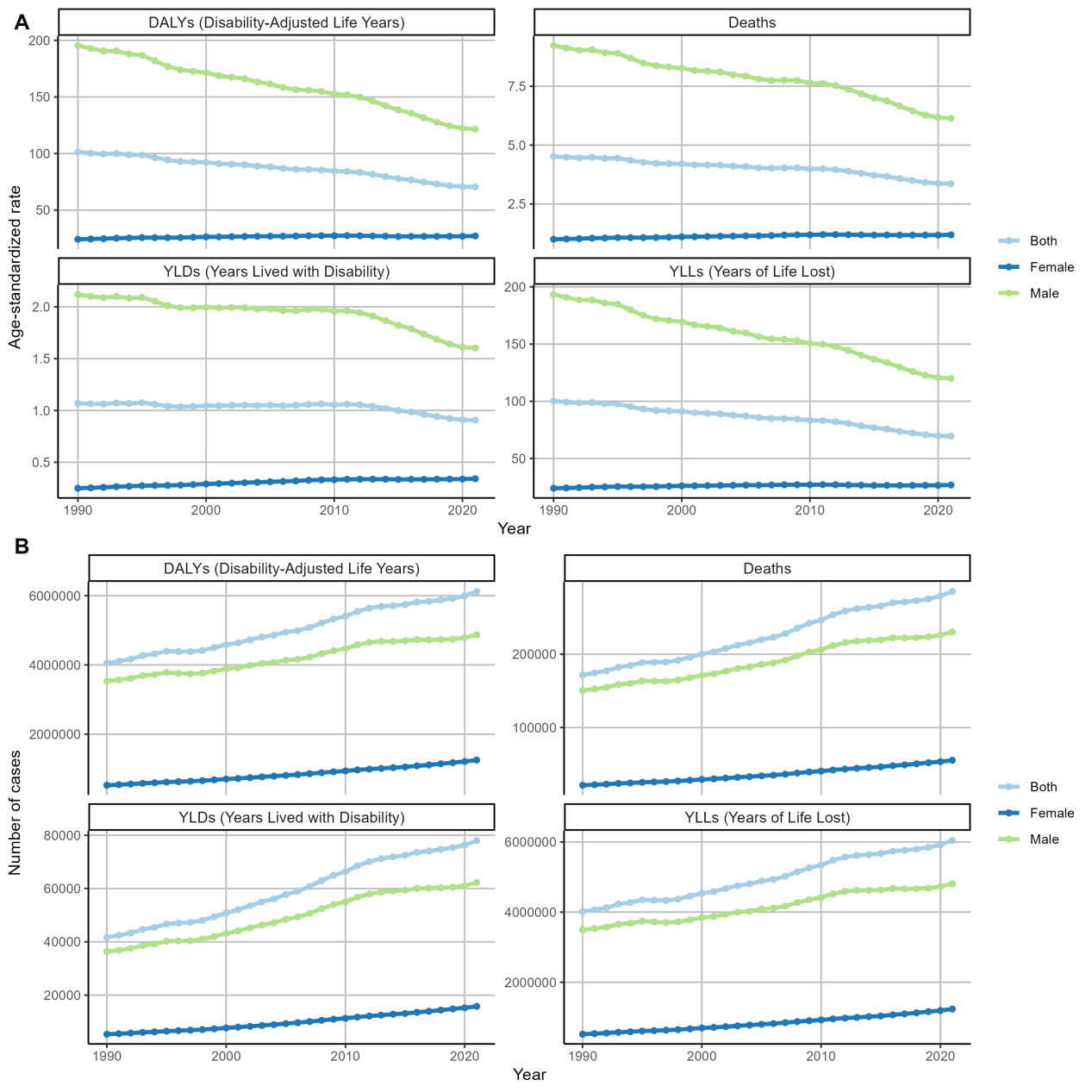
Figure S27.** Trends in the numbers and age-standardized rates of tracheal-bronchus-and-lung cancer attributable to occupational risks-related deaths, DALYs, YLDs, and YLLs globally by sexes from 1990 to 2021. Abbreviations: DALYs, disability-adjusted life years; YLDs, years lived with disability; YLLs, years of life lost.

**
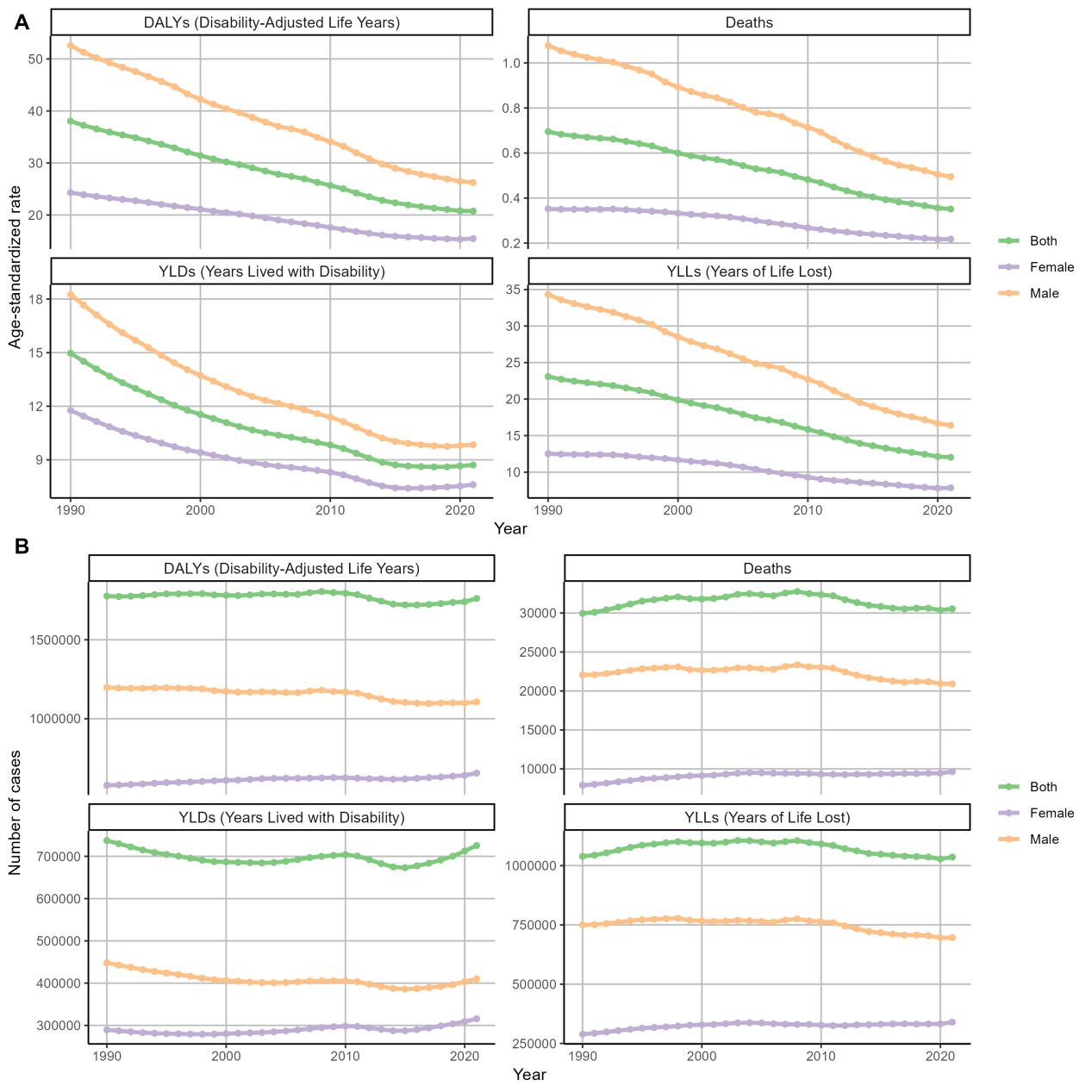
Figure S28.** Trends in the numbers and age-standardized rates of asthma attributable to occupational risks-related deaths, DALYs, YLDs, and YLLs globally by sexes from 1990 to 2021. Abbreviations: DALYs, disability-adjusted life years; YLDs, years lived with disability; YLLs, years of life lost.

**
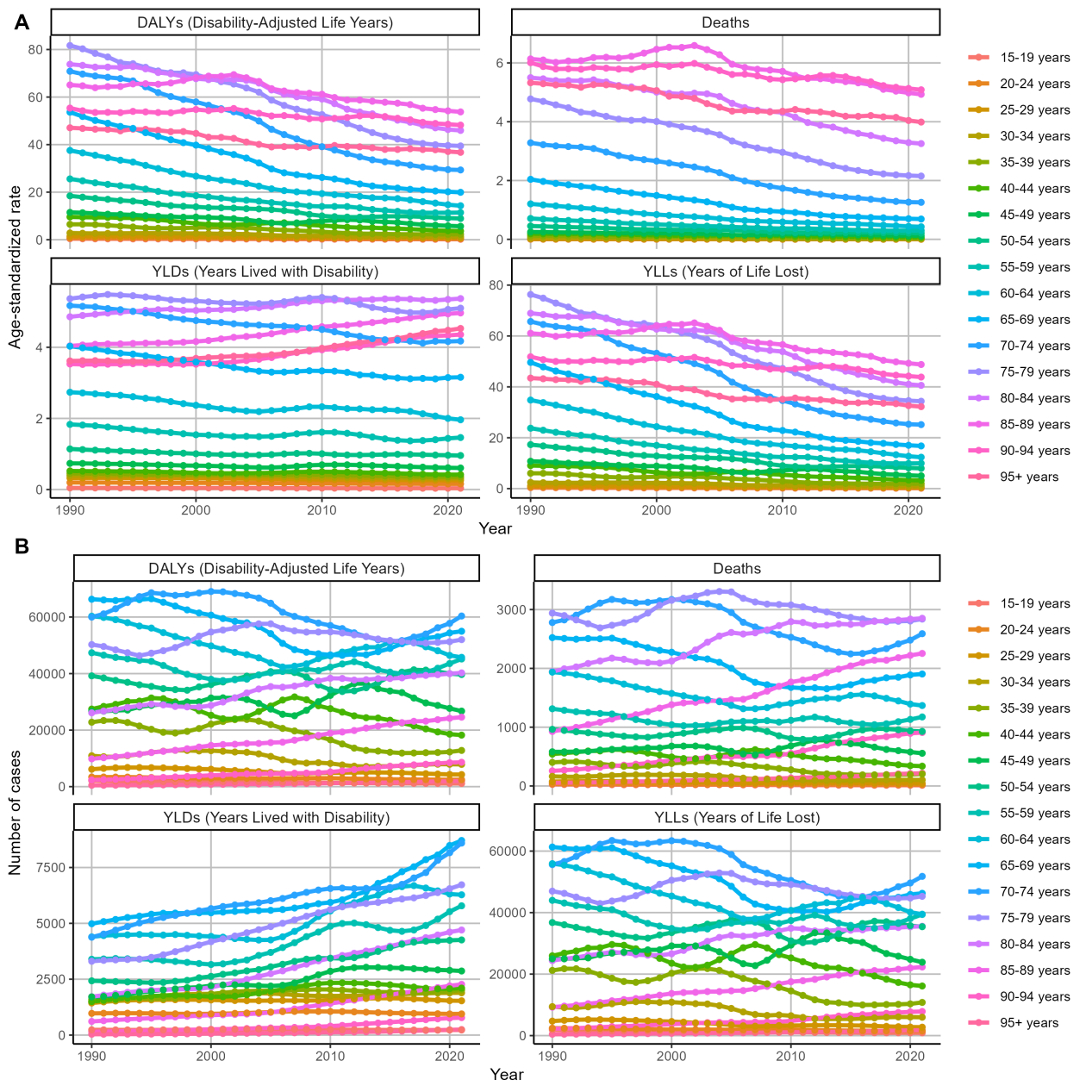
Figure S29.** Trends in the numbers and age-standardized rates of pneumoconiosis attributable to occupational risks-related deaths, DALYs, YLDs, and YLLs globally by age groups from 1990 to 2021. Abbreviations: DALYs, disability-adjusted life years; YLDs, years lived with disability; YLLs, years of life lost.

**
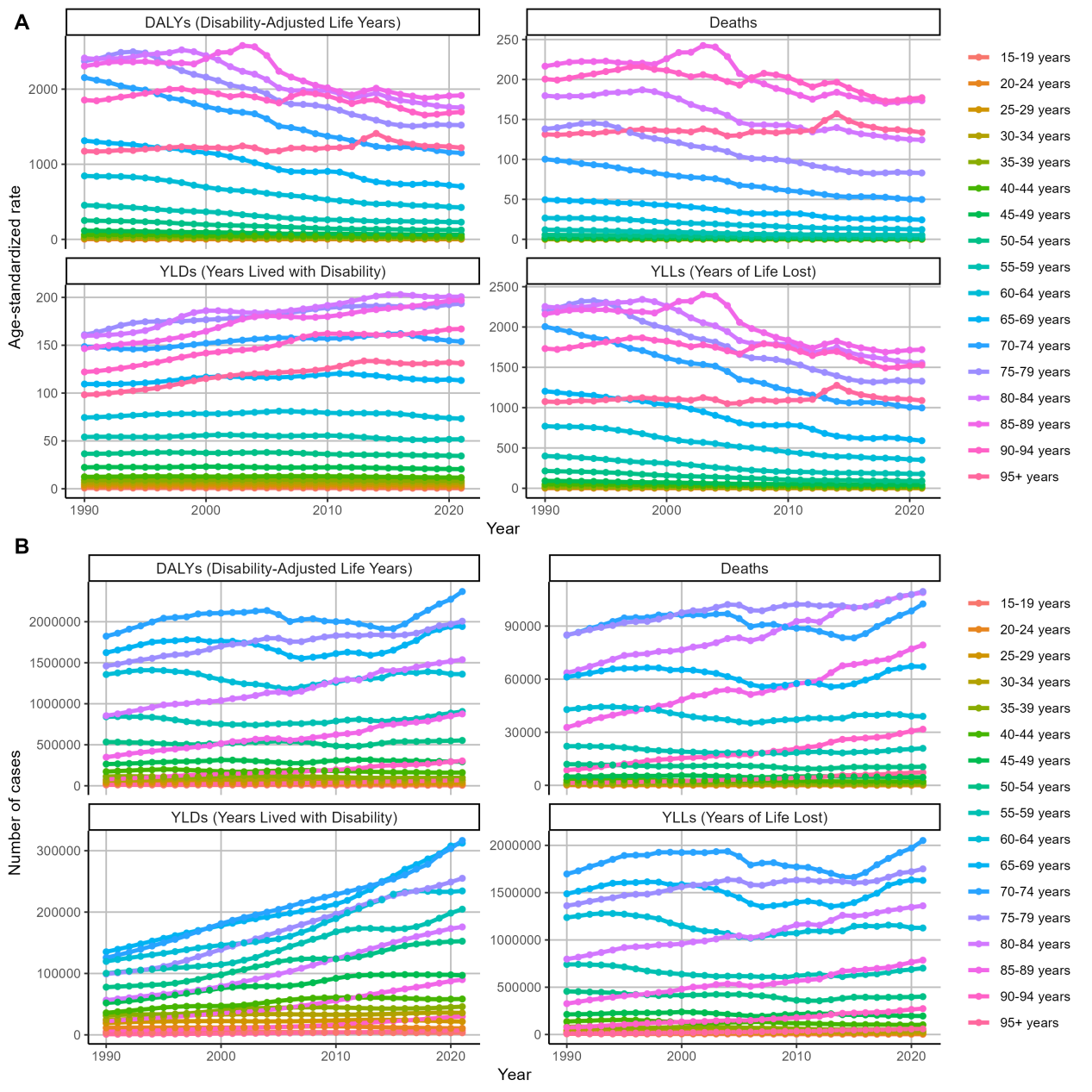
Figure S30.** Trends in the numbers and age-standardized rates of chronic obstructive pulmonary disease attributable to occupational risks-related deaths, DALYs, YLDs, and YLLs globally by age groups from 1990 to 2021. Abbreviations: DALYs, disability-adjusted life years; YLDs, years lived with disability; YLLs, years of life lost.

**
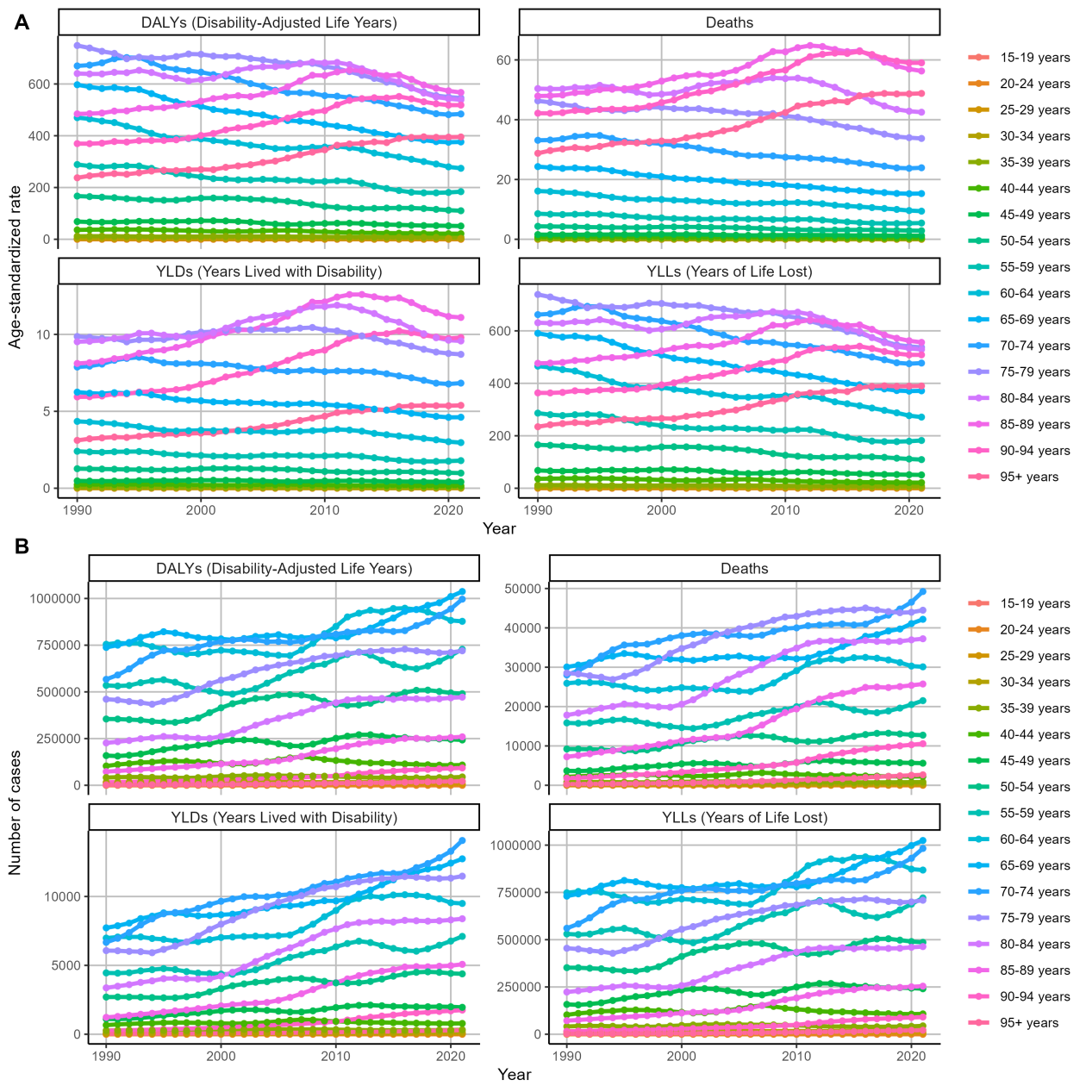
Figure S31.** Trends in the numbers and age-standardized rates of tracheal-bronchus-and-lung cancer attributable to occupational risks-related deaths, DALYs, YLDs, and YLLs globally by age groups from 1990 to 2021. Abbreviations: DALYs, disability-adjusted life years; YLDs, years lived with disability; YLLs, years of life lost.

**
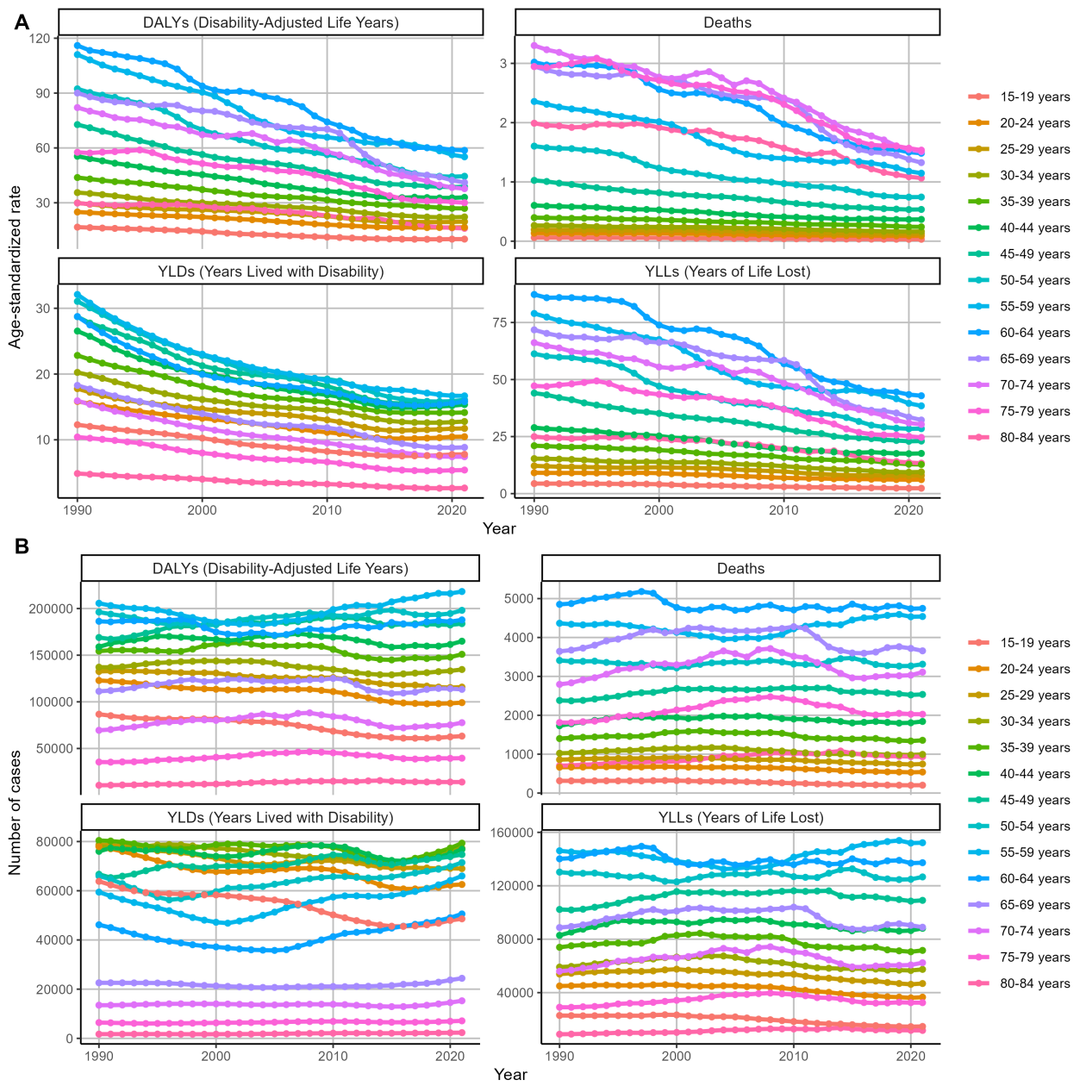
Figure S32.** Trends in the numbers and age-standardized rates of asthma attributable to occupational risks-related deaths, DALYs, YLDs, and YLLs globally by age groups from 1990 to 2021. Abbreviations: DALYs, disability-adjusted life years; YLDs, years lived with disability; YLLs, years of life lost.

**
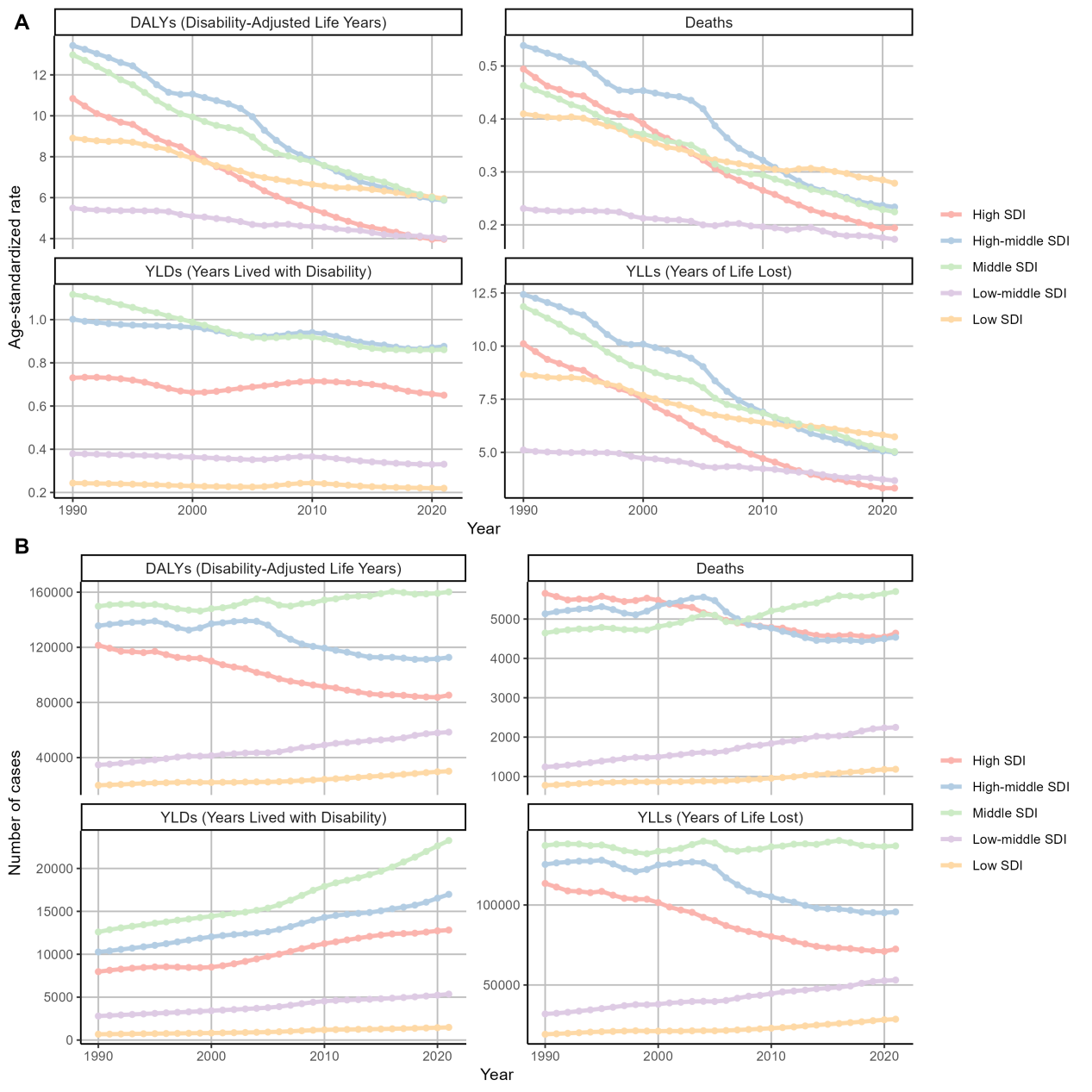
Figure S33.** Trends in the numbers and age-standardized rates of pneumoconiosis attributable to occupational risks-related deaths, DALYs, YLDs, and YLLs globally by SDI regions from 1990 to 2021. Abbreviations: DALYs, disability-adjusted life years; YLDs, years lived with disability; YLLs, years of life lost; SDI, Socio - demographic Index.

**
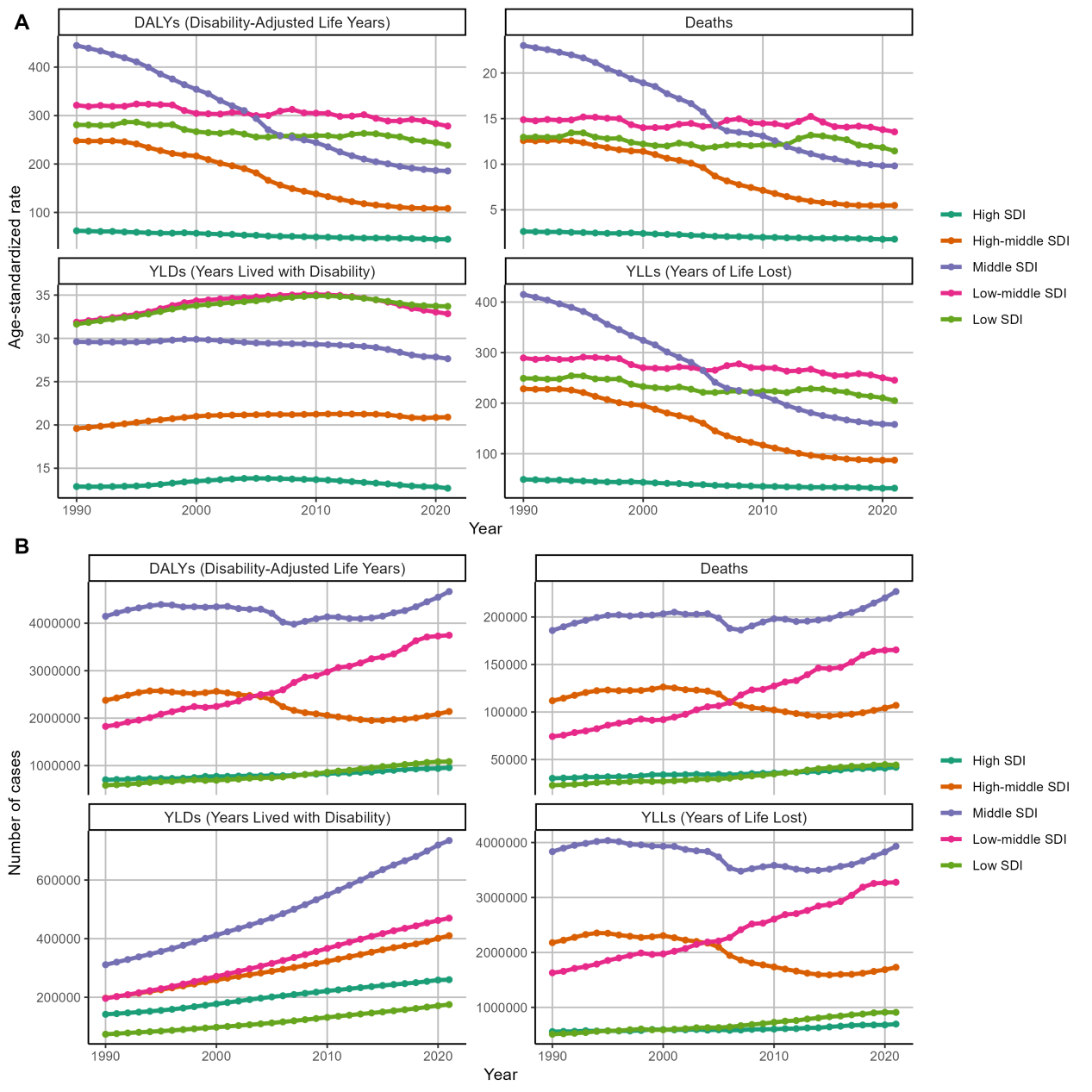
Figure S34.** Trends in the numbers and age-standardized rates of chronic obstructive pulmonary disease attributable to occupational risks-related deaths, DALYs, YLDs, and YLLs globally by SDI regions from 1990 to 2021. Abbreviations: DALYs, disability-adjusted life years; YLDs, years lived with disability; YLLs, years of life lost; SDI, Socio - demographic Index.

**
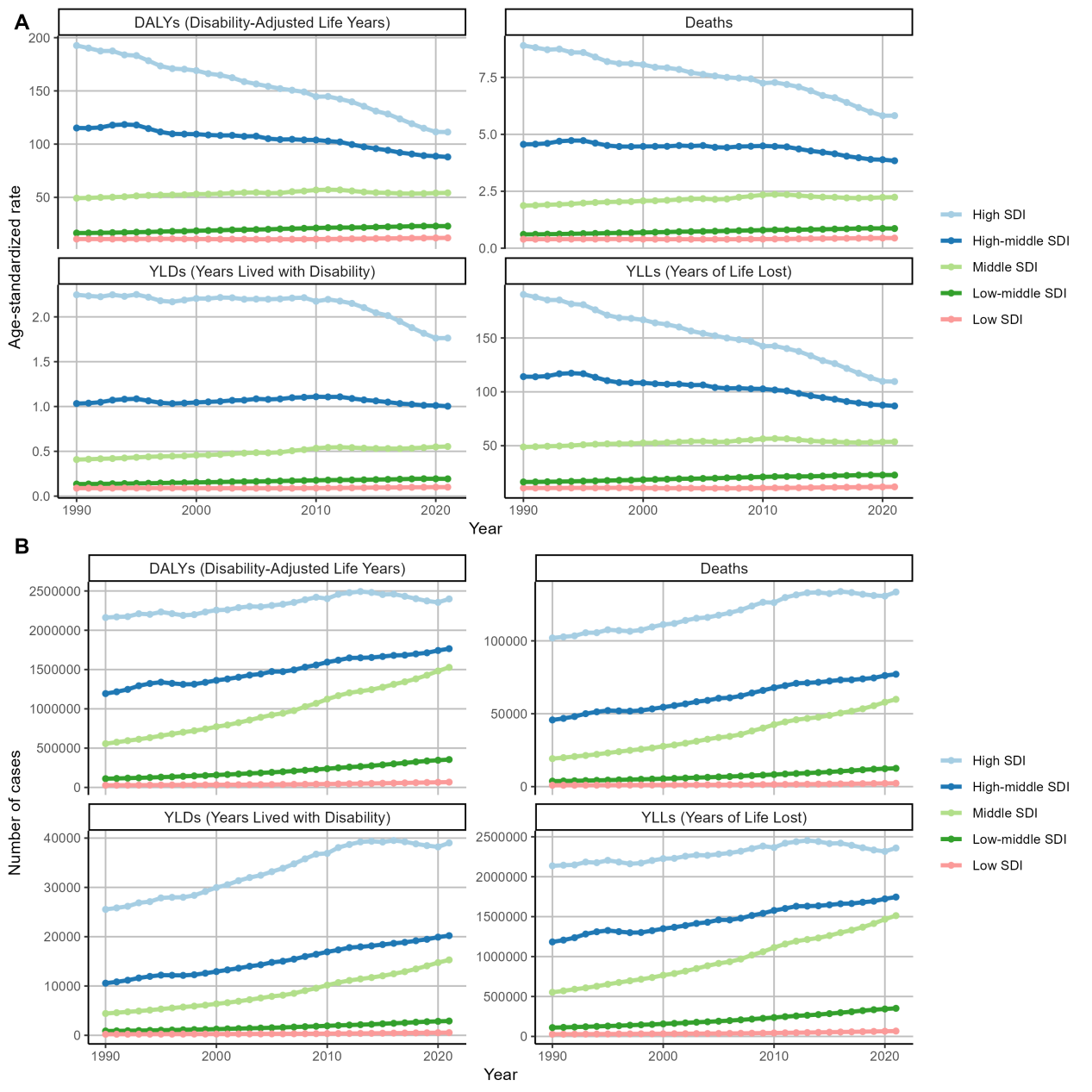
Figure S35.** Trends in the numbers and age-standardized rates of tracheal-bronchus-and-lung cancer attributable to occupational risks-related deaths, DALYs, YLDs, and YLLs globally by SDI regions from 1990 to 2021. Abbreviations: DALYs, disability-adjusted life years; YLDs, years lived with disability; YLLs, years of life lost; SDI, Socio - demographic Index.

**
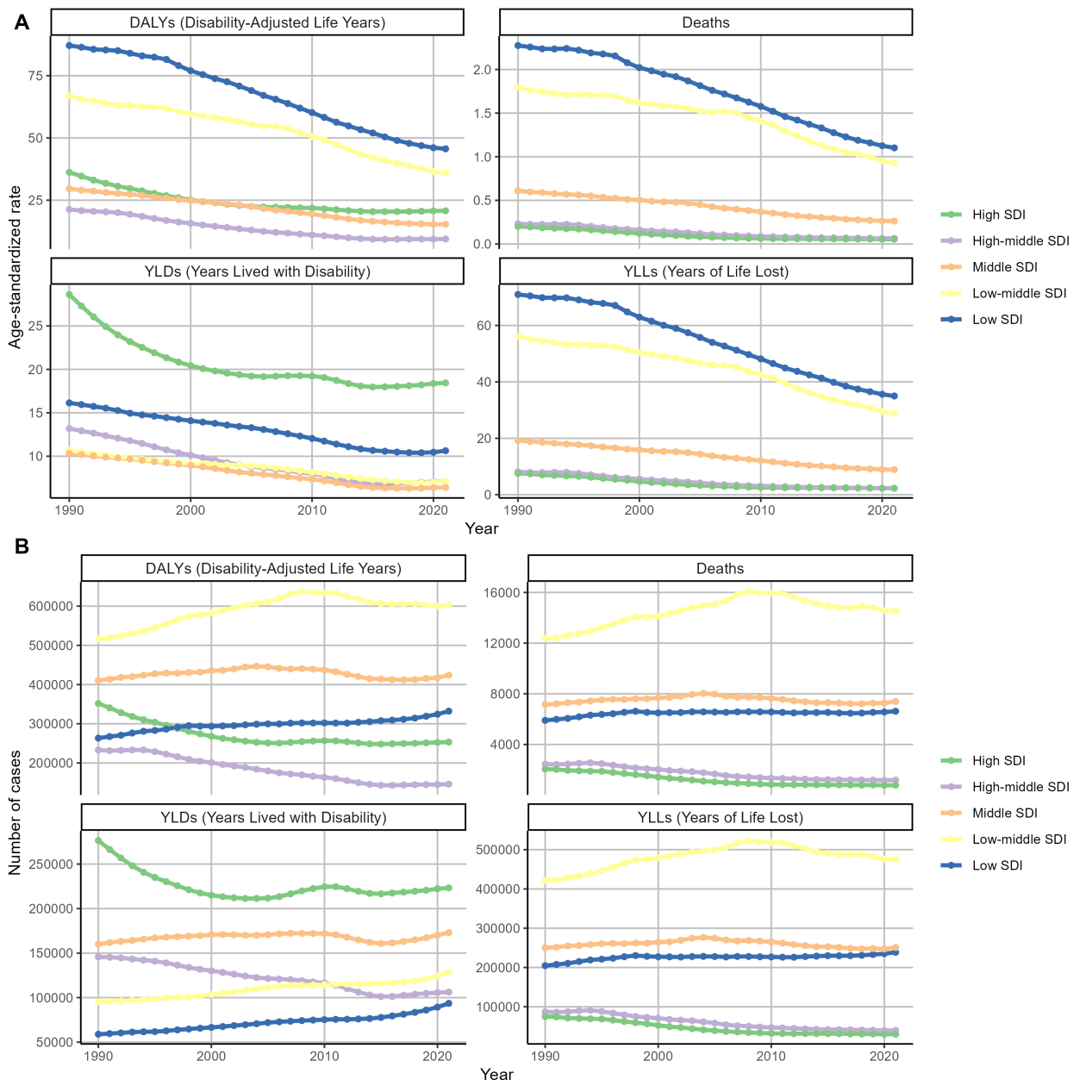
Figure S36.** Trends in the numbers and age-standardized rates of asthma attributable to occupational risks-related deaths, DALYs, YLDs, and YLLs globally by SDI regions from 1990 to 2021. Abbreviations: DALYs, disability-adjusted life years; YLDs, years lived with disability; YLLs, years of life lost; SDI, Socio - demographic Index.

**
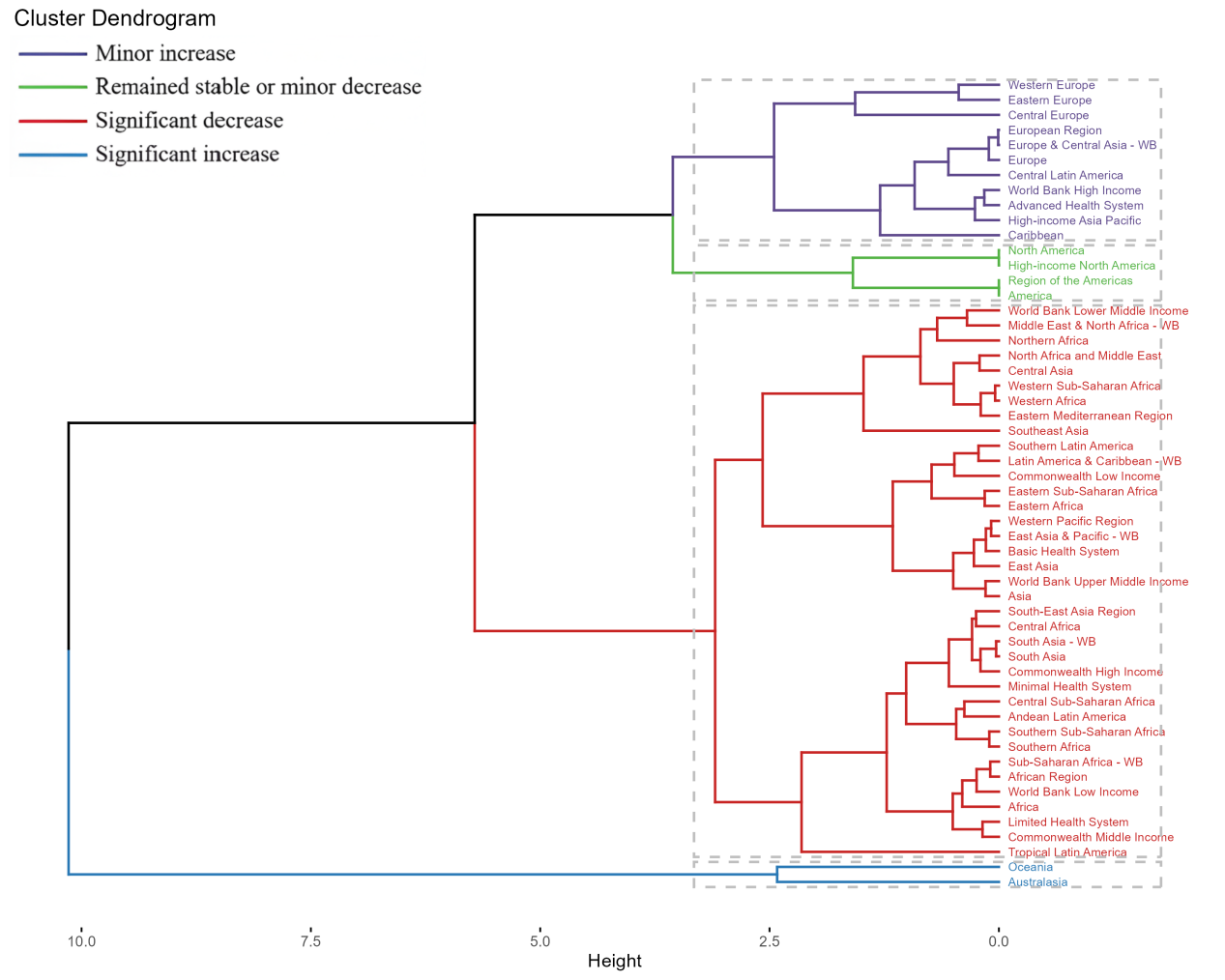
Figure S37.** Results of cluster analysis based on the EAPC values of the age-standardized rates of pneumoconiosis attributable to occupational risks from 1990 to 2021. Abbreviations: EAPC, estimated annual percentage change.

**
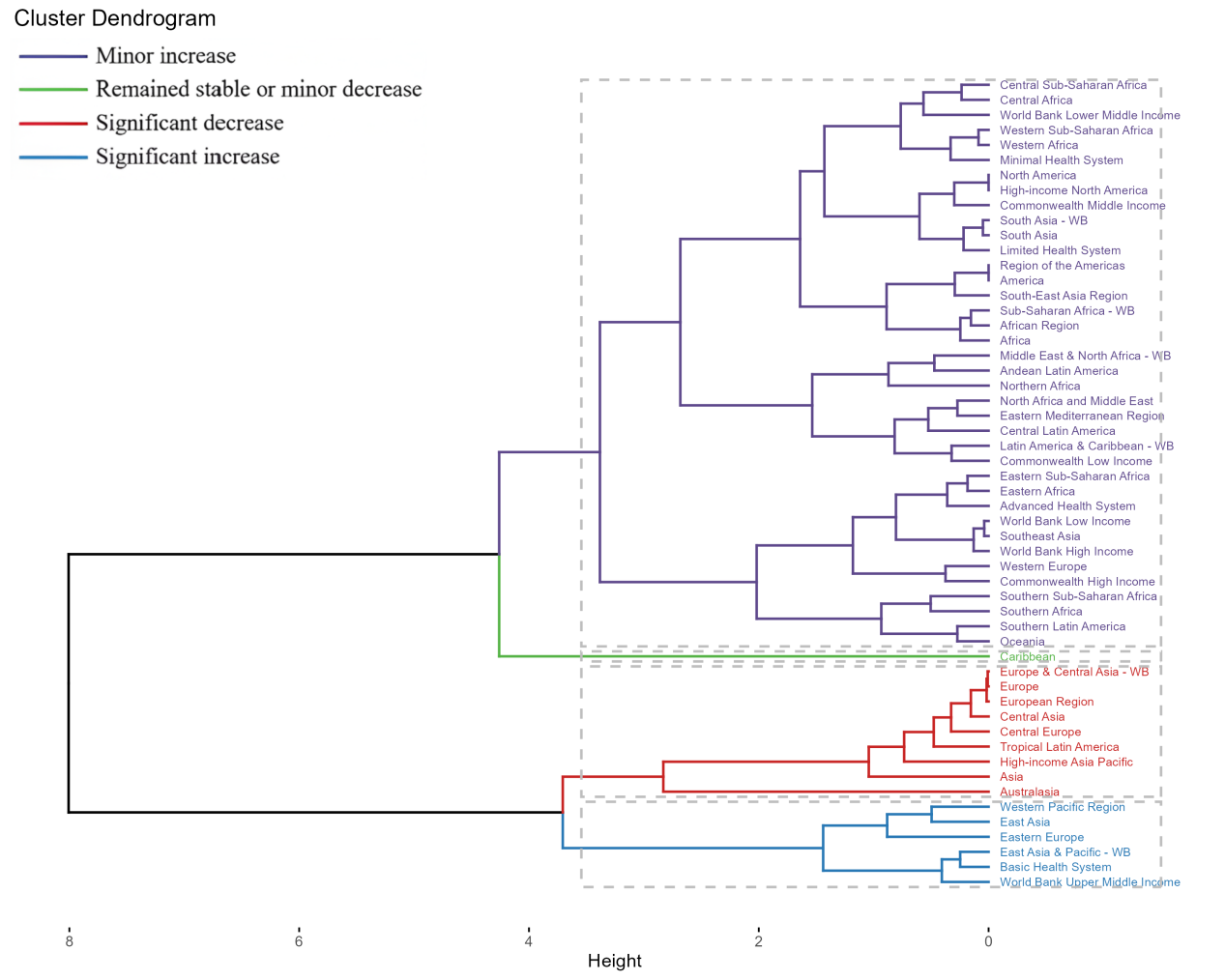
Figure S38.** Results of cluster analysis based on the EAPC values of the age-standardized rates of chronic obstructive pulmonary disease attributable to occupational risks from 1990 to 2021. Abbreviations: EAPC, estimated annual percentage change.

**
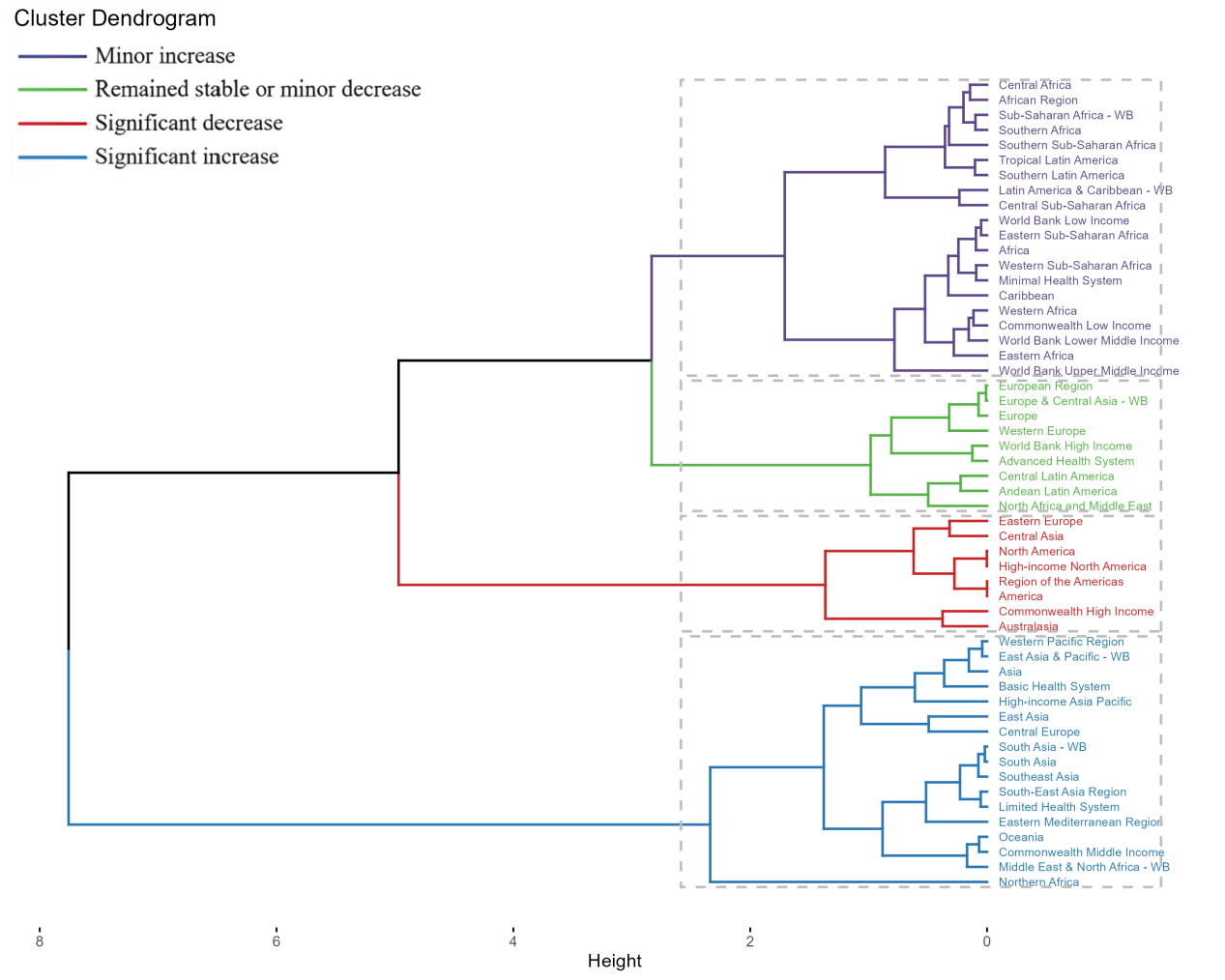
Figure S39.** Results of cluster analysis based on the EAPC values of the age-standardized rates of tracheal-bronchus-and-lung cancer attributable to occupational risks from 1990 to 2021. Abbreviations: EAPC, estimated annual percentage change.

**
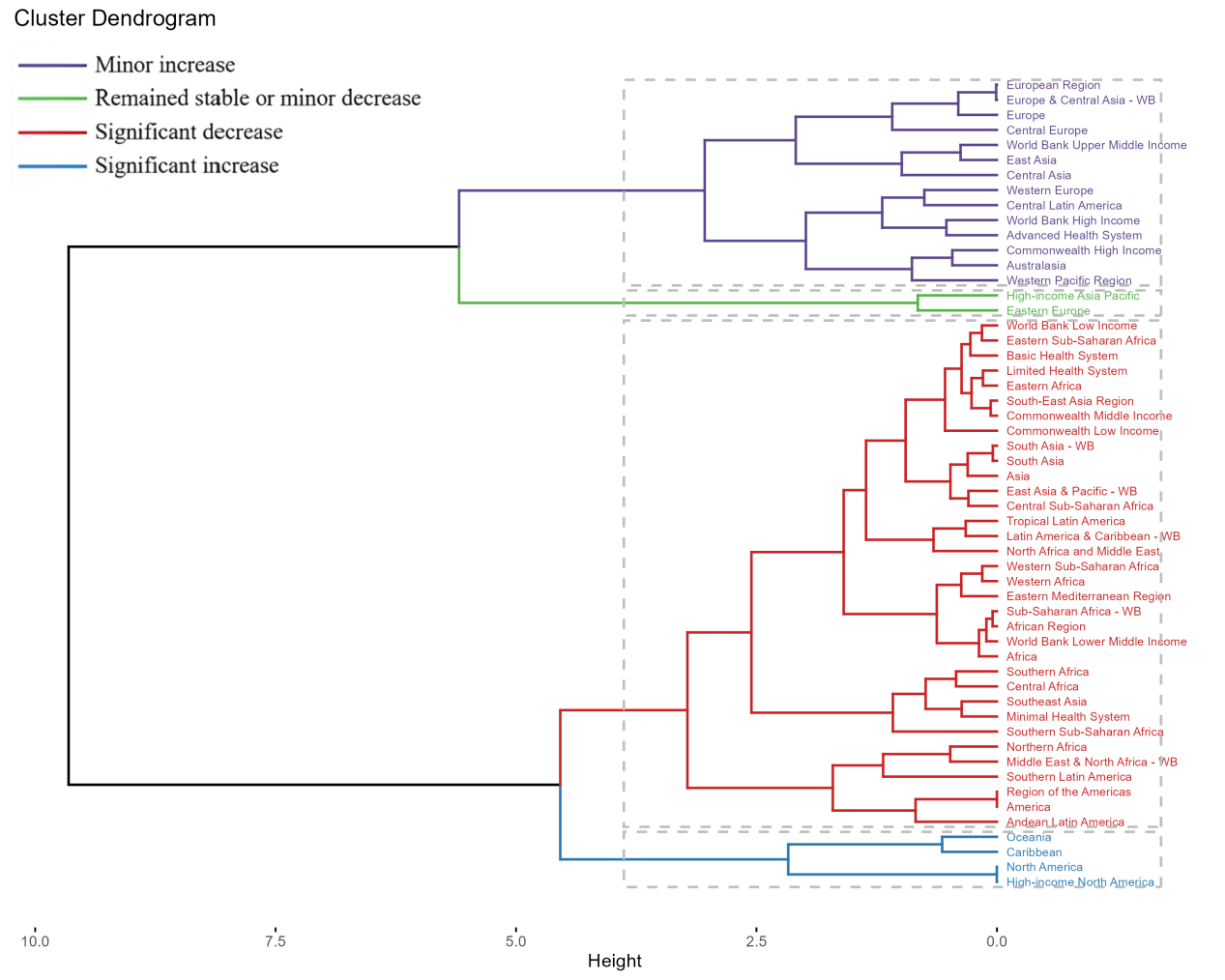
Figure S40.** Results of cluster analysis based on the EAPC values of the age-standardized rates of asthma attributable to occupational risks from 1990 to 2021. Abbreviations: EAPC, estimated annual percentage change.

**
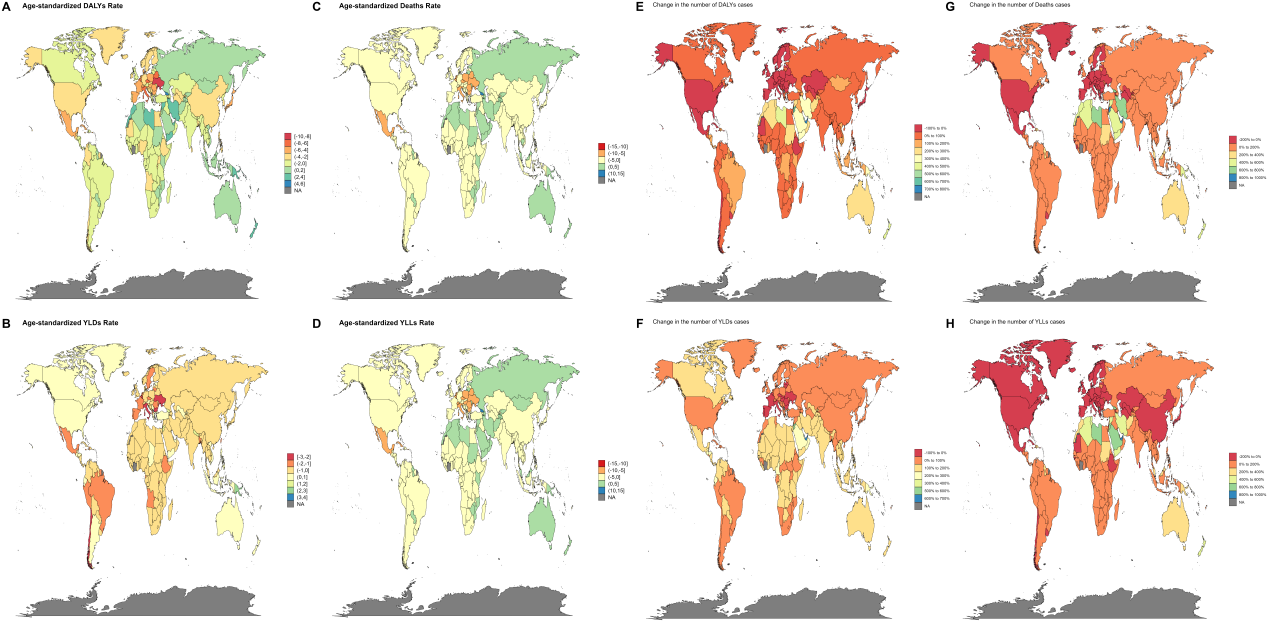
Figure S41.** Trends in the numbers and age-standardized rates of pneumoconiosis attributable to occupational risks-related deaths, DALYs, YLDs, and YLLs globally across countries and territories from 1990 to 2021. Abbreviations: DALYs, disability-adjusted life years; YLDs, years lived with disability; YLLs, years of life lost.

**
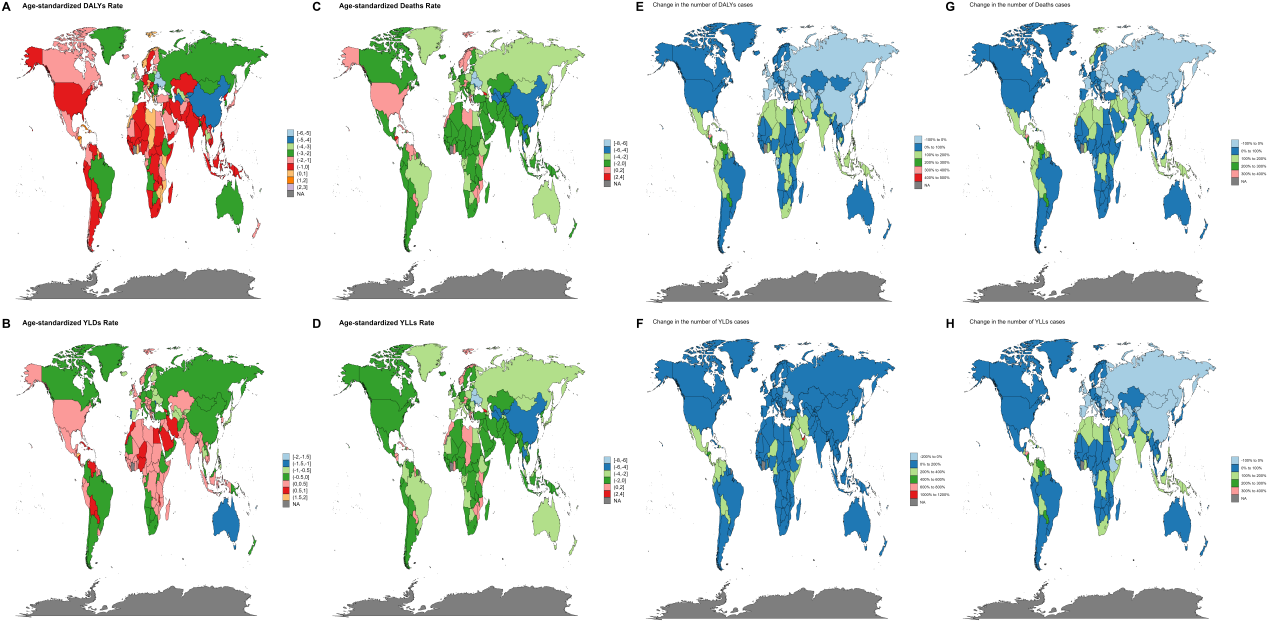
Figure S42.** Trends in the numbers and age-standardized rates of chronic obstructive pulmonary disease attributable to occupational risks-related deaths, DALYs, YLDs, and YLLs globally across countries and territories from 1990 to 2021. Abbreviations: DALYs, disability-adjusted life years; YLDs, years lived with disability; YLLs, years of life lost.

**
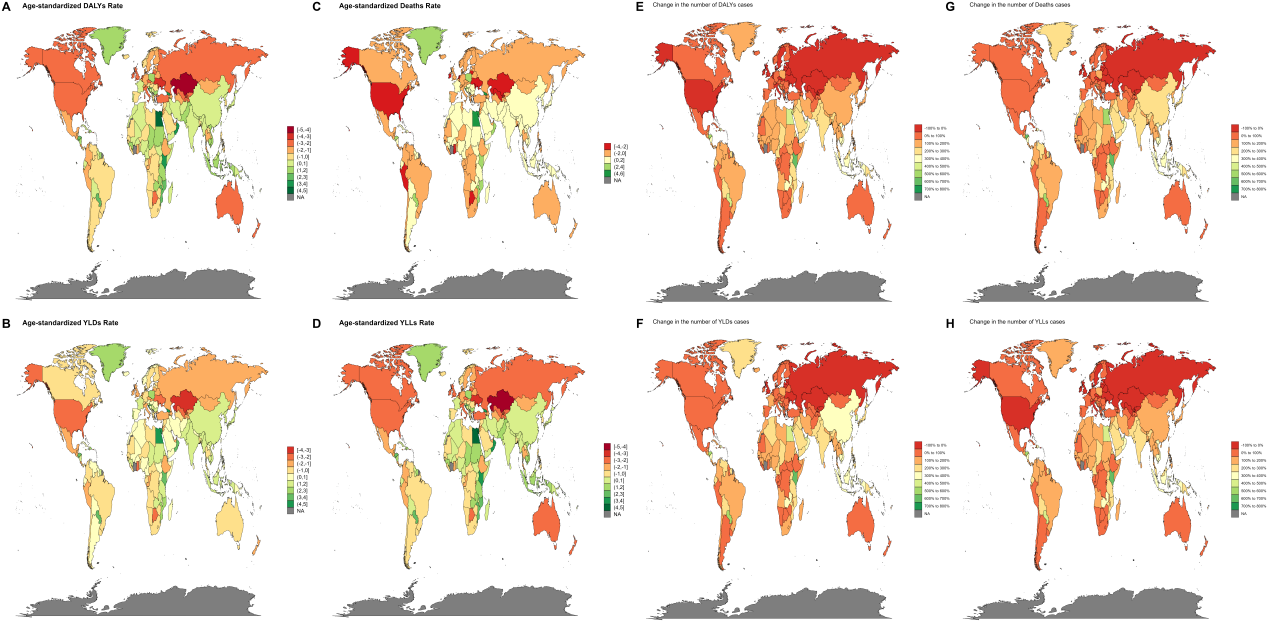
Figure S43.** Trends in the numbers and age-standardized rates of tracheal-bronchus-and-lung cancer attributable to occupational risks-related deaths, DALYs, YLDs, and YLLs globally across countries and territories from 1990 to 2021. Abbreviations: DALYs, disability-adjusted life years; YLDs, years lived with disability; YLLs, years of life lost.

**
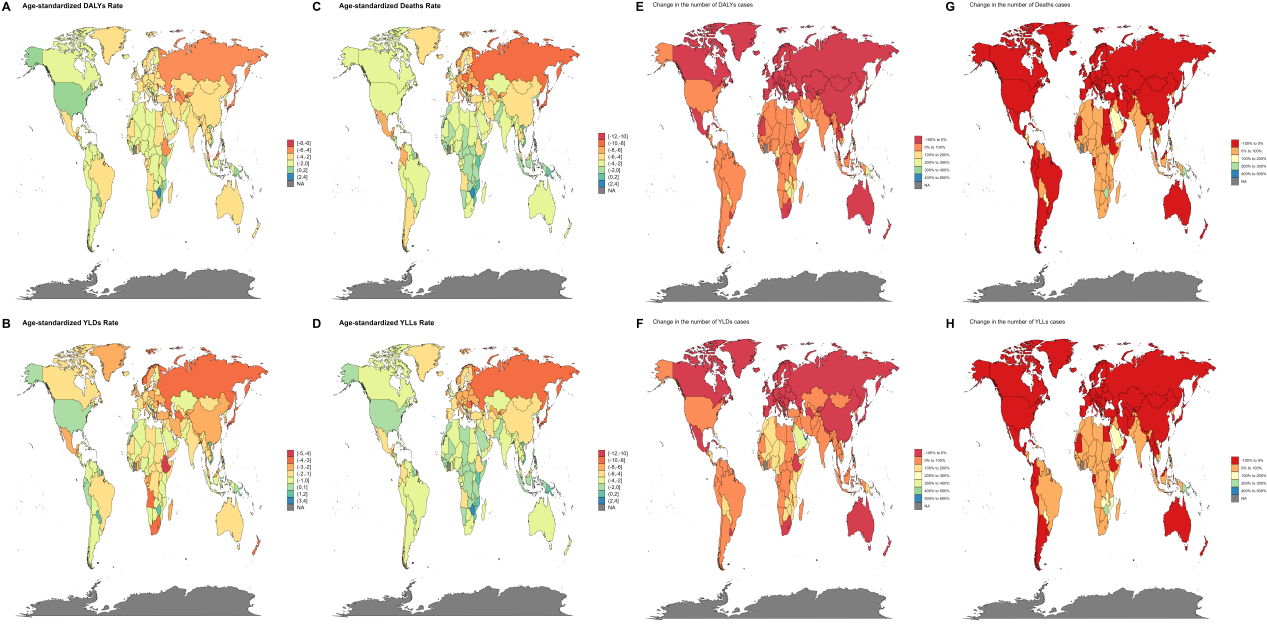
Figure S44.** Trends in the numbers and age-standardized rates of asthma attributable to occupational risks-related deaths, DALYs, YLDs, and YLLs globally across countries and territories from 1990 to 2021. Abbreviations: DALYs, disability-adjusted life years; YLDs, years lived with disability; YLLs, years of life lost.

**
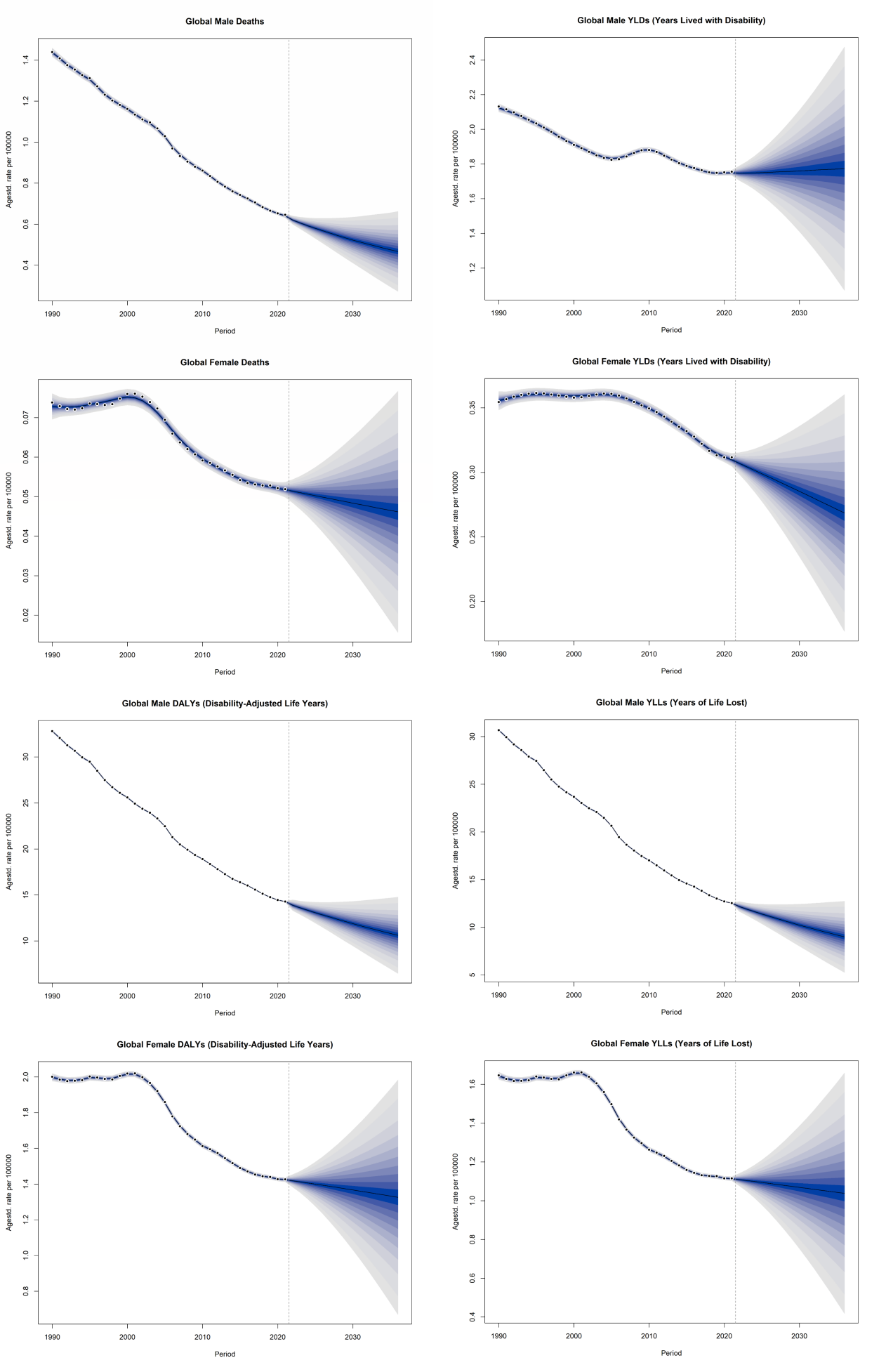
Figure S45.** The predicted results in the pneumoconiosis attributable to occupational risks-related numbers and age-standardized rates of deaths, DALYs, YLDs, and YLLs by sex globally from 2022 to 2050 of the BAPC model. Abbreviations: DALYs, disability-adjusted life years; YLDs, years lived with disability; YLLs, years of life lost; BAPC, Bayesian age-period-cohort.

**
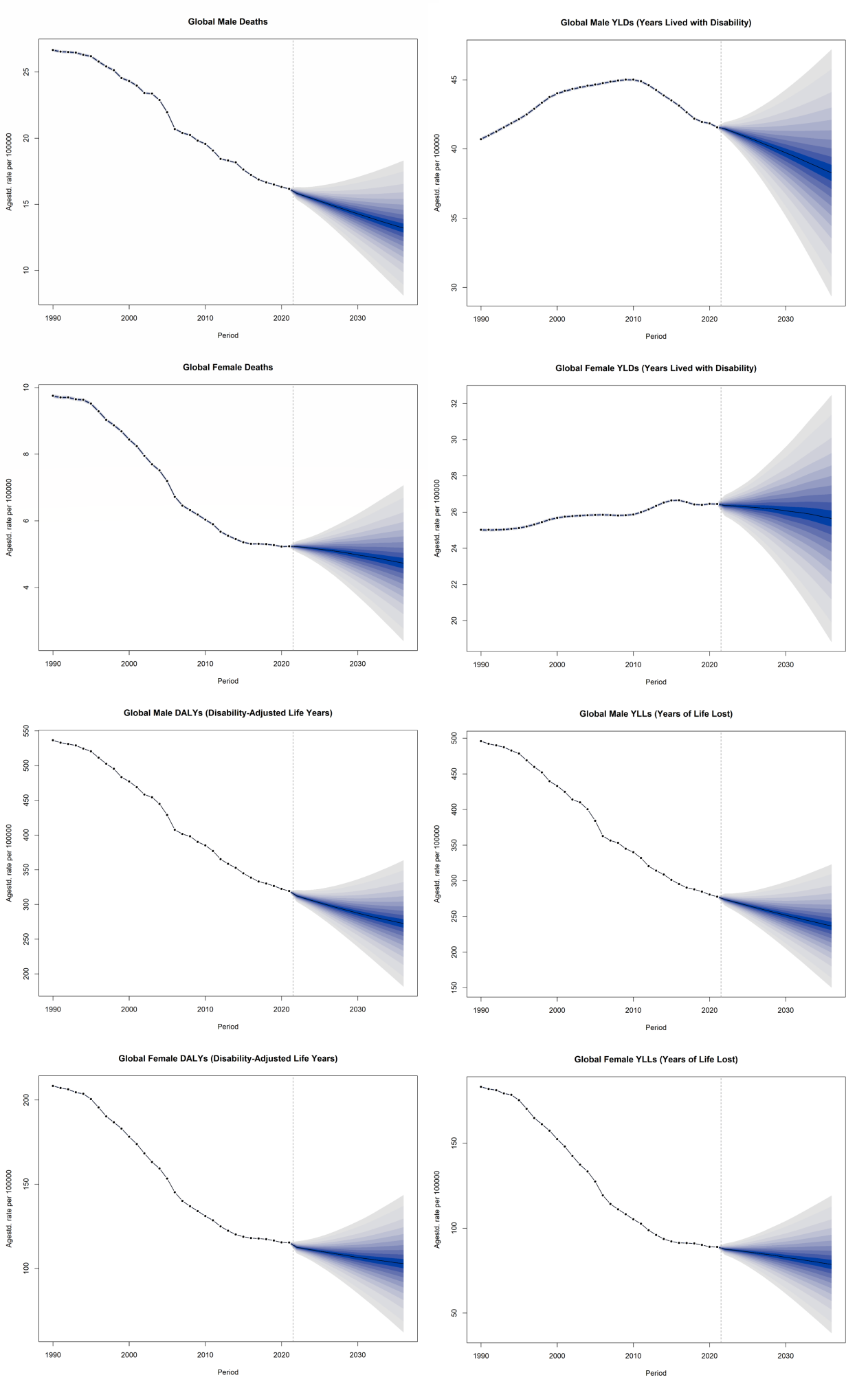
Figure S46.** The predicted results in the chronic obstructive pulmonary disease attributable to occupational risks-related numbers and age-standardized rates of deaths, DALYs, YLDs, and YLLs by sex globally from 2022 to 2050 of the BAPC model. Abbreviations: DALYs, disability-adjusted life years; YLDs, years lived with disability; YLLs, years of life lost; BAPC, Bayesian age-period-cohort.

**
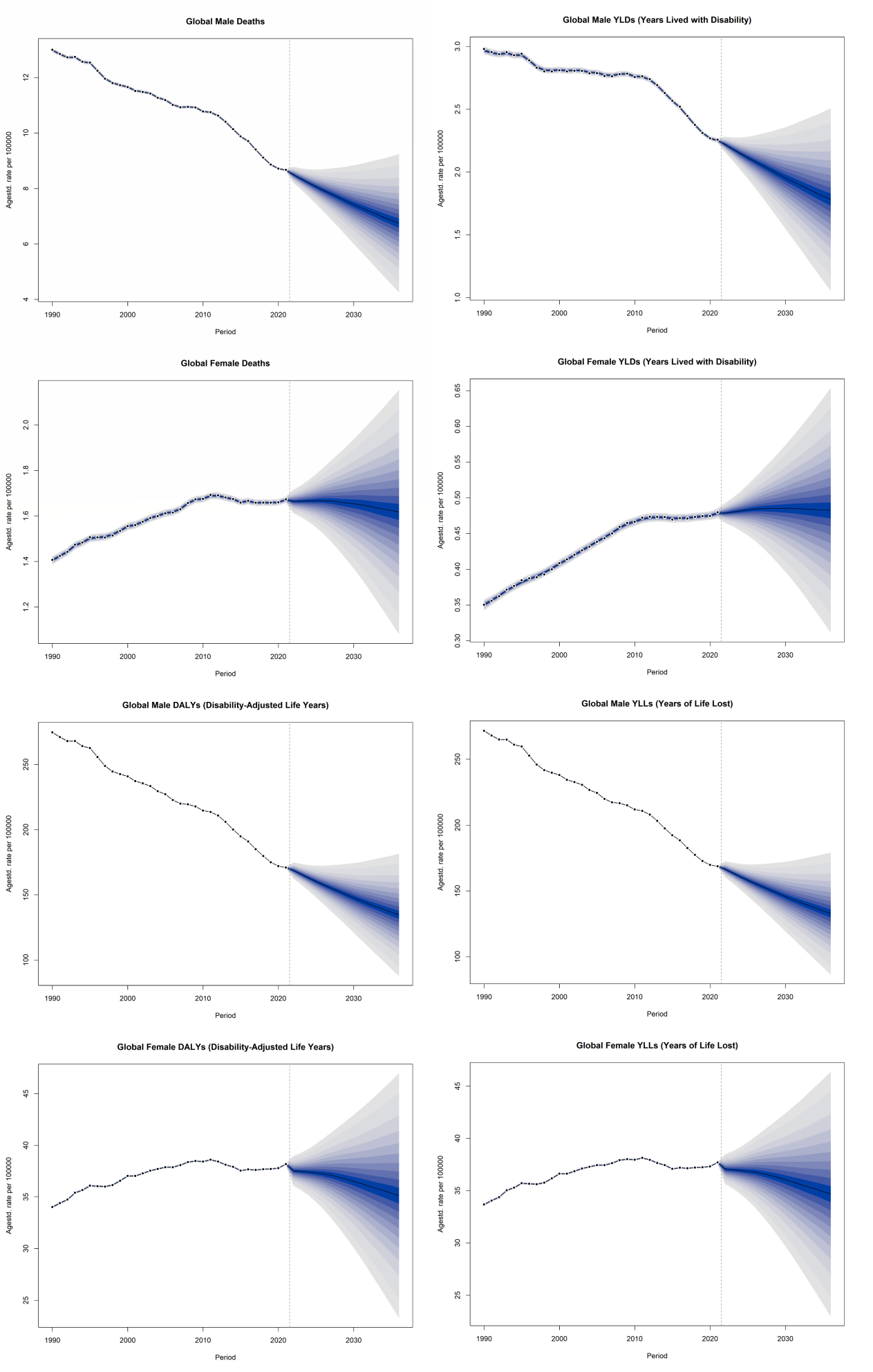
Figure S47.** The predicted results in the tracheal-bronchus-and-lung cancer attributable to occupational risks-related numbers and age-standardized rates of deaths, DALYs, YLDs, and YLLs by sex globally from 2022 to 2050 of the BAPC model. Abbreviations: Abbreviations: DALYs, disability-adjusted life years; YLDs, years lived with disability; YLLs, years of life lost; BAPC, Bayesian age-period-cohort.

**
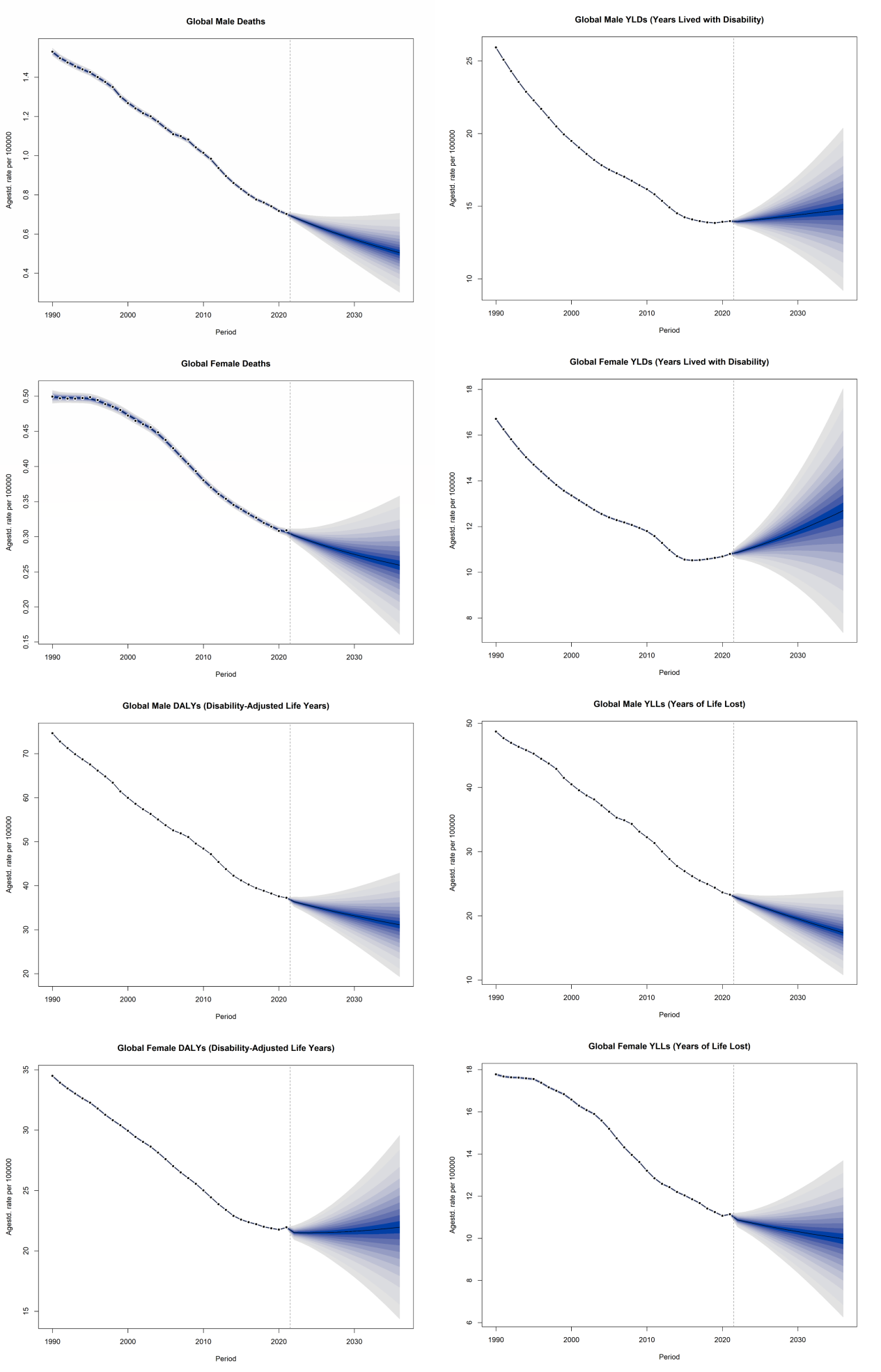
Figure S48.** The predicted results in the asthma attributable to occupational risks-related numbers and age-standardized rates of deaths, DALYs, YLDs, and YLLs by sex globally from 2022 to 2050 of the BAPC model. Abbreviations: Abbreviations: DALYs, disability-adjusted life years; YLDs, years lived with disability; YLLs, years of life lost; BAPC, Bayesian age-period-cohort.

**
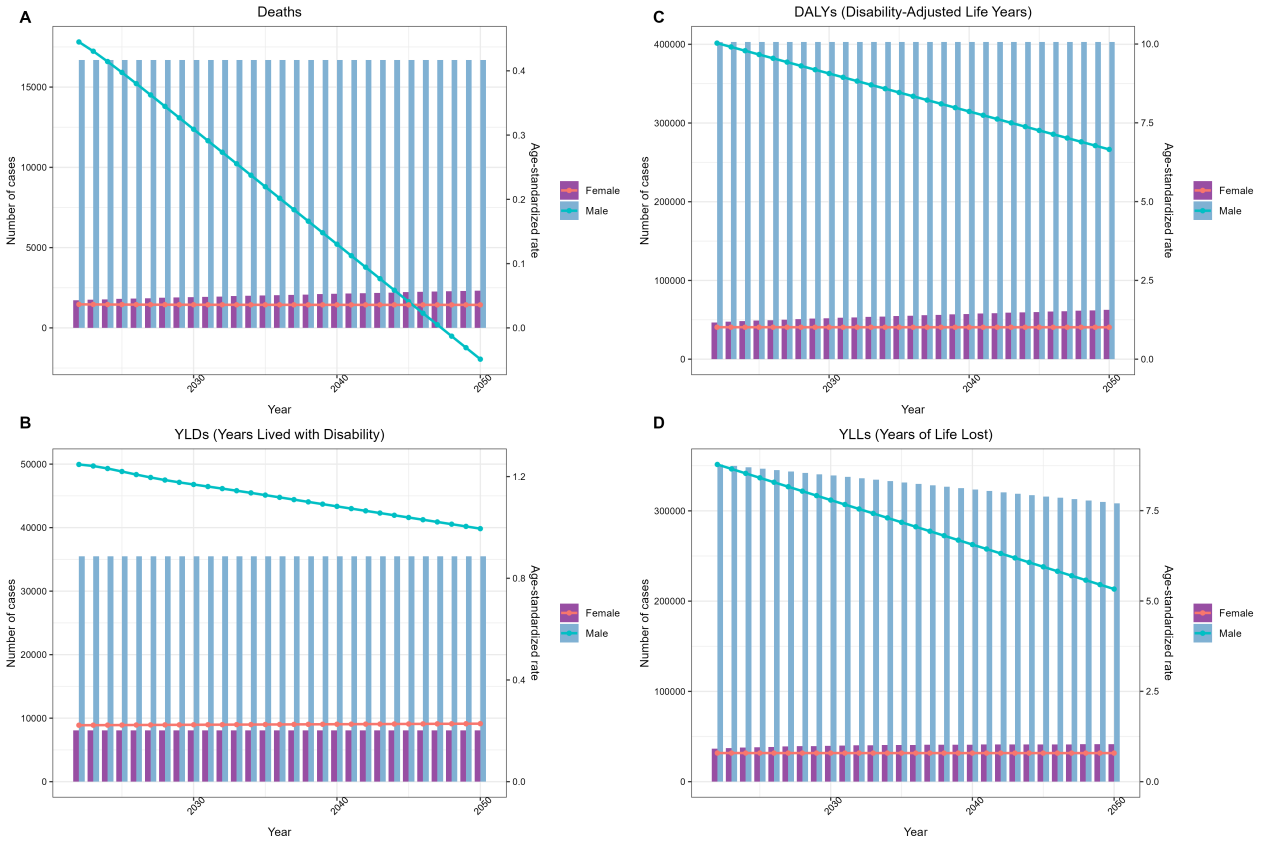
Figure S49.** The predicted results in the pneumoconiosis attributable to occupational risks-related numbers and age-standardized rates of deaths, DALYs, YLDs, and YLLs by sex globally from 2022 to 2050 of the ARIMA model. Abbreviations: DALYs, disability-adjusted life years; YLDs, years lived with disability; YLLs, years of life lost; ARIMA, Autoregressive Integrated Moving Average.

**
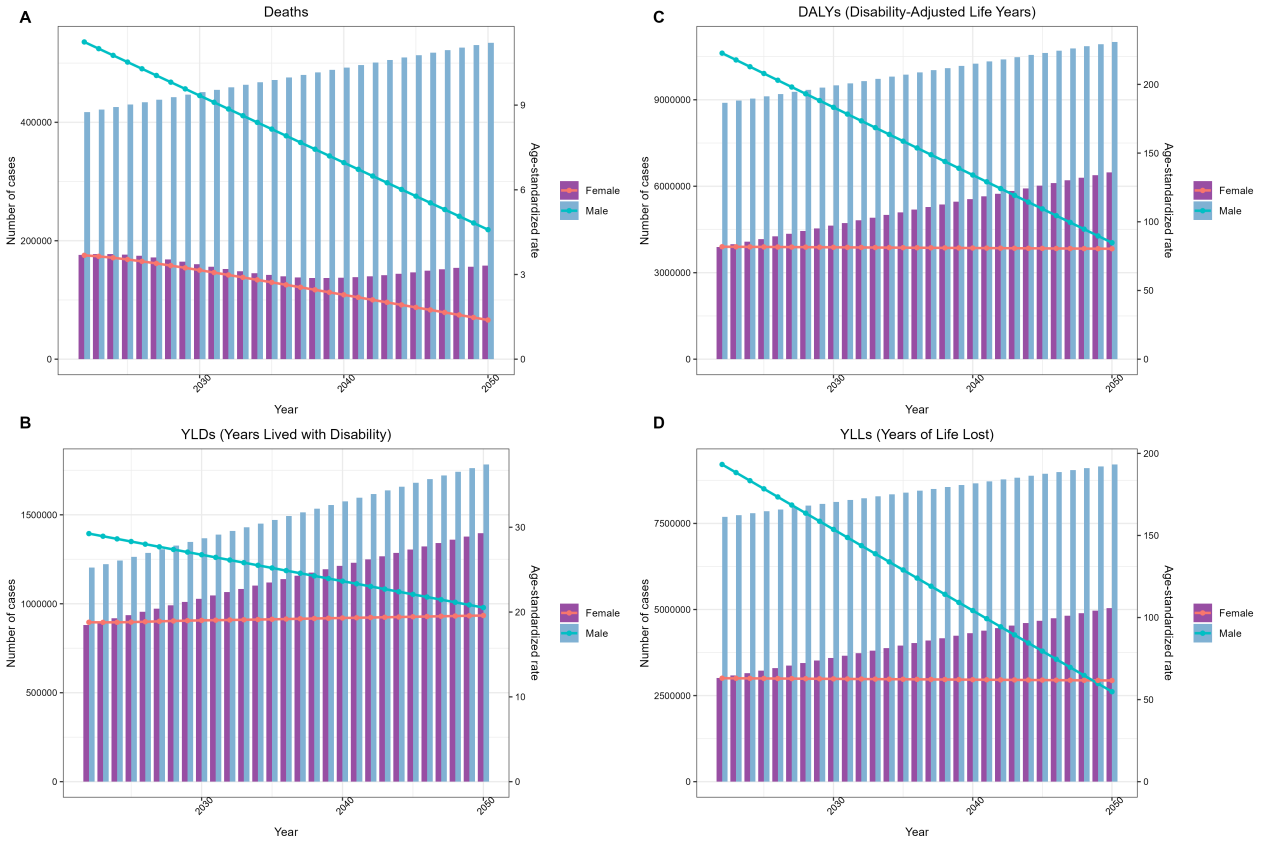
Figure S50.** The predicted results in the chronic obstructive pulmonary disease attributable to occupational risks-related numbers and age-standardized rates of deaths, DALYs, YLDs, and YLLs by sex globally from 2022 to 2050 of the ARIMA model. Abbreviations: DALYs, disability-adjusted life years; YLDs, years lived with disability; YLLs, years of life lost; ARIMA, Autoregressive Integrated Moving Average.

**
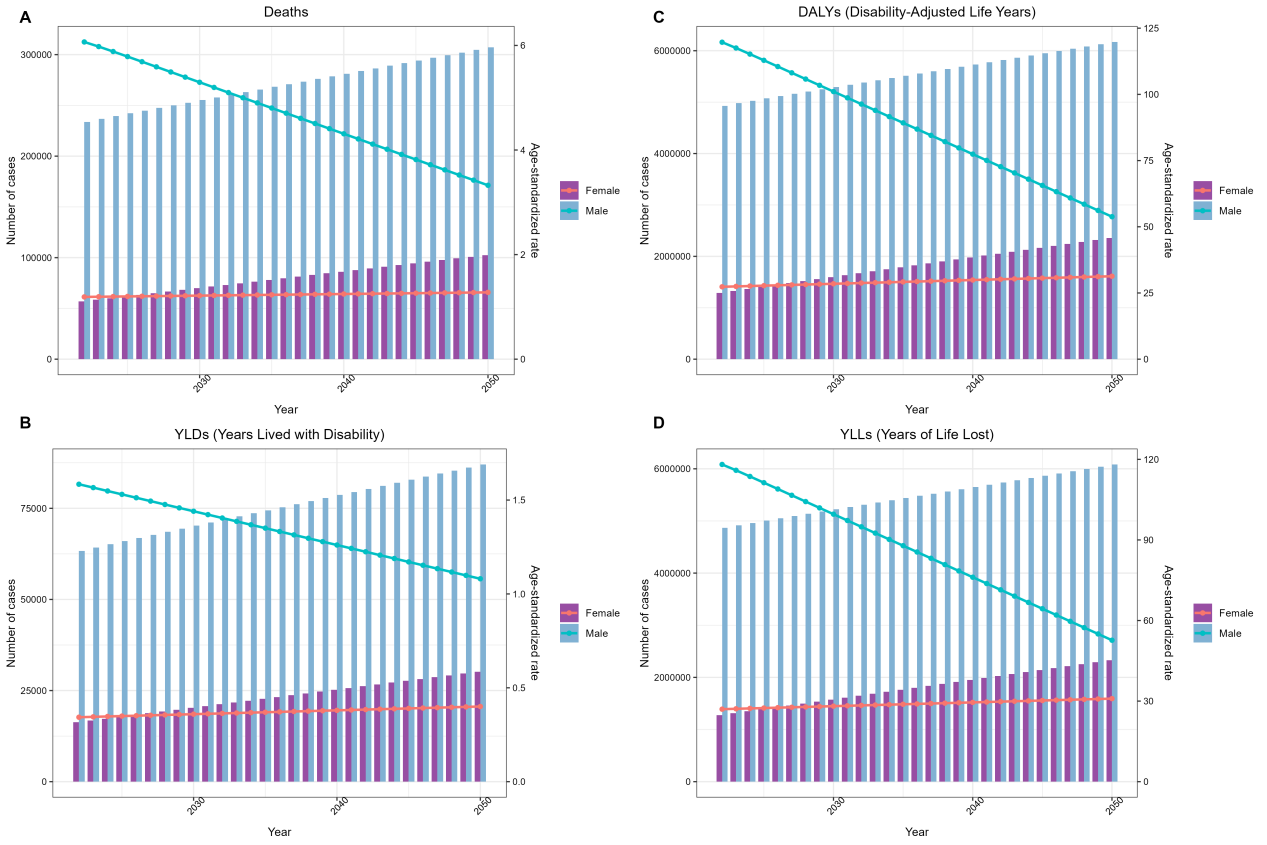
Figure S51.** The predicted results in the tracheal-bronchus-and-lung cancer attributable to occupational risks-related numbers and age-standardized rates of deaths, DALYs, YLDs, and YLLs by sex globally from 2022 to 2050 of the ARIMA model. Abbreviations: Abbreviations: DALYs, disability-adjusted life years; YLDs, years lived with disability; YLLs, years of life lost; ARIMA, Autoregressive Integrated Moving Average.

**
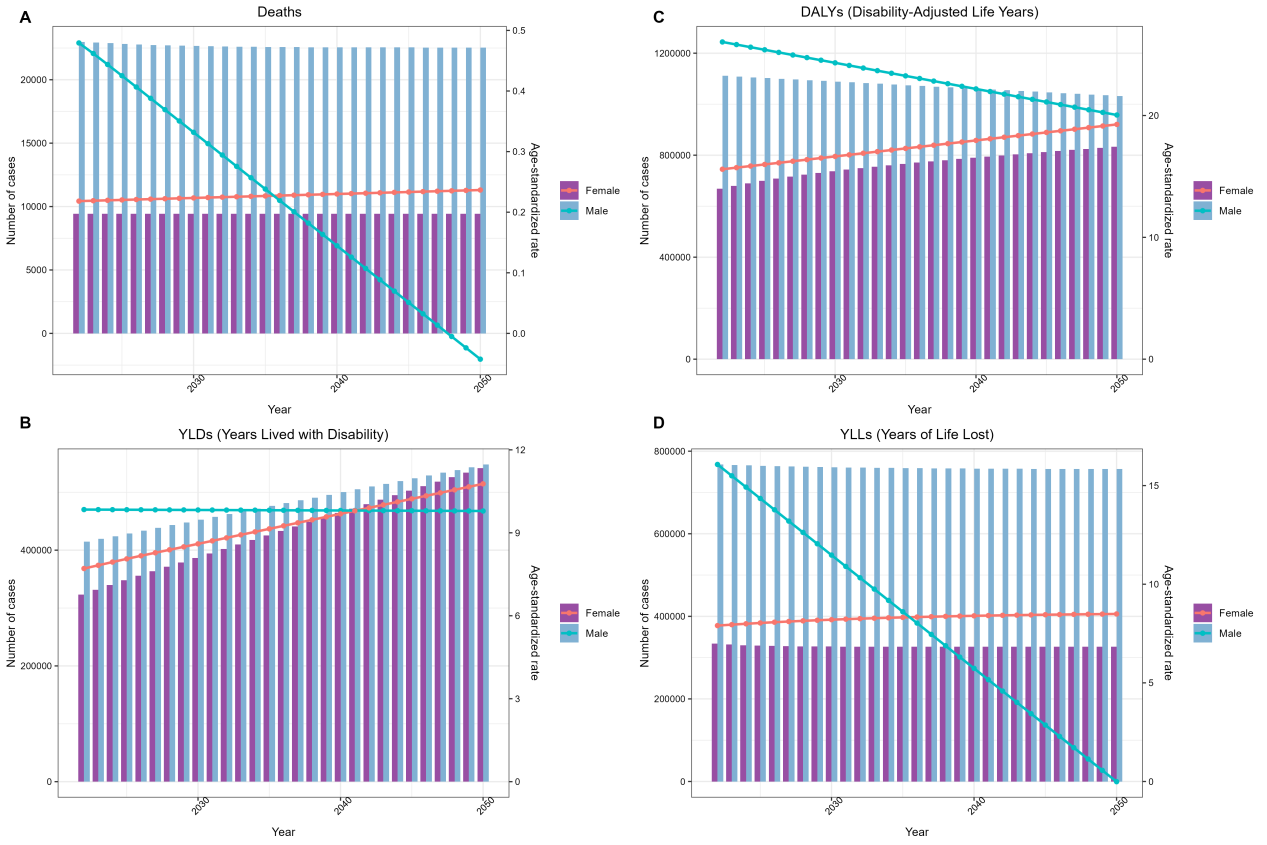
Figure S52.** The predicted results in the asthma attributable to occupational risks-related numbers and age-standardized rates of deaths, DALYs, YLDs, and YLLs by sex globally from 2022 to 2050 of the ARIMA model. Abbreviations: Abbreviations: DALYs, disability-adjusted life years; YLDs, years lived with disability; YLLs, years of life lost; ARIMA, Autoregressive Integrated Moving Average.

**
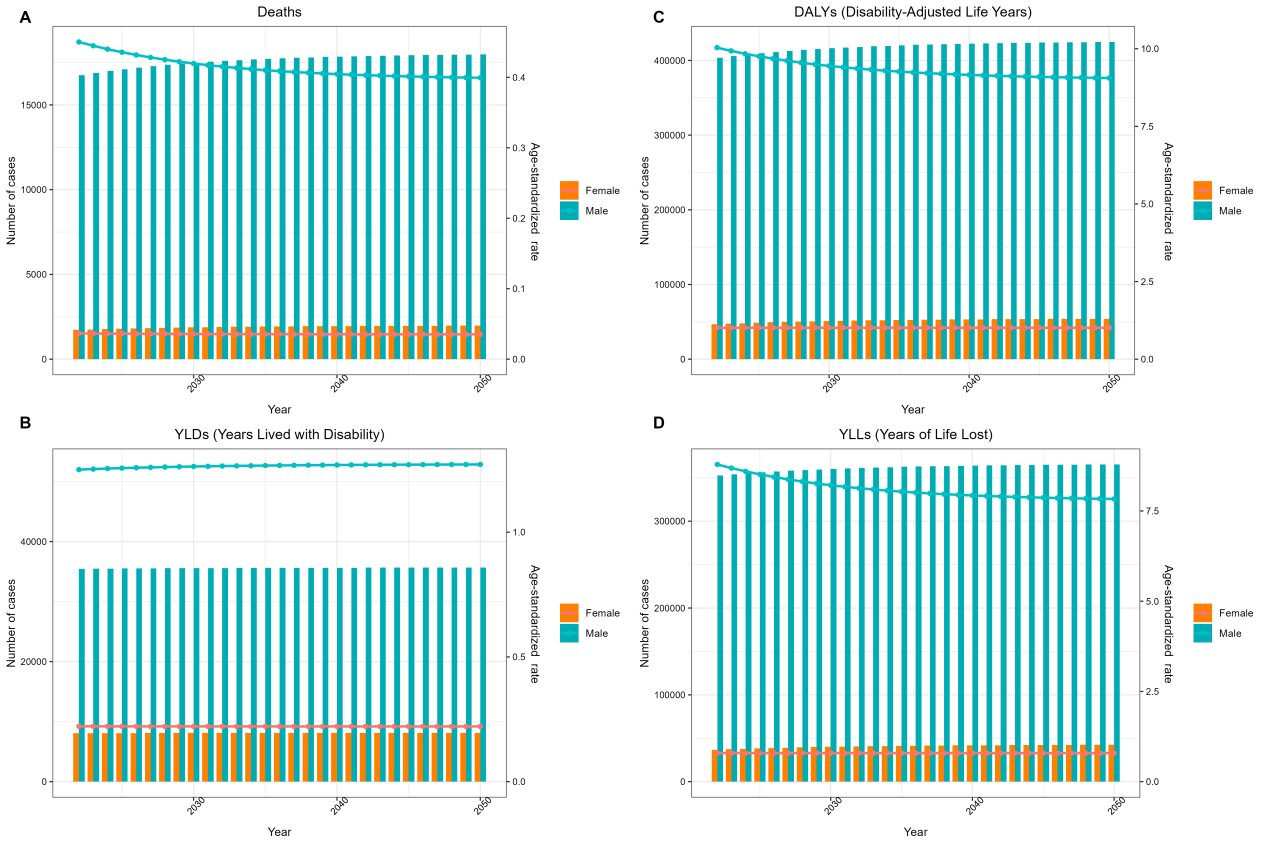
Figure S53.** The predicted results in the pneumoconiosis attributable to occupational risks-related numbers and age-standardized rates of deaths, DALYs, YLDs, and YLLs by sex globally from 2022 to 2050 of the ES model. Abbreviations: Abbreviations: DALYs, disability-adjusted life years; YLDs, years lived with disability; YLLs, years of life lost; ES, Exponential Smoothing.

**
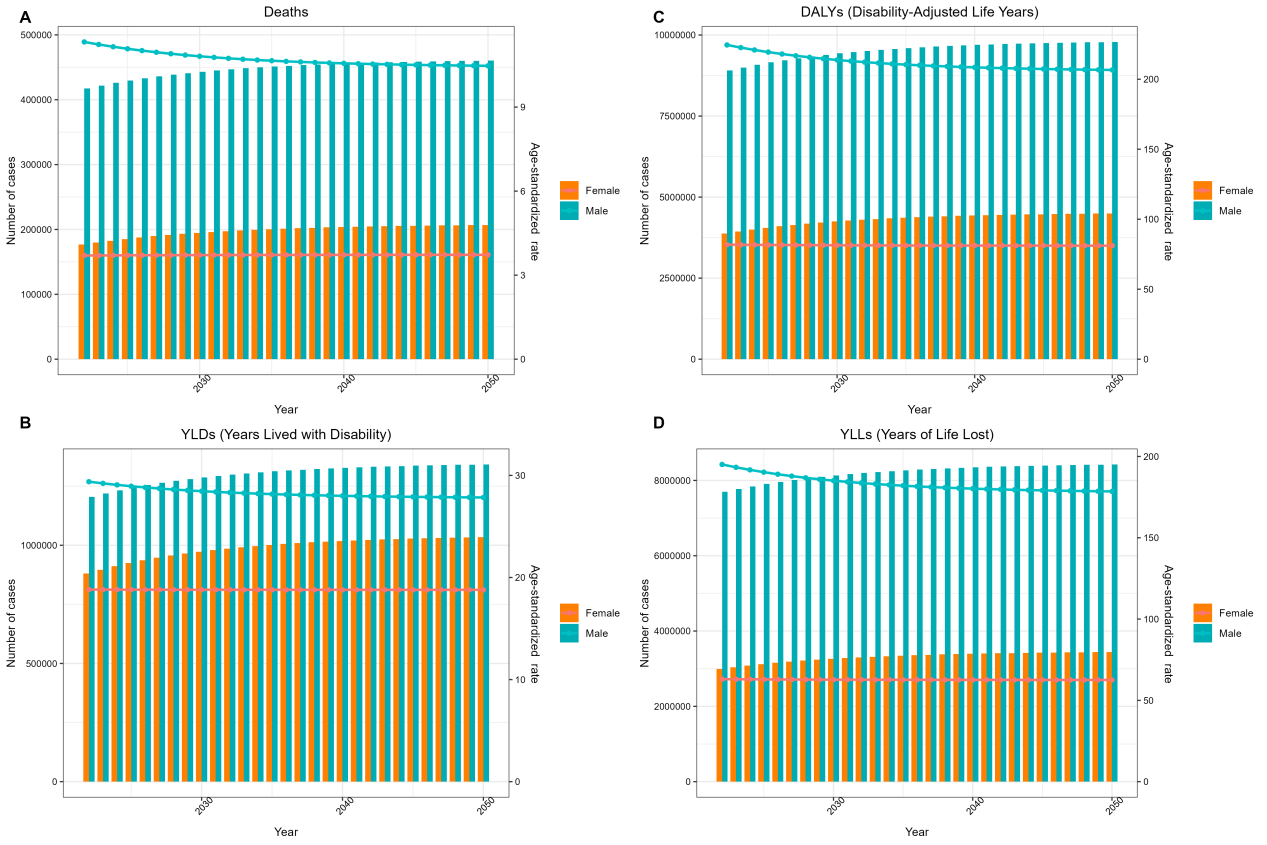
Figure S54.** The predicted results in the chronic obstructive pulmonary disease attributable to occupational risks-related numbers and age-standardized rates of deaths, DALYs, YLDs, and YLLs by sex globally from 2022 to 2050 of the ES model. Abbreviations: Abbreviations: DALYs, disability-adjusted life years; YLDs, years lived with disability; YLLs, years of life lost; ES, Exponential Smoothing.

**
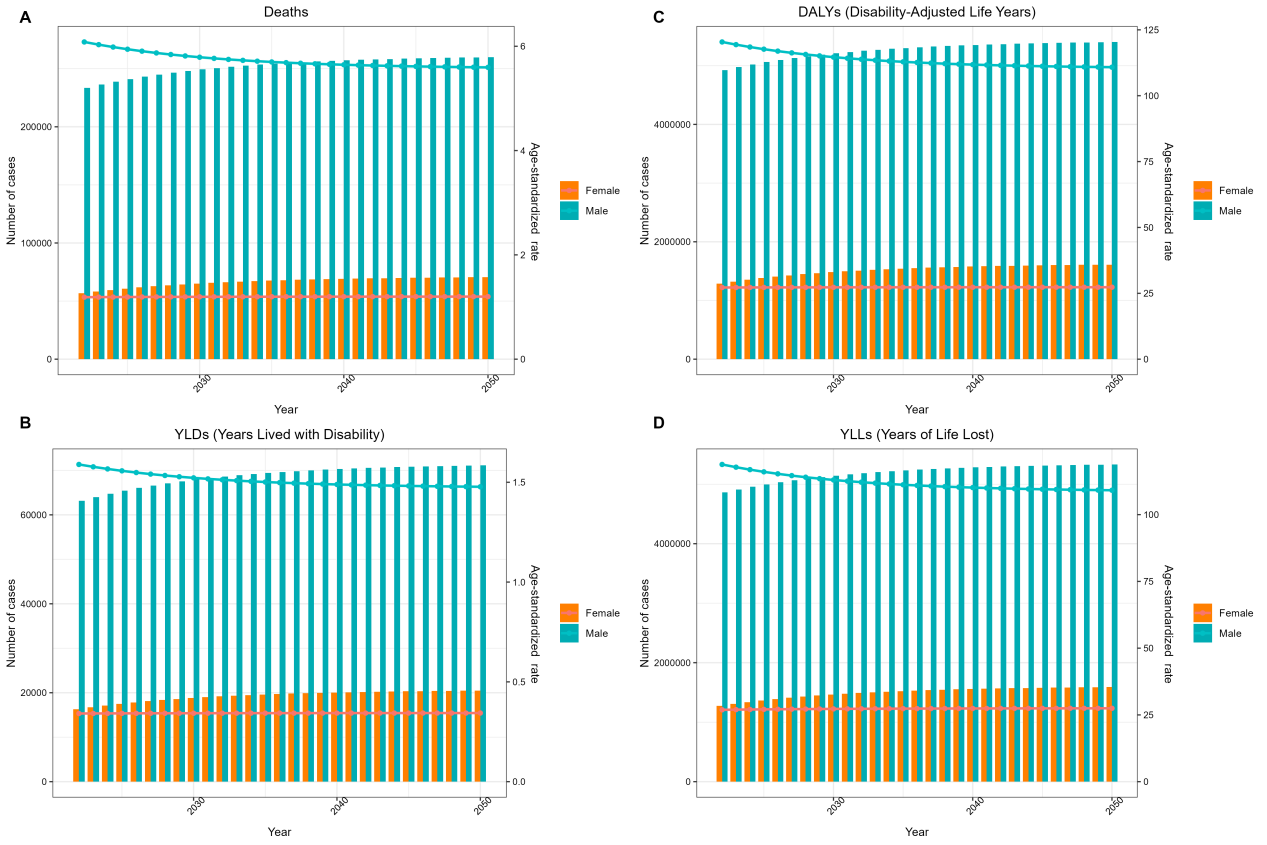
Figure S55.** The predicted results in the tracheal-bronchus-and-lung cancer attributable to occupational risks-related numbers and age-standardized rates of deaths, DALYs, YLDs, and YLLs by sex globally from 2022 to 2050 of the ES model. Abbreviations: DALYs, disability-adjusted life years; YLDs, years lived with disability; YLLs, years of life lost; ES, Exponential Smoothing.

**
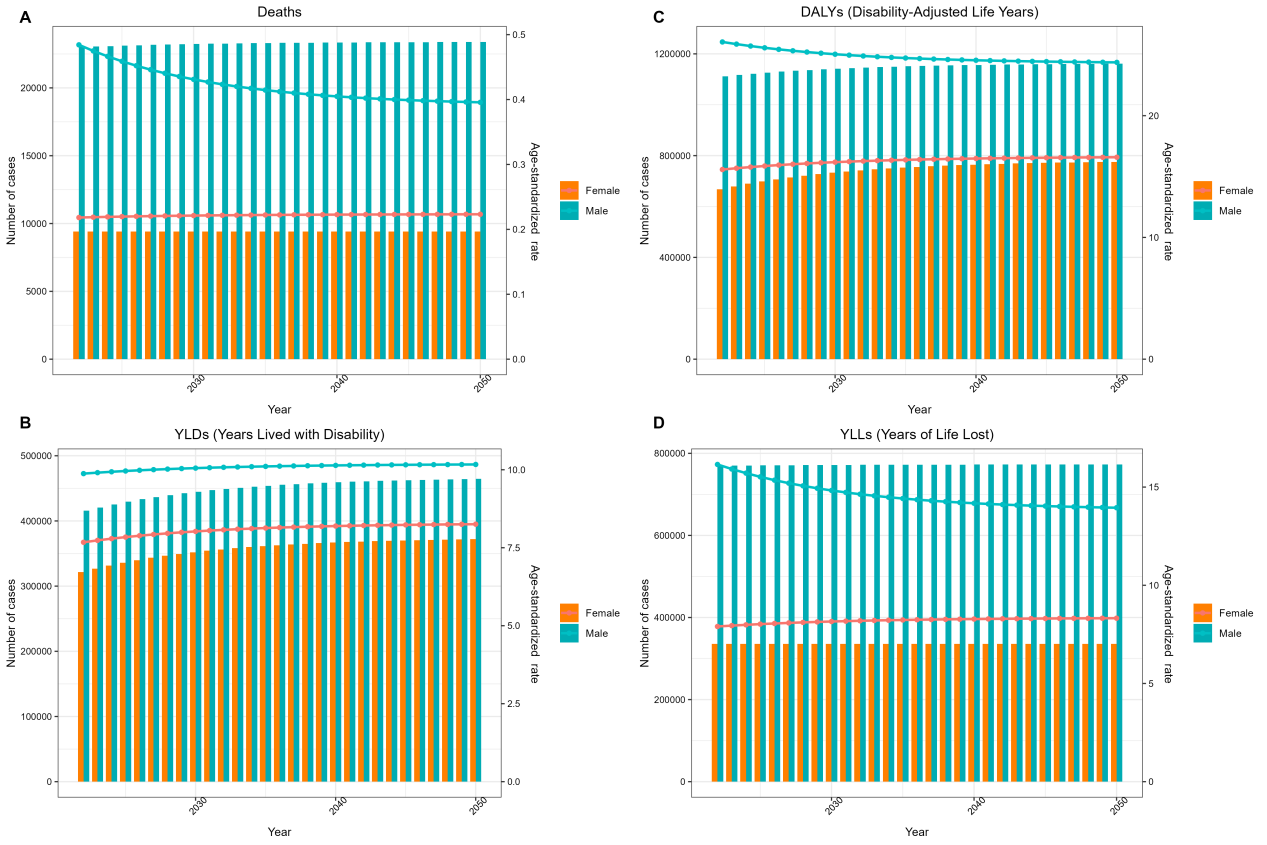
Figure S56.** The predicted results in the asthma attributable to occupational risks-related numbers and age-standardized rates of deaths, DALYs, YLDs, and YLLs by sex globally from 2022 to 2050 of the ES model. Abbreviations: DALYs, disability-adjusted life years; YLDs, years lived with disability; YLLs, years of life lost; ES, Exponential Smoothing.

**
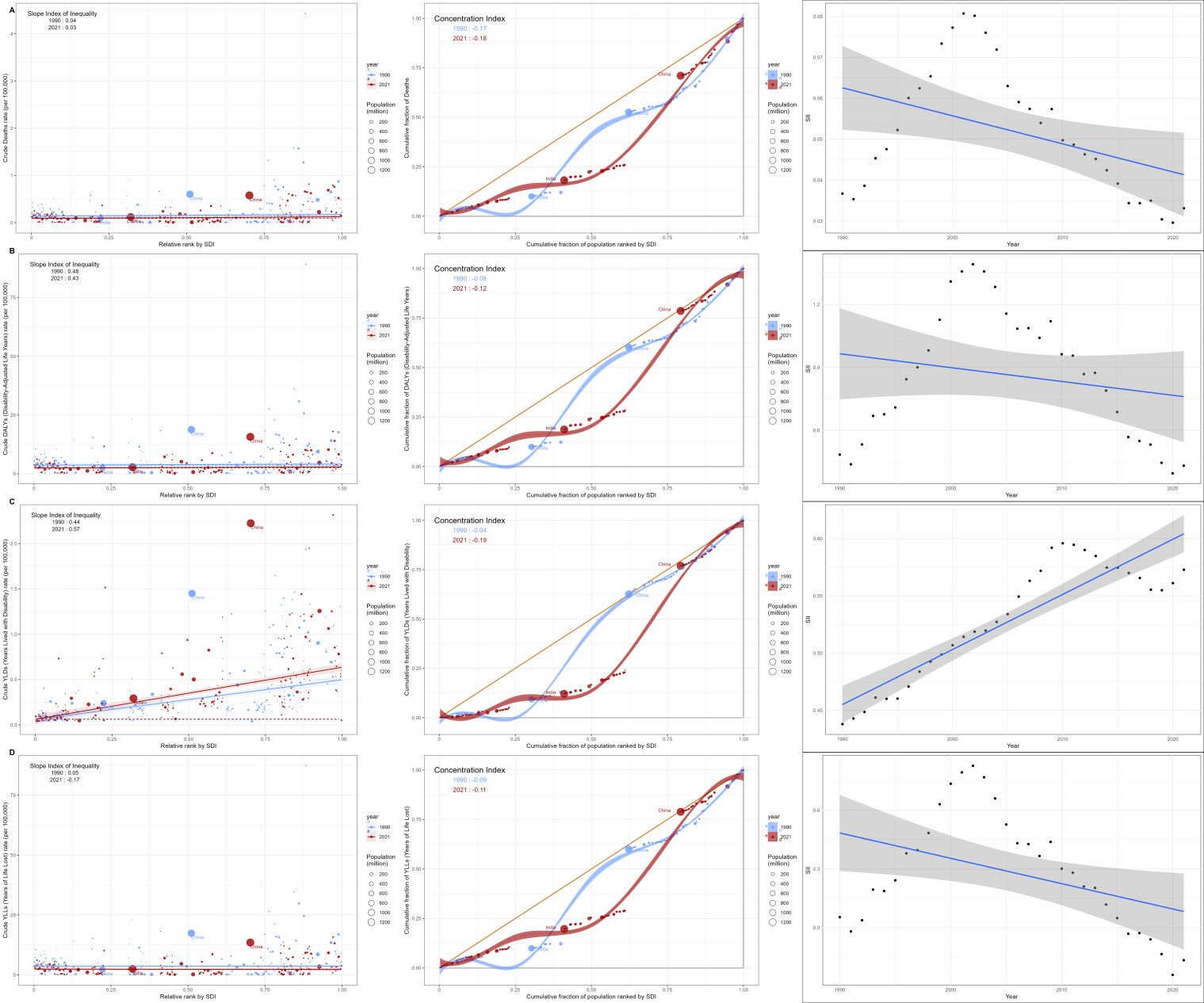
Figure S57.** The results of healthy inequality analysis for pneumoconiosis attributable to occupational risks.

**
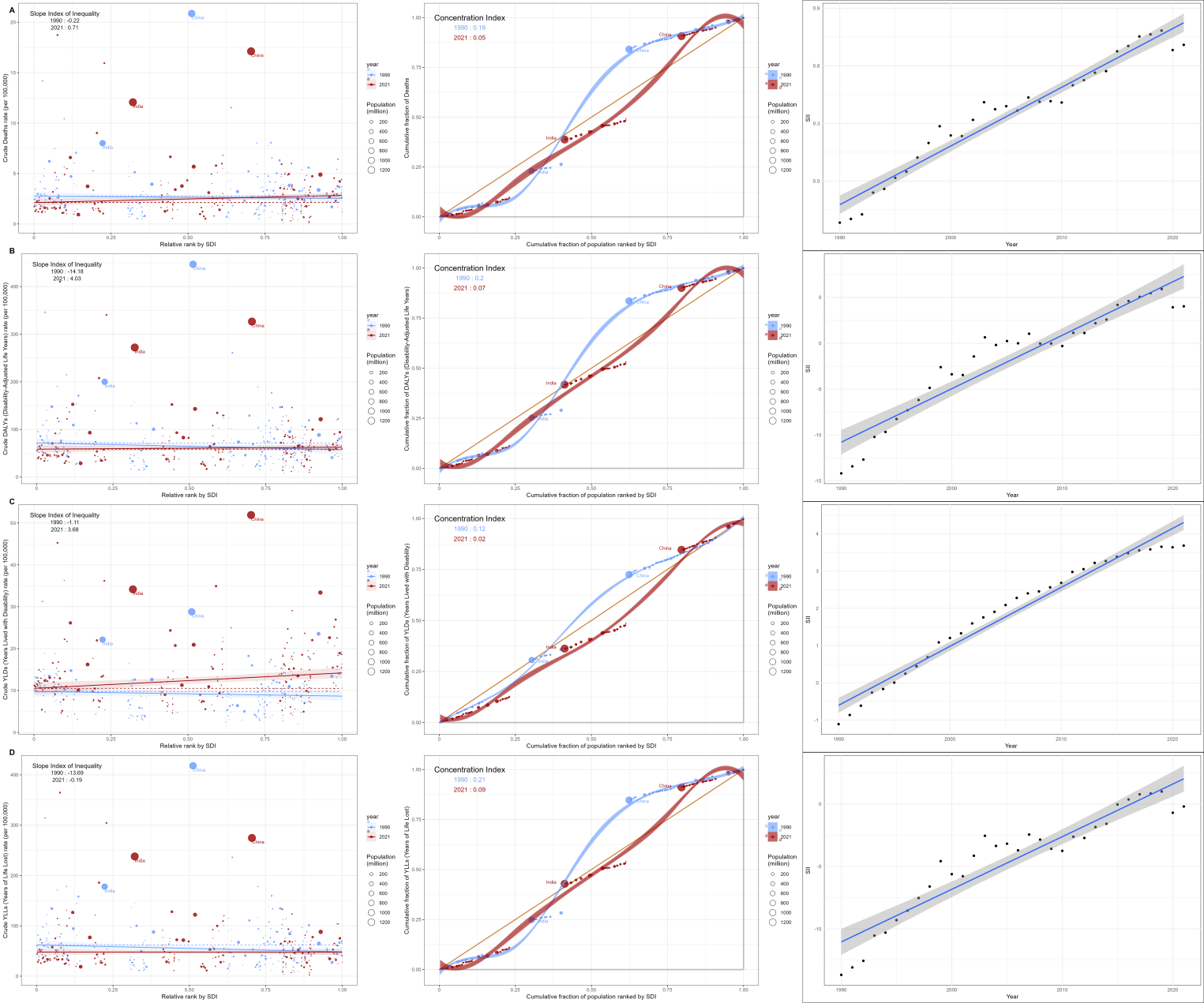
Figure S58.** The results of healthy inequality analysis for chronic obstructive pulmonary disease attributable to occupational risks.

**
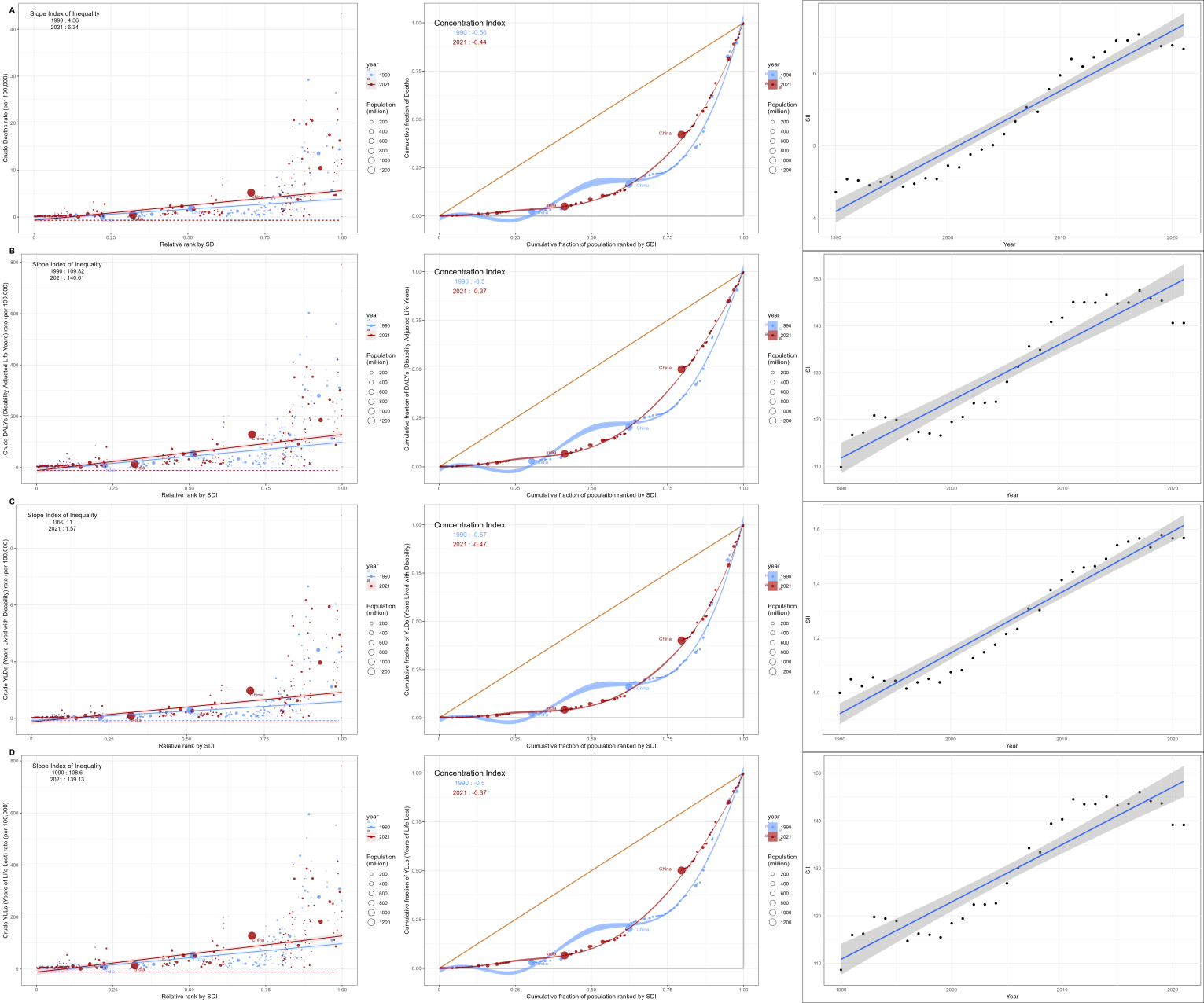
Figure S59.** The results of healthy inequality analysis for tracheal-bronchus-and-lung cancer attributable to occupational risks.

**
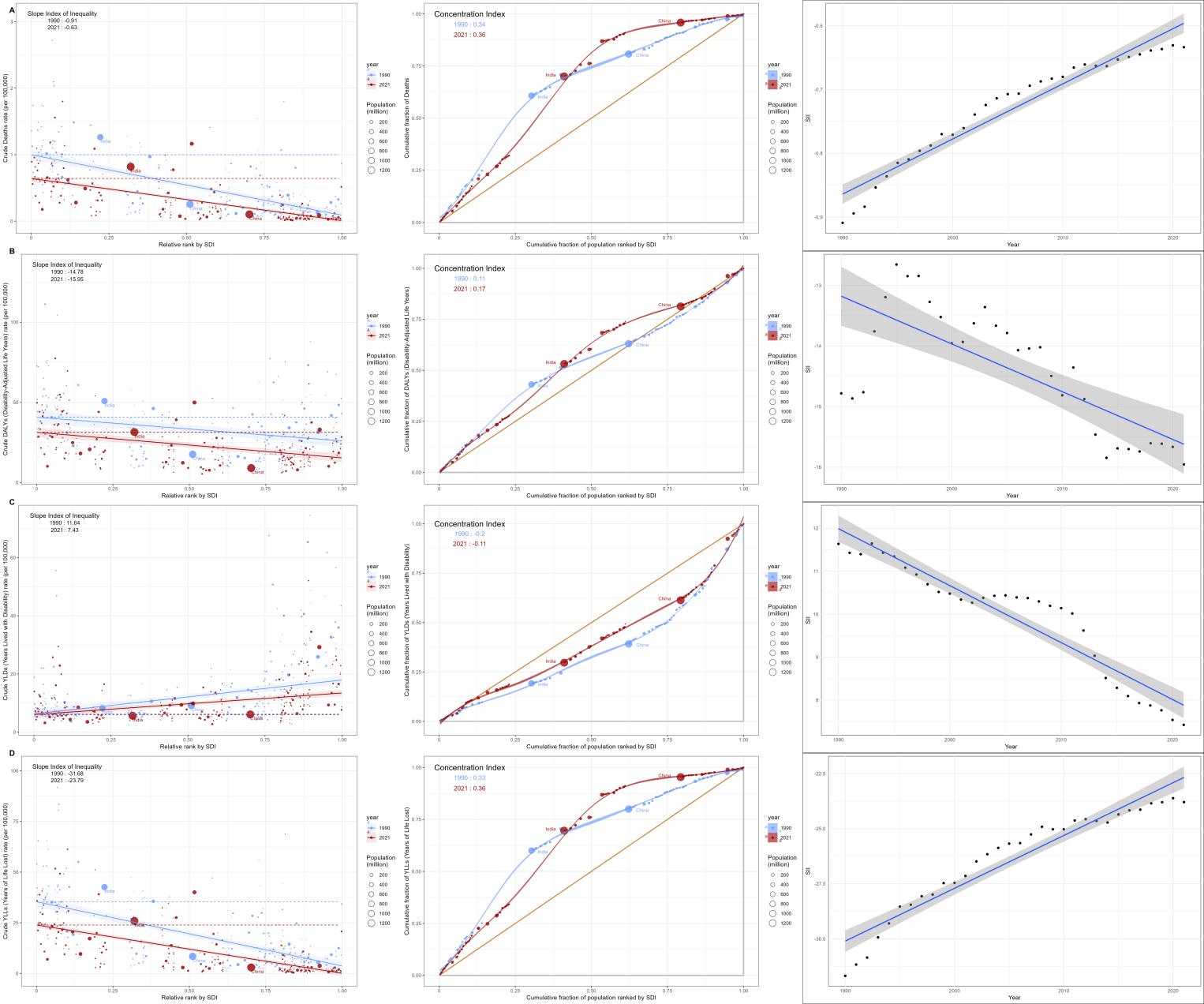
Figure S60.** The results of healthy inequality analysis for asthma attributable to occupational risks.
